# Synthesis of Novel Shikonin Derivatives and Pharmacological Effects of Cyclopropylacetylshikonin on Melanoma Cells

**DOI:** 10.3390/molecules23112820

**Published:** 2018-10-30

**Authors:** Christin Durchschein, Antje Hufner, Beate Rinner, Alexander Stallinger, Alexander Deutsch, Birgit Lohberger, Rudolf Bauer, Nadine Kretschmer

**Affiliations:** 1 Institute of Pharmaceutical Sciences, Department of Pharmacognosy, University of Graz, Universitaetsplatz 4, 8010 Graz, Austria; christin.durchschein@uni-graz.at (C.D.); nadine.kretschmer@uni-graz.at (N.K.); 2 Institute of Pharmaceutical Sciences, Department of Pharmaceutical Chemistry, University of Graz, Universitaetsplatz 1, 8010 Graz, Austria; antje.huefner@uni-graz.at; 3 Division of Biomedical Research, Medical University of Graz, Roseggerweg 48, 8036 Graz, Austria; beate.rinner@medunigraz.at (B.R.); alexander.stallinger@medunigraz.at (A.S.); 4 Division of Hematology, Medical University of Graz, Auenbruggerplatz 15, 8036 Graz, Austria; alexander.deutsch@medunigraz.at; 5 Department of Orthopedics and Trauma, Medical University Graz, Auenbruggerplatz 5, 8036 Graz, Austria; birgit.lohberger@medunigraz.at

**Keywords:** shikonin derivatives, cyclopropylacetylshikonin, apoptosis, melanoma

## Abstract

Despite much research in the last centuries, treatment of malignant melanoma is still challenging because of its mostly unnoticeable metastatic spreading and aggressive growth rate. Therefore, the discovery of novel drug leads is an important goal. In a previous study, we have isolated several shikonin derivatives from the roots of *Onosma paniculata* Bureau & Franchet (Boraginaceae) which evolved as promising anticancer candidates. *β*,*β*-Dimethylacrylshikonin (**1**) was the most cytotoxic derivative and exhibited strong tumor growth inhibitory activity, in particular, towards melanoma cells. In this study, we synthesized eighteen novel shikonin derivatives in order to obtain compounds which exhibit a higher cytotoxicity than **1**. We investigated their cytotoxic potential against various melanoma cell lines and juvenile skin fibroblasts. The most active compound was (*R*)-1-(1,4-dihydro-5,8-dihydroxy-1,4-dioxonaphthalen-2-yl)-4-methylpent-3-enyl cyclopropylacetate (cyclopropylacetylshikonin) (**6**). It revealed significant stronger tumor growth inhibitory activity towards two melanoma cell lines derived from metastatic lesions (WM164 and MUG-Mel2). Further investigations have shown that **6** induced apoptosis caspase-dependently, increased the protein levels of cleaved PARP, and led to double-stranded DNA breaks as shown by phosphorylation of H2AX. Cell membrane damage and cell cycle arrest were not observed.

## 1. Introduction

Skin cancer is a major public health concern all over the world. According to the WHO, one of three diagnosed cancers is a skin cancer. Melanoma is the most dangerous type of skin cancer because of its often unnoticeable metastatic spreading and aggressive growth rate. Only approx. 4% of human skin cancers are melanoma but cause around 80% of all skin cancer deaths and mortality rates are increasing worldwide [1]. In early stages, skin-localized melanomas are complete removed by local surgery. However, advanced metastatic melanomas remain a challenge in treatment. Although progress in elucidating the genetic and molecular aberrations of this disease has been accomplished in recent years and novel drugs, such as vemurafenib (BRAF inhibitor), ipilimumab (anti-CTLA-4 monoclonal antibody), and nivolumab (anti-PD-1 antibody), have been approved, we are far from understanding the pathogenesis of this disease precisely [2]. In addition, it has to be remembered that medicinal treatments still have limited efficacy due to resistances of tumors, low response rates as well as adverse side effects [3]. In the development of new anticancer drugs, natural products have always played an important role. Approximately 87% of all approved small molecules anticancer drugs are natural products or are derived from them [4]. Famous natural products in this area are vinca alkaloids isolated from *Catharantus roseus* G. Don, like vinblastine and vincristine, anthracyclines from *Streptomyces* bacteria such as doxorubicin, and taxanes from different *Taxus* species, like paclitaxel and docetaxel. During the last decades, many studies have demonstrated the broad pharmacological spectrum of shikonin and derivatives, natural naphthoquinones derived from the roots of Chinese herbs such as *Arnebia euchroma* (Royle) I. M. Johnston, *Lithospermum erythrorhizon* Siebold & Zuccarini, or *Onosma paniculata* Bureau & Franchet. Traditionally, these roots are used to treat several diseases including cancer. In previous studies, roots of *O. paniculata* were phytochemically and pharmacologically investigated and emerged as promising research objects [5,6,7,8]. We were able to isolate several shikonin derivatives and investigated their effects on various tumor cell lines including leukemia, medullary thyroid carcinoma, glioblastoma, colon cancer, breast cancer, and melanoma [5,6,7,8]. Overall, *β*,*β*-dimethylacrylshikonin (**1**) was the most cytotoxic compound of all isolated derivatives. Interestingly, it exhibited the strongest cytotoxic activity against several melanoma cell lines [8]. Therefore, we decided to concentrate our studies on the effects in melanoma cells. We could show that it led to apoptosis induction by caspase activation as well as cell cycle arrest [8]. In general, the anticancer effects of shikonin derivatives are largely attributed to the induction of cell cycle arrest, apoptosis, and/or necroptosis and the inhibition of migration and invasion [8,9,10,11]. In this study, we are addressing the question of whether the structure of **1** can be modified to find derivatives which are more cytotoxic. In previous investigations, we confirmed that the naphtoquinone scaffold is more crucial for the entire activity than the side chain [12]. Moreover, the presence of phenolic hydroxyl groups increases the activity [13,14] and the side chain seems to modify the entire activity [15,16,17]. We now have synthesized eighteen novel shikonin derivatives and evaluated their cytotoxic effects against various melanoma cell lines and skin fibroblasts. Finally, the pharmacological effects of the most active derivative were investigated in more detail.

## 2. Results and Discussion 

### 2.1. Synthesis of Novel Shikonin Derivatives

Shikonin derivatives possess strong growth inhibitory activity towards various cancer cell lines [18,19,20]. In a previous investigation, we confirmed that the naphtoquinone scaffold is more crucial for the entire activity than the side chain [12]. However, there are indications that the side chain influences the entire activity [12,15,16,17]. The hydroxyl groups at the naphthazarin scaffold are necessary for maintaining its drug-like property. Also all known anthracycline antitumor antibiotics and mitoxantrone include a quinizarin skeleton with free hydroxyl groups. These might be important for DNA binding and cell or tissue bioavailability [21]. Therefore, we decided to modify the side chain of **1** which was the most cytotoxic derivative in a previous study [8]. To keep close to our lead compound **1**, we formally connected the two methyls of dimethylacylate with one to four methylene groups resulting in the corresponding cycloalkylideneacetates **2** to **5**. The influence of the double bond was investigated by preparing cycloalkylacetates **6** to **9** and cyclohexenylacetate **10**. To study the effect of the spacing CH resp. CH_2_ group, cyclopropyl to cyclohexylcarboxylates **11** to **14** together with the cyclohexenylcarboxylates **15** and **16** were synthesized. 3-methylcyclopropylcarboxylate **17** and bicyclic derivatives **18**, **19**, and **20** were produced to analyze the space filling of an additional methylene moiety (Table 1). 

**Scheme 1:**
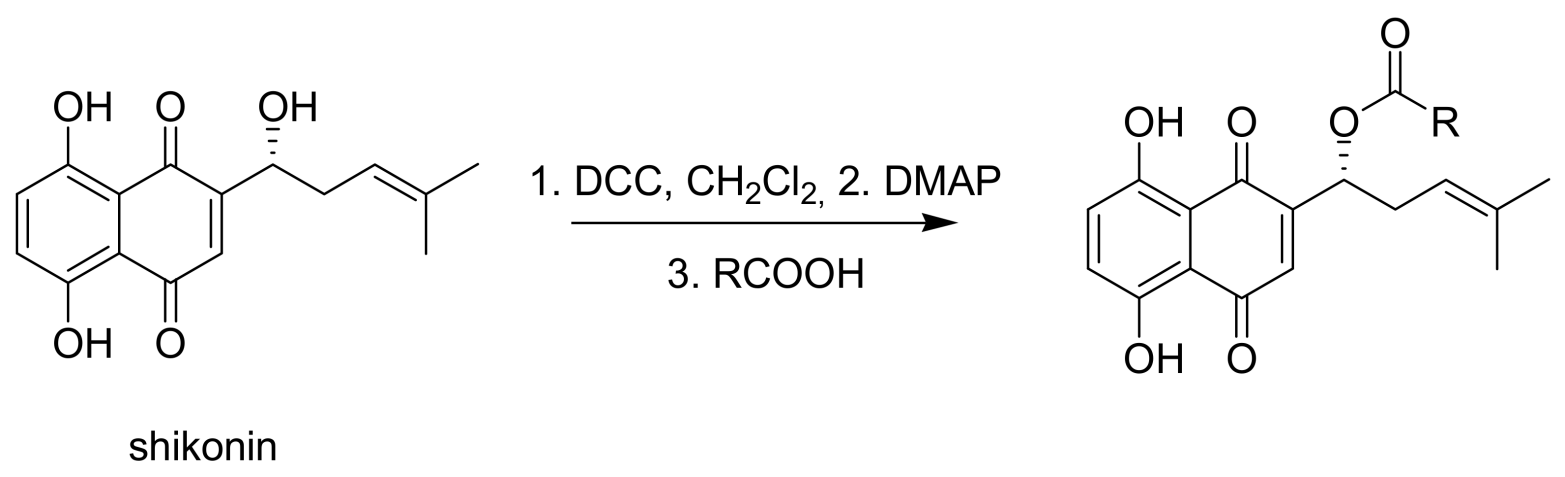
Acylation of shikonin.

**Scheme 2:**
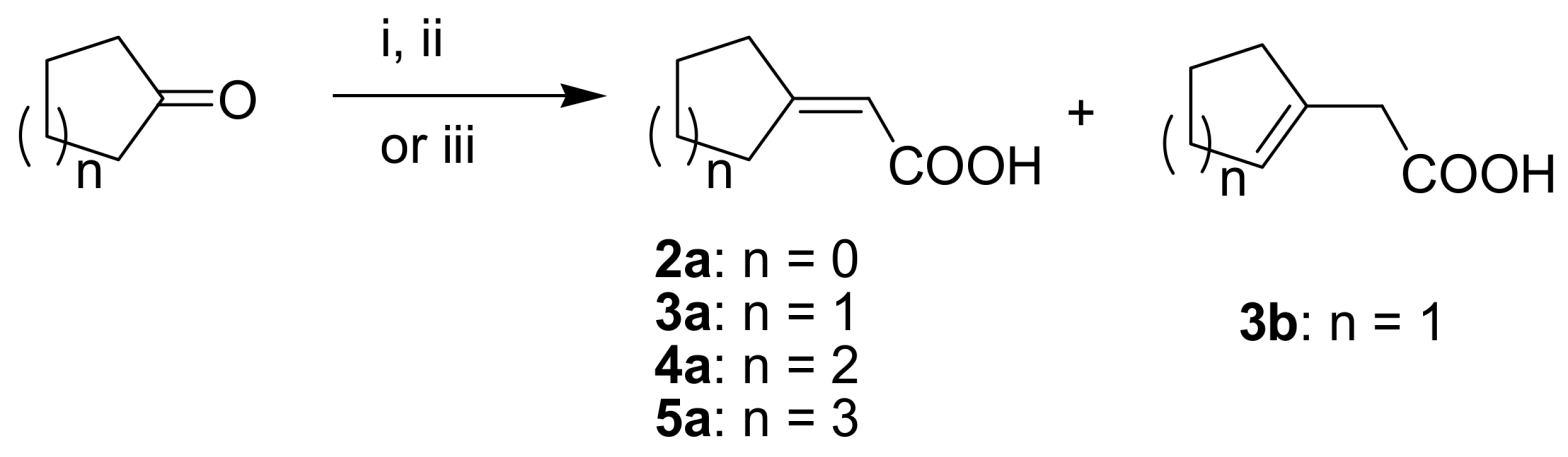
Syntheses of cycloalkylideneacetic acids **2a** to **5a**. (**i**) (EtO)_2_P(O)-CH_2_-COOEt, NaH, Et_2_O, cyclobutanone or cyclopentanone (**ii**) LiOH × H_2_O, THF, H_2_O, MeOH, (**iii**) 1. HP(OMe)_2_, NaH, ClCH_2_COOH, cyclohexanone or cycloheptanone, glyme 2. HCl, H_2_O.

**Scheme 3:**
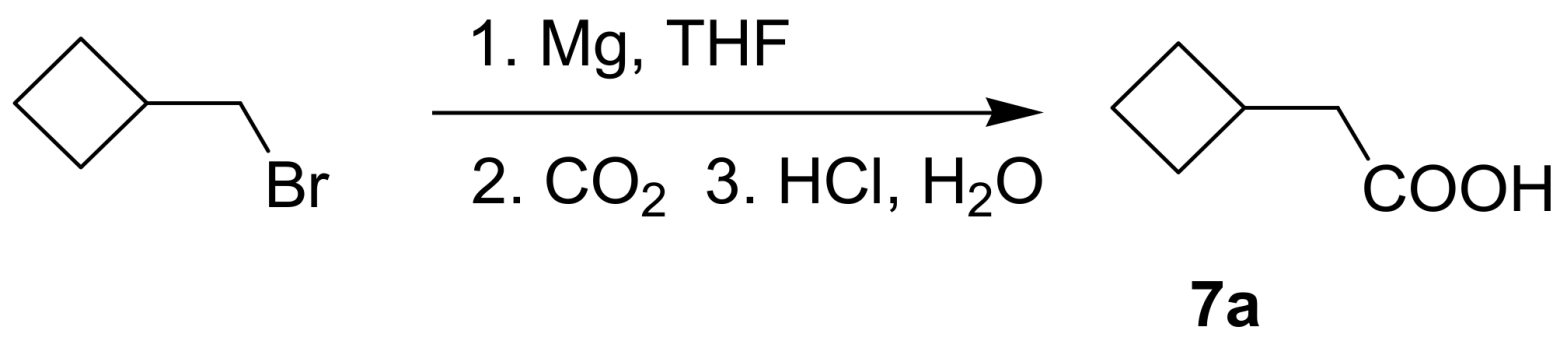
Synthesis of cyclobutylacetic acid (**3a**).

Since shikonin is easier and in bigger amounts acquirable than **1**, we used shikonin as starting material for our synthesis. Shikonin (100% *R*-isomer, seeSupplementary Material) was acylated as usual via Steglich esterification with the corresponding carboxylic acid, dicyclohexylcarbodiimide (DCC) as a coupling reagent and 4-dimethylaminopyridine (DMAP) as catalyst in dichloromethane (Scheme 1) [16,21,22,23,24,25]. As outlined in Scheme 2, cyclohexylideneacetic acid (**4a**) and cycloheptylideneacetic acid (**5a**) resulted from condensation of the corresponding cycloketone with chloroacetic acid mediated by sodium hydride and diethylphosphite [26]. Cyclobutylideneacetic acid (**2a**) and cyclopentylideneacetic acid (**3a**) were synthesized via Horner–Wadsworth–Emmons reaction [27] followed by hydrolysis with lithium hydroxide monohydrate in aqueous THF/MeOH [28]. The C=C double bond of ethyl cyclopentylideneacetate was partially isomerized resulting in a 1:3 mixture of cyclopentylideneacetic acid (**3a**) and 2-cyclopentenylacetic acid (**3b**). **7a** was prepared analogously to 3-ethylpentanoic acid via Grignard reaction using (bromomethyl)cyclobutane and carbon dioxide (Scheme 3) [29].

### 2.2. Cytotoxicity of Investigated Shikonin Derivatives

Since all synthesized derivatives were 100% *R*-isomers, the first question was if the chiral center influences the overall pharmacological activity. Therefore, different samples of **1** with varying enantiomeric ratios were prepared. Melanoma cells were treated with these compounds up to 10.0 µM and for 72 h and the effect was compared to **1** (isolated from roots). We did not detect significant differences in the cytotoxicity indicating that the chiral center has no influence on the activity. In addition, it made no difference if the compound was synthesized (70/30 mixture) or isolated (see Figure S1, Supplementary Material).

**Figure 1:**
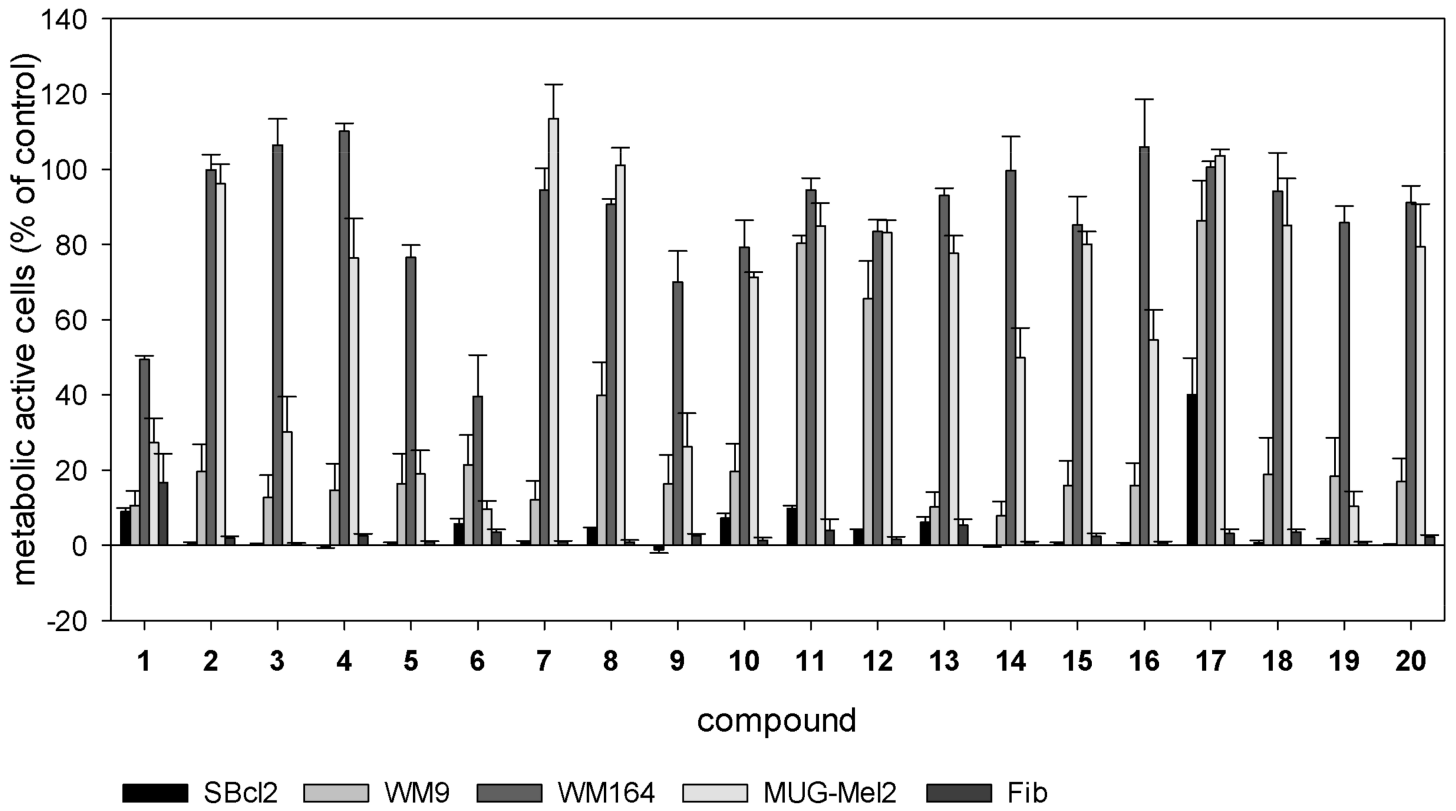
Results of the XTTassay. Melanoma cells (SBcl2, WM9, WM164, and MUG-Mel2) and juvenile skin fibroblasts were treated with 5.0 µM of **2** to **20** for 72 h (mean ± SEM, *n* = 4). Compound **1** serves as reference compound. Results of all tested concentrations can be found in the Supplementary Material.

Subsequently, all derivatives were investigated regarding their cytotoxicity against various melanoma cell lines (SBcl2, WM9, WM164, and MUG-Mel2) and juvenile skin fibroblasts. Cells were treated with different concentrations of **2** to **20** (Figure 1 and, additionally, Figures S2 and S3, Supplementary Material) and their effects compared to **1**. SBcl2 cells represent an early state of cancer progression (cutaneous melanoma, radial growth phase) and are very sensitive to **1** and many other compounds. WM9, WM164, and MUG-Mel2 are derived from melanoma metastases located at the left axillary node (BRAF mutated), right upper arm with stage IV superficial spreading melanoma (BRAF mutated), and from very fast growing metastasis of the left shoulder (NRAS mutated), respectively [30]. Especially WM164 and MUG-Mel2 cells are less susceptible to many cytotoxic compounds and, therefore, difficult to treat. This is also reflected by our results. For example, compounds **2**, **4**, and **7** were considerably less cytotoxic towards WM164 and MUG-Mel2 cells than towards the other cell lines. However, this does not apply to all cycloalkylidenes and cycloalkylacetates. The assumption that an increase of steric bulkiness results in decreased cytotoxic activity [16] does not apply to the cytotoxicity of our cycloalkylideneacetates series **2** to **5** with **5** as the most potent one. This regularity was also not strictly observed for cycloalkylacetates **6** to **9** and cycloalkane carboxylates **11** and **14**. 

Cyclopropylacetate **6** turned out to be significantly more active against the metastatic cell lines WM164 and MUG-Mel2 than **1** (Table 2). This is of special interest because these kinds of cells cause major clinical problems and respond poor to most treatment options. **6** was also more cytotoxic against the melanoma cell lines used than **11**, which was the most active derivative in a previous study [20]. However, it also exhibited cytotoxicity against juvenile skin fibroblasts (IC_50_ = 1.6 ± 0.4 µM). To better assess its cytotoxicity against nontumorigenic cells, **6** was also tested on two other healthy cell types. On the one hand, human embryonic epithelial cells (HEK-293), a well-established nontumorigenic cell line, was used. On the other hand, we used isolated human adult fibroblasts to study the cytotoxicity against another type of fibroblasts. Fibroblasts have been shown to display distinct transcriptional patterns depending on their origin [31]. Compared to juvenile fibroblasts, IC_50_ values of **6** were 3.4 fold higher towards HEK-293 cells (IC_50_ = 5.4 ± 0.7 µM) and 4.0 fold higher against adult fibroblasts (IC_50_ = 6.4 ± 0.7 µM). This shows that the cytotoxicity varies in different nontumorigenic types of cells. Nevertheless, toxicity of chemotherapeutics to healthy cells is a well-known problem in cancer therapy and leads to undesirable side effects in patients. For example, vinblastine, a commonly used chemotherapeutic, exhibited IC_50_ values towards melanoma cells and lung fibroblasts within the same concentration range [8]. Another example is doxorubicin—again a commonly used chemotherapeutic—which showed the same or even a higher cytotoxicity against HEK-293 cells than against breast cancer and leukemia cells [32,33]. However, quinones and derivatives are also members of the PAINS group. PAINS (Pan-Assay Interference Compounds) possess common structural motifs that lead to strong activities in biological assays. PAINS structures occur in natural products (e.g., vitamin K2 and thymoquinone) as well as synthetic drugs. Even some approved chemotherapeutics such as mitoxantrone and doxorubicin contain a PAINS motif. PAINS structures lead, for example, to reactions with nucleophiles such as thiols or amines and cause redox cycling. Quinones including shikonin derivatives possess strong redox activity. Therefore, they can react with nucleophiles, for example, in the side chains of proteins [34]. This, in turn, can lead to adverse side effects. To overcome or reduce these adverse effects, one might be tempted to use smart **6**-loaded targeted nanoparticles. It has been reported that blood vessels of tumors are leaky allowing nanoparticles to penetrate specifically into the tumor tissue. In addition, lymphatic drainage in tumors is poor retaining the accumulated nanoparticles and allowing the drug to be released [35]. Moreover, shikonin-loaded nanoparticles improved the antitumor effects of shikonin in glioma cells in vitro and the particles accumulated in the brain of rats [36]. For melanoma, it has been demonstrated recently that self-assembled nanomicelles of clotrimazole improve drug delivery and apoptosis and, at the same time, inhibit tumor progression [37]. Therefore, we assume that this might be a promising way for further development of **6**. However, development, characterization as well as in vitro and in vivo testing of such nanoparticles goes beyond the scope of the current work. 

Returning to the structure activity relationship of our synthesized compounds, methylation of the cyclopropane moiety of **11** further reduced the activity (see compound **17**). An additional double bond in the carbocyclus, as present in compounds **10**, **15** and **16**, did not enhance cytotoxicity and, compared to the corresponding monocyclic derivative, an additional methylene bridge as presented in the bicyclic derivatives **18** to **20** provides no regularity concerning activity. 

In summary, (*R*)-1-(1,4-dihydro-5,8-dihydroxy-1,4-dioxonaphthalen-2-yl)-4-methylpent-3-enyl cyclopropylacetate (cyclopropylacetylshikonin, **6**) was the most active derivative of this series. The IC_50_ values towards the metastatic cell lines WM164 and MUG-Mel2 were 1.7 fold (WM164, *p* = 0.05) and 2.3 fold (MUG-Mel2, *p* ≤ 0.001) lower than those of **1**, while the effectiveness against the other melanoma cell lines was similar compared to **1** (Table 2).

### 2.3. Pharmacological Effects of Cyclopropylacetylshikonin *(**6**)*

**Figure 2:**
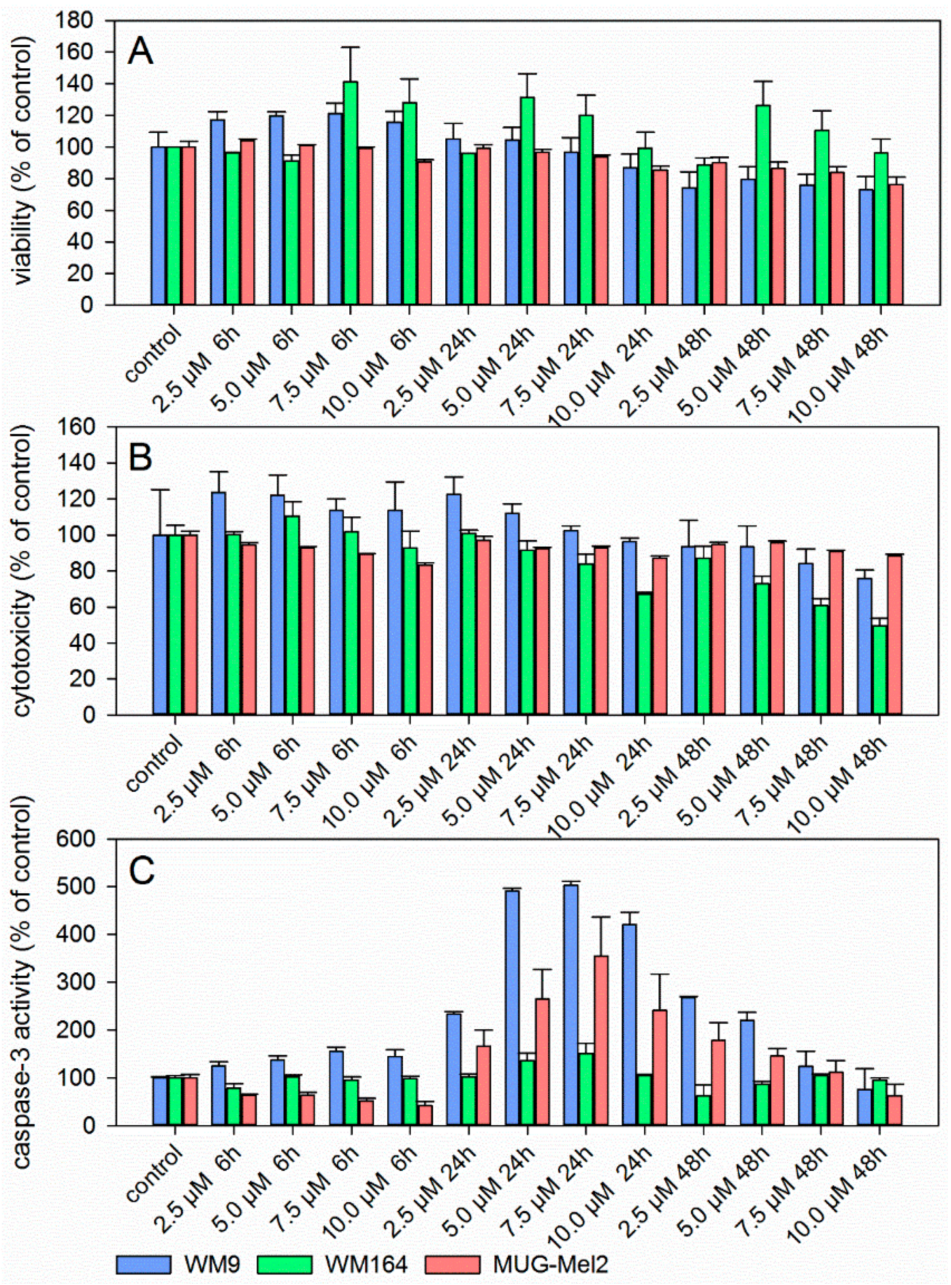
Results of the ApoTox-Glo™Triplex assay showing cell viability (**A**), cytotoxicity (**B**), and caspase 3/7 activity (**C**) in different melanoma cells after 6 h, 24 h, and 48 h treatment with **6** (mean ± SEM, *n* = 6). Staurosporine (10.0 µM) served as positive control for apoptosis induction (caspase activity after 24 h: WM9: 724.8 ± 3.8%; WM164: 898.6 ± 18.4%, and MUG-Mel2: 637.7 ± 64.8% and after 48 h: WM9: 181.1 ± 4.1%, WM164: 226.6 ± 22.5%, and MUG-Mel2: 314.1 ± 41.3%).

**Figure 3:**
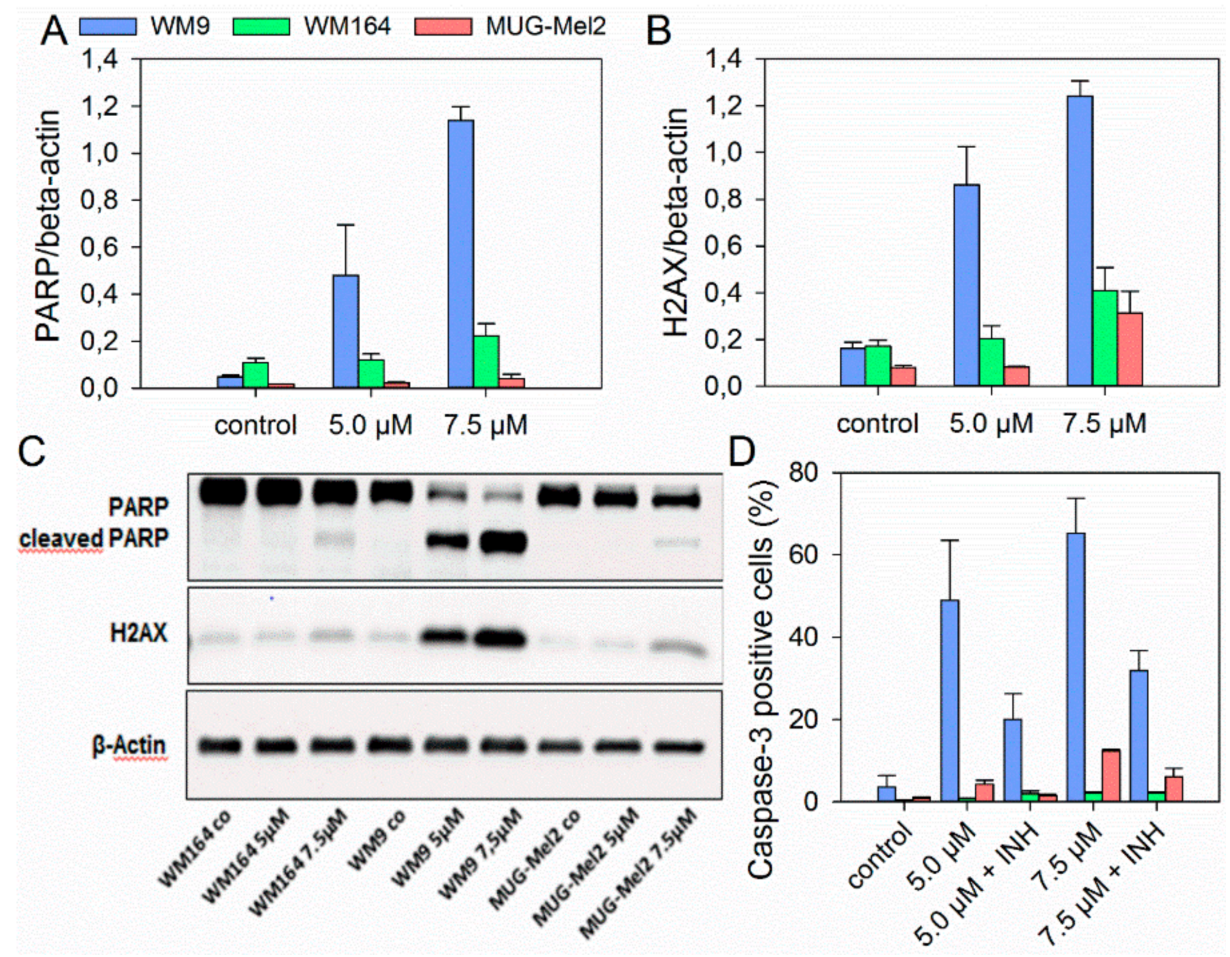
Western blot (**A**–**C**) and flow cytometric experiments (**D**) concerning apoptosis induction in different melanoma cells. (**A**) Densitometric analysis of the apoptotic marker cleaved PARP and (**B**) the DNA damage marker phosphorylated H2AX. The effect of **6** on three different melanoma cell lines was evaluated by immunoblotting under control conditions and in the presence of 5.0 µM and 7.5 µM of **6** for 24 h. Fold change is normalized to controls (mean ± SD, *n* = 3). (**C**) One representative immunoblot out of three is shown. β-Actin was used as loading control. (**D**) Results of cleaved caspase 3 FACS measurements after treatment with **6** (5.0 and 7.5 µM) and 24 h incubation with or without caspase 3 inhibitor (INH), which confirmed the results above (mean ± SEM, *n* = 6).

Since **6** revealed the strongest growth inhibitory activity towards the melanoma cell lines used, its pharmacological effects were investigated in more detail. Figure 2 summarizes the results of the ApoToxGlo™ assay. This assay determines cell viability, compound cytotoxicity, and the activity of caspase 3/7, indicative for apoptosis induction, simultaneously in the same well. Cell viability of WM9 and MUG-Mel2 was slightly reduced after 24 h at 7.5 µM (Figure 2A). However, this change was not statistically significant. WM164 cells showed no reduction of cell viability but a slight, however, again not statistically significant increase. Cytotoxicity was minimally decreased in all cell lines after 48 h (Figure 2B). Caspase 3/7 activity peaked after 24 h with the maximum at 5.0 µM and 7.5 µM (Figure 2C). The strongest activation was found for WM9 cells. In WM164, there was just a marginal increase in caspase 3/7 activity. In previous investigations, we demonstrated that **1** also induced apoptosis caspase 3 dependently after 24 h [8]. The induction of apoptosis by shikonin derivatives was also reported in other types of cancer such as medullary thyroid carcinoma cells [6], human gastric cancer cells, and human breast cancer cells [38,39]. To confirm apoptosis induction, immunoblotting and flow cytometric experiments were performed. Caspases are produced as inactive precursors and cleaved into active enzymes when needed. Thereby, many factors, e.g., the induction of double-stranded DNA breaks, can lead to caspase activation. When DNA double-stranded breaks occur, H2AX is phosphorylated as consequence and, subsequently, induces apoptosis. Activated caspases, in turn, cleave other proteins, for example poly (ADP-ribose) polymerase (PARP) [40]. Therefore, protein expression of cleaved PARP and phosphorylated H2AX was investigated. The levels of both proteins were increased by **6** as shown in Figure 3A–C. Our results indicate that DNA damage caused by **6** might be one reason for apoptosis induction. Also, by using FACS experiments, caspase 3 cleavage was seen in MUG-Mel2 and, particularly, in WM9 cells (Figure 3D), confirming the previous results. Furthermore, the combination of **6** and the caspase inhibitor Z-VAD-FMK led to a declineof caspase 3 activation again confirming the involvement of caspases. 

**Figure 4:**
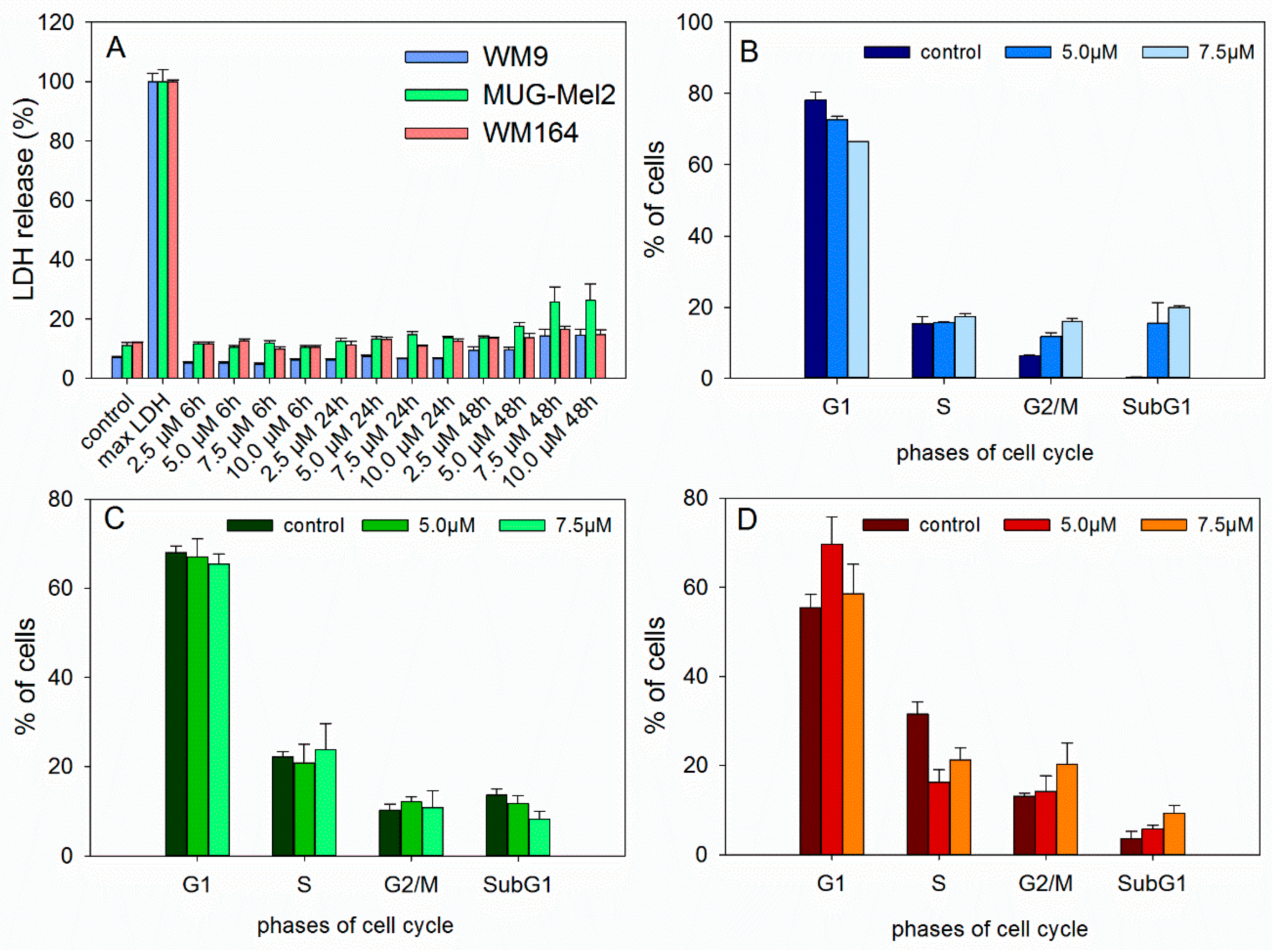
(**A**) Results of lactate dehydrogenase (LDH) assay showing that **6** caused no significant damage to the cell membrane in different melanoma cells (mean ± SEM, *n* = 6). Maximum LDH release control was used to determine maximal LDH release. (**B**–**D**) Treatment of melanoma cells with **6** did not lead to significant changes in the cell cycle distribution (B: WM9, C: MUG-Mel2, and D: WM164 cells, 24 h of incubation, mean ± SEM, *n* = 3).

Since a joint decline of viability and cytotoxicityin the ApoToxGlo™ assay can be a hint of necrosis induction, the CytoTox 96^®^ Non-Radioactive Cytotoxicity Assay (LDH assay) was performed (Figure 4A). The results show that **6** did not significantly damage cell membrane up to 5.0 µM and 48 h incubation. This indicates that necrosis is not involved in the observed cell death. 

Previous investigations have also demonstrated that **1** affected the amount of melanoma cells in the S- (SBcl2 and WM35 cells) or G2/M-phase (WM9 and WM164 cells) [8]. Also shikonin led to a cell cycle arrest in different cancer cell lines and immortalized human keratinocytes [41,42,43,44,45]. Therefore, the effects of **6** on cell cycle distribution were investigated (Figure 4B–D). However, **6** did not lead to a significant cell cycle arrest, suggesting a different mode of action in these cell lines. 

## 3. Material and Methods

### 3.1. Chemicals and Synthesis of Derivatives

#### 3.1.1. Chemicals

Shikonin was obtained from Chengdu Biopurify Phytochemicals Ltd. (Chengdu, China). Reagents and solvents were obtained from commercial suppliers. Solvents were dried and purified using standard techniques.

#### 3.1.2. Synthesis

Compounds were synthesized as described below and in the Supplementary Material. Derivatives with additional chiral centers in the acyl side chain were not separated into diastereomers. Solvents were of p.a. quality, if not stated otherwise. Analytical thin layer chromatography (TLC) was performed using aluminium foil plates coated with silica 60 F_254_ (Merck, Darmstadt, Germany). Shikonin derivatives were detected in VIS. Some intermediates were additionally visualized by UV 254 nm and spraying with molybdatophosphoric acid and subsequent heating. Preparative thin layer chromatography (PTLC) was done on analytical aluminium or plastic foil plates coated with silica 60 F_254_ (Merck, Darmstadt, Germany) and developed until the solvent front reached 8 cm. For column chromatography (CC), silica gel 60 (63–200 µm, Merck, Darmstadt, Germany) was used. Infrared spectra were recorded on a Bruker Alpha Platinum ATR spectrometer (Bruker, Kennewick, WA, USA). ^1^H and ^13^C-NMR spectra were recorded on Varian 400 MHz UnityINOVA spectrometer (400 and 100 MHz, respectively, Varian, Palo Alto, CA, USA) using deuterated chloroform (CDCl_3_) as the solvent and TMS as internal standard. ^1^H- and ^13^C-resonances are indicated as given in the Table 1. Assignments marked with an asterisk are interchangeable. The purity of all synthesized compounds was verified using NMR and analytical HPLC. The purity exceeded 95%. Liquid chromatography coupled with electrospray ionization-tandem mass spectrometry (LC-ESI-MS) analyses were carried out on a Dionex Ultimate 3000 UHPLC (Thermo, San José, CA, USA) coupled with a Thermo LTQ XL linear ion trap mass spectrometer equipped with an H-ESI II probe in the negative mode using 500 nm as acquisition wavelength (source heater temperature, 250 °C, capillary temperature, 200 °C, source voltage, 3.5 kV, capillary voltage, −14 V, sheath gas flow, 50 arbitrary units, auxiliary gas flow, 10 arbitrary units). As stationary phase, a Kinetex C18 column (2.6 µm, 100 × 2.10 mm, Phenomenex, Torrance, FL, USA) was used. The mobile phase consisted of water (A) and acetonitrile (B). The gradient was set as follows 0–45 min: 55–100% B. Flow rate was 0.2 mL/min. Column temperature was set at 30 °C. 

#### 3.1.3. General Procedure for the Acylation of Shikonin

A solution of shikonin in abs. CH_2_Cl_2_ (0.1 mmol/5 mL) was cooled to 0 °C under argon atmosphere and DCC was added. DMAP was added after stirring for 15 min. After further stirring for 15 min, the corresponding acid was added and stirring was continued for 14 h to 5 days with slowly warming up to room temperature. After addition of 1 mL cyclohexane/0.1 mmol shikonin, the mixture was concentrated under reduced pressure at room temperature to approx. 0.5 mL/0.1 mmol shikonin. The mixture was filtered over 3 mm silica and 2 mm celite^®^ (eluent: petroleum ether/CH_2_Cl_2_ = 1:0 to 1:2). Product containing fractions were evaporated and submitted to flash CC and/or repeated PTLC (cyclohexane/CH_2_Cl_2_ mixtures). Due to the rapid decomposition of raw acylshikonin mixtures on evaporation to higher concentrations (c > approx. 0.2 M acylshikonin) and degradation of acylshikonins on prolonged contact with silica, intermediate solutions were not to be concentrated to dryness and all isolation and purification steps had be performed at a good pace.

*(R)-1-(1,4-Dihydro-5,8-dihydroxy-1,4-dioxonaphthalen-2-yl)-4-methylpent-3-enyl 2-cyclobutylideneacetate* (**2**): 0.1 mmol Shikonin, 0.20 mmol DCC, 30 µmol DMAP, and 0.1 mmol 2-cyclobutylideneacetic acid (**2a**); reaction time 5 days; PTLC on silica (developed twice with cyclohexane/CH_2_Cl_2_ = 2:1 and twice with cyclohexane/CH_2_Cl_2_ = 1:2) and PTLC on silica with cyclohexane/CH_2_Cl_2_ = 2:1 (three times developed), **2**, yield: 3%. **2**: R_f_ = 0.31 (silica, cyclohexane/CH_2_Cl_2_ = 1:4); IR (ATR): 2957 (w), 2919 (m), ≈ 2950 (br), 2851 (w), 1716 (m), 1668 (m), 1609 (m), 1568 (m), 1454 (m), 1263 (s), 1202 (s), 1170 (s), 1108 (m), 703 (w) cm^−1^; ^1^H-NMR (CDCl_3_): δ 1.58 (s, 3H, H-6′), 1.69 (s, 3H, H-5′), 2.12 (quint, *J* = 7.9 Hz, 2H, H-3′′), 2.49 (dtm, *J* = 14.9, 7.4 Hz, 1H, H-2′), 2.62 (dtm, *J* = 14.9, 5.5 Hz, 1H, H-2′), 2.88 (t, *J* = 7.6 Hz, 2H, H-2′′, H-4′′), 3.14 (qm, *J* = 7.7 Hz, 2H, H-2′′, H-4′′), 5.14 (tm, *J* = 7.1 Hz, 1H, H-3’), 5.68 (quint, 1H, *J* = 2.1 Hz, H-α), 6.02 (dd, *J* = 7.1, 4.4 Hz, 1H, H-1’), 6.96 (d, *J* = 0.9 Hz, 1H, H-3), 7.18 (s, 2H, H-6, H-7), 12.44 (s, 1H, C5-OH), 12.59 (s, 1H, C8-OH); ^13^C-NMR (CDCl_3_): δ 17.6 (C-3′′), 18.0 (C-6’), 25.8 (C-5’), 32.4 (C-2′′*), 32.9 (C-2’), 34.1 C-4′′*), 69.0 (C-1’), 111.6 (C-4a, C-α), 111.8 (C-8a), 118.1 (C-3’), 131.6 (C-3), 132.5 (C-6, C-7), 135.9 (C-4’), 148.9 (C-2), 165.1 (COO), 166.2 (C-5), 166.8 (C-8), 169.8 (C-1′′), 177.4 (C-1), 179.0 (C-4); MS (ESI^−^) *m*/*z* (%): 819 (16) [2(M − H) + Cl + Na]^−^, 785 (37) [2(M − H) + Na]^−^, 417 and 415 (14) [M − 2H + Cl]^−^, 382.11 (100) [M]^−^; 381.21 (53) [M − H]^−^, 137 (44); [M]^−^ calculated for C_22_H_22_O_6_: 382.1416. 

*(R)-1-(1,4-Dihydro-5,8-dihydroxy-1,4-dioxonaphthalen-2-yl)-4-methylpent-3-enyl 2-cyclopentylideneacetate* (**3**): 0.1 mmol Shikonin, 0.20 mmol DCC, 25 µmol DMAP, and 0.11 mmol 2-cyclopentylideneacetic acid (**3a**); reaction time 16 h; PTLC on silica (developed twice with cyclohexane/CH_2_Cl_2_ = 2:1 and twice with cyclohexane/CH_2_Cl_2_ = 1:2) and PTLC on silica with cyclohexane/CH_2_Cl_2_ = 2:1 (four times developed), **3**, yield: 5%. **3**: R_f_ = 0.37 (silica, CH_2_Cl_2_); IR (ATR): 2959 (w), 2918 (w), ≈2950 (br), 2850 (w), 1717 (m), 1648 (m), 1609 (vs), 1569 (m), 1453 (m), 1230 (m), 1188 (vs), 1114 (s) cm^−1^; ^1^H-NMR (CDCl_3_): 1.58 (s, 3H, H-6’), 1.68 (m, 2H, H-4′′), 1.69 (s, 3H, H-5’), 1.75 (m, 2H, H-3′′), 2.48 (m, 3H, H-2’ and H-2′′), 2.63 (dtm, *J* = 15.3, 5.4 Hz, 1H, H-2’), 2.75 (m, 2H, H-5′′), 5.15 (tm, *J* = 7.2 Hz, 1H, H-3’), 5.90 (quint, 1H, *J* = 1.9 Hz, H-α), 6.02 (dd, *J* = 7.4, 4.7 Hz, 1H, H-1’), 6.97 (d, *J* = 0.8 Hz, 1H, H-3), 7.18 (s, 2H, H-6 and H-7), 12.43 (s, 1H, C5-OH), 12.60 (s, 1H, C8-OH); ^13^C-NMR (CDCl_3_): δ 18.0 (C-6’), 25.5 (C-4′′), 25.8 (C-5’), 26.4 (C-3′′), 32.9, 33.0 (C-2’, C-2′′), 36.2 (C-5′′), 68.7 (C-1’), 110.9 (C-α), 111.7 (C-4a), 111.8 (C-8a), 118.1 (C-3’), 131.6 (C-3), 132.4 (C-7), 133.5 (C-6), 135.8 (C-4’), 149.2 (C-2), 165.5 (COO), 166.1 (C-5), 166.7 (C-8), 171.5 (C-1′′), 177.7 (C-1), 179.1 (C-4); MS (ESI^−^) *m*/*z* (%): 813 (10) [2(M − H)+Na]^−^, 395.30 (100) [M − H]^−^; [M − H]^−^ calculated for C_23_H_24_O_6_: 395.1495.

*(R)-1-(1,4-Dihydro-5,8-dihydroxy-1,4-dioxonaphthalen-2-yl)-4-methylpent-3-enyl 2-cyclohexylideneacetate* (**4**): 0.1 mmol Shikonin, 0.20 mmol DCC, 25 µmol DMAP, and 0.11 mmol 2-cyclohexylideneacetic acid (**4a**); reaction time 16 h; Flash-CC on silica (3 g) with petroleum ether/CH_2_Cl_2_ = 0:1 to 1:0) and PTLC on silica each developed twice with cyclohexane/CH_2_Cl_2_ = 2:1 and twice with cyclohexane/CH_2_Cl_2_ = 1:2); **4**, yield: 13%. **4**: R_f_ = 0.31 (silica, CH_2_Cl_2_); IR (ATR): 2919 (m), ≈2950 (br) (OH), 2851 (w), 1720 (m) (C=O), 1643 (m), 1610 (s), 1570 (m), 1451 (m), 1266 (m), 1233 (m), 1201 (s) (COC), 1147 (s), 777 (w) cm^−1^; ^1^H-NMR (CDCl_3_): 1.58 (s, 3H, H-6’), 1.59-1.70 (m, 6H, H-3′′, H-4′′, H-5′′), 1.69 (s, 3H, H-5’), 1.75 (m, 2H, H-3′′), 2.23 (t, *J* = 6.5 Hz, 2H, H-2′′), 2.48 (dt, *J* = 15.0, 7.3 Hz, 1H, H-2’), 2.62 (dtm, *J* = 15.2, 5.4 Hz, 1H, H-2’), 2.75 (t, *J* = 5.5 Hz, 2H, H-6′′), 5.15 (tm, *J* = 7.2 Hz, 1H, H-3’), 5.70 (s, 1H, H-α), 6.02 (dd, *J* = 7.4, 4.7 Hz, 1H, H-1’), 6.98 (d, *J* = 0.8 Hz, 1H, H-3), 7.18 (s, 2H, H-6 and H-7), 12.44 (s, 1H, C5-OH), 12.60 (s, 1H, C8-OH); ^13^C-NMR (CDCl_3_): δ 18.0 (C-6’), 25.8 (C-5’), 26.2 (C-4′′), 27.8 (C-5′′), 28.6 (C-3′′), 30.0 (C-6′′), 32.9 (C-2’), 38.1 (C-2′′), 68.6 (C-1’), 111.6 (C-4a), 111.9 (C-8a), 112.1 (C-α), 118.0 (C-3’), 131.7 (C-3), 132.4 (C-7), 132.6 (C-6), 135.9 (C-4’), 149.1 (C-2), 165.4 (COO), 166.0 (C-1′′), 166.3 (C-5), 166.8 (C-8), 177.6 (C-1), 179.1 (C-4); MS (ESI^−^) *m*/*z* (%): 841 (11) [2(M − H) + Na]^−^, 410.23 (47) [M]^−^, 409.23 (100) [M − H]^−^; [M]^−^ calculated for C_24_H_26_O_6_: 410.1729. 

*(R)-1-(1,4-Dihydro-5,8-dihydroxy-1,4-dioxonaphthalen-2-yl)-4-methylpent-3-enyl 2-cycloheptylideneacetate* (**5**): 0.1 mmol Shikonin, 0.20 mmol DCC, 25 µmol DMAP, and 0.11 mmol 2-cycloheptylideneacetic acid (**5a**); reaction time 16 h; PTLC on silica (developed twice with cyclohexane/CH_2_Cl_2_ = 2:1 and twice with cyclohexane/CH_2_Cl_2_ = 1:2) and PTLC on silica with cyclohexane/CH_2_Cl_2_ = 2:1 (four times developed), **5**, yield: 10%. **5**: R_f_ = 0.40 (silica, CH_2_Cl_2_); IR (ATR): 2918 (m), ≈2950 (br), 2850 (w), 1717 (m), 1609 (s), 1569 (m), 1560 (m), 1453 (m), 1201 (m), 1136 (s), 779 (w) cm^−1^; ^1^H-NMR (CDCl_3_): 1.52–1.55 (m, 2H, H-4′′, H-5′′), 1.55 (s, 3H, H-6′), 1.62-1.69 (m, 2H, H-3′′, H-6′′), 1.69 (s, 3H, H-5′), 2.42 (m, 2H, H-2′′), 2.49 (dtm, *J* = 14.8, 7.3 Hz, 1H, H-2′), 2.61 (dtm, *J* = 14.9, 5.0 Hz, 1H, H-2′), 2.85 (m, 2H, H-7′′), 5.15 (tm, *J* = 7.4 Hz, 1H, H-3′), 5.77 (s, 1H, H-α), 6.01 (ddd, *J* = 7.2, 4.6, 0.8 Hz, 1H, H-1′), 6.98 (d, *J* = 0.8 Hz, 1H, H-3), 7.18 (s, 2H, H-6, H-7), 12.44 (s, 1H, C5-OH), 12.60 (s, 1H, C8-OH); ^13^C-NMR (CDCl_3_): δ 18.0 (C-6′), 25.8 (C-5′), 26.6 (C-6′′), 27.9 (C-3′′), 29.0 (C-4′′), 29.8 (C-5′′), 32.3 (C-7′′), 33.0 (C-2′), 39.2 (C-2′′), 68.5 (C-1′), 111.6 (C-4a), 111.9 (C-8a), 114.7 (C-α), 118.1 (C-3′), 131.7 (C-3), 132.4 (C-7), 133.5 (C-6), 135.8 (C-4′), 149.2 (C-2), 165.2 (COO), 166.1 (C-5), 166.7 (C-8), 169.2 (C-1′′), 177.7 (C-1), 179.2 (C-4); MS (ESI^−^) *m*/*z* (%): 869 (15) [2(M − H) + Na]^−^, 424.25 (45) [M]^−^, 423.22 (100) [M − H]^−^ 159 (31), 137 (39); [M]^−^ calculated for C_25_H_28_O_6_: 424.1886.

*(R)-1-(1,4-Dihydro-5,8-dihydroxy-1,4-dioxonaphthalen-2-yl)-4-methylpent-3-enyl cyclopropylacetate* (**6**): 0.2 mmol Shikonin, 0.4 mmol DCC, 80 µmol DMAP, and 0.25 mmol cyclopropylacetic acid (**6a**); reaction time 16 h; CC on silica (7 g) with cyclohexane/CH_2_Cl_2_ = 2:1 to 1:4; **6**, yield 26%. **6**: R_f_ = 0.29 (silica, cyclohexane/CH_2_Cl_2_ = 1:4); IR (ATR): 3080 (w), 2972 (w), ≈2950 (br), 2916 (w), 2857 (w), 1742 (s), 1610 (s), 1570 (m), 1454 (m), 1231 (m), 1204 (s), 784 (m) cm^−1^; ^1^H-NMR (CDCl_3_): 0.17–0.24 (m, 2H, H-2′′, H-3′′), 0.56–0.63 (m, 2H, H-2′′, H-3′′), 1.02–1.13 (m, 1H, H-1′′), 1.58 (s, 3H, H-6′), 1.69 (s, 3H, H-5′), 2.23–2.37 (m, 2H, H-α), 2.49 (dtm, *J* = 15.0, 7.5 Hz, 1H, H-2′), 2.63 (dtm, *J* = 15.0, 5.5 Hz, 1H, H-2′), 5.14 (tm, *J* = 7.1 Hz, 1H, H-3′), 6.05 (ddd, *J* = 7.3, 4.5, 1.0 Hz, 1H, H-1′), 7.01 (d, *J* = 1.0 Hz, 1H, H-3), 7.19 (s, 2H, H-6, H-7), 12.43 (s, 1H, C5-OH), 12.59 (s, 1H, C8-OH); ^13^C-NMR (CDCl_3_): δ 4.4, 4.5 (C-2′′, C-3′′), 7.0 (C-1′′), 18.0 (C-6’), 25.8 (C-5’), 32.9 (C-2’), 39.5 (C-α), 69.4 (C-1’), 111.6 (C-4a), 111.8 (C-8a), 117.8 (C-3’), 131.5 (C-3), 132.7 (C-7), 132.8 (C-6), 136.0 (C-4’), 148.4 (C-2), 166.8 (C-5), 167.4 (C-8), 172.0 (COO), 176.8 (C-1), 178.3 (C-4); MS (ESI^−^) *m*/*z* (%):1165 (5) [3M − 3H + Na + K]^−^, 1147 (7) [3M − 2H + K]^−^, 1131 (11) [3M − 2H + Na]^−^, 761 (1) [2(M − H) + Na]^−^, 637 (2) [2M − 3H − RCOOH]^−^, 370.22 (32) [M]^−^, 369.25 (100) [M − H]^−^; [M]^−^ calculated for C_21_H_22_O_6_: 370.1416.

*(R)-1-(1,4-Dihydro-5,8-dihydroxy-1,4-dioxonaphthalen-2-yl)-4-methylpent-3-enyl cyclobutylacetate* (**7**): 50 µmol Shikonin, 0.15 mmol DCC, 30 µmol DMAP, and 53 µmol cyclobutylacetic acid (**7a**); reaction time 16 h; PTLC on silica (developed twice with cyclohexane/CH_2_Cl_2_ = 2:1 and one time with cyclohexane/CH_2_Cl_2_ = 1:2) and an additional PTLC on silica (developed twice with cyclohexane/CH_2_Cl_2_ = 2:1); **7**, yield: 21%. **7**: R_f_ = 0.21 (silica, cyclohexane/CH_2_Cl_2_ = 1:4); IR (ATR): 2959 (w), 2918 (m), ≈2950 (br), 2851 (w), 1737 (m), 1609 (s), 1568 (m), 1453 (m), 1264 (m), 1229 (m), 1202 (s), 1158 (m), 777 (w) cm^−1^; ^1^H-NMR (CDCl_3_): 1.58 (s, 3H, H-6′), 1.69 (s, 3H, H-5′), 1.70–1.78 (m, 2H, trans H-2′′, trans H-4′′), 1.81–1.97 (m, 2H, H-3′′), 2.09–2.20 (m, 2H, cis H-2′′, cis H-4′′), 2.46 (dtm, *J* = 15.0, 7.5 Hz, 1H, H-2′), 2.49 (d, *J* = 7.5 Hz, 2H, H-α), 2.60 (dtm, *J* = 14.7, 5.9 Hz, 1H, H-2′), 2.71 (quint, *J* = 7.8 Hz, 1H, H-1′′), 5.11 (tm, *J* = 7.3 Hz, 1H, H-3’), 6.00 (ddd, *J* = 7.7, 4.2, 0.9 Hz, 1H, H-1’), 6.96 (d, *J* = 1.0 Hz, 1H, H-3), 7.18 (s, 2H, H-6, H-7), 12.42 (s, 1H, C5-OH), 12.57 (s, 1H, C8-OH); ^13^C-NMR (CDCl_3_): δ 18.0 (C-6’), 18.5 (C-3′′), 25.8 (C-5’), 28.2, 28.3 (C-2′′, C-4′′), 32.2 (C-1′′), 32.9 (C-2’), 41.2 (C-α), 69.2 (C-1’), 111.6 (C-4a), 111.8 (C-8a), 117.8 (C-3’), 131.4 (C-3), 132.7 (C-7), 132.9 (C-6), 136.0 (C-4’), 148.5 (C-2), 166.9 (C-5), 167.4 (C-8), 171.5 (COO), 176.8 (C-1), 178.3 (C-4); MS (ESI^−^) *m*/*z* (%): 789.37 (8) [2(M − H) + Na]^−^, 384.21 [M]^−^ (52), 383.26 (100) [M − H]^−^; [M]^−^ calculated for C_22_H_24_O_6_: 384.1573.

*(R)-1-(1,4-Dihydro-5,8-dihydroxy-1,4-dioxonaphthalen-2-yl)-4-methylpent-3-enyl cyclopentylacetate* (**8**): 0.1 mmol Shikonin, 0.15 mmol DCC, 25 µmol DMAP, and 0.1 mmol cyclopentylacetic acid (**8a**); reaction time 17 h; CC on silica (7 g) with cyclohexane/CH_2_Cl_2_ = 2:5 and PTLC on silica with cyclohexane/CH_2_Cl_2_ = 2:5 (three times developed); **8**, yield 10%. **8**: R_f_ = 0.26 (silica, cyclohexane/CH_2_Cl_2_ = 1:4); IR (ATR): 2951 (m, br), 2866 (w), 1738 (s), 1608 (s), 1569 (m), 1452 (m), 1230 (s), 1200 (s), 1111 (s), 777 (m) cm^−1^; ^1^H-NMR (CDCl_3_): 1.12–1.23 (m, 2H, H-2′′, H-5′′), 1.58 (s, 3H, H-6′), 1.52–1.68 (m, 4H, H-3′′, H-4′′), 1.69 (s, 3H, H-5′), 1.78–1.88 (m, 2H, H-2′′, H-5′′), 2.25 (sept, *J* = 7.7 Hz, 1H, H-1′′), 2.37–2.43 (m, 2H, H-α), 2.47 (dtm, *J* = 15.1, 7.5 Hz, 1H, H-2′), 2.61 (dtm, *J* = 15.0, 5.7 Hz, 1H, H-2′), 5.13 (tm, *J* = 7.3 Hz, 1H, H-3′), 6.03 (ddd, *J* = 7.3, 4.4, 0.8 Hz, 1H, H-1′), 6.99 (d, *J* = 1.0 Hz, 1H, H-3), 7.19 (s, 2H, H-6, H-7), 12.43 (s, 1H, C5-OH), 12.59 (s, 1H, C8-OH); ^13^C-NMR (CDCl_3_): δ 18.0 (C-6′), 25.0 (C-3′′, C-4′′), 25.8 (C-5’), 32.4, 32.5 (C-2′′, C-5′′), 33.0 (C-2′), 36.5 (C-1′′), 40.4 (C-α), 69.2 (C-1′), 111.6 (C-4a), 111.8 (C-8a), 117.9 (C-3′), 131.5 (C-3), 132.7 (C-7), 132.8 (C-6), 136.0 (C-4′), 148.5 (C-2), 166.8 (C-5), 167.4 (C-8), 172.1 (COO), 176.8 (C-1), 178.3 (C-4); MS (ESI^−^) *m*/*z* (%): 1231 (9) [3M − 2H + K]^−^, 1215 (8) [3M − 2H + Na]^−^, 398.23 (34) [M]^−^, 397.30 (100) [M − H]^−^; [M]^−^ calculated for C_23_H_26_O_6_: 398.1729.

*(R)-1-(1,4-Dihydro-5,8-dihydroxy-1,4-dioxonaphthalen-2-yl)-4-methylpent-3-enyl cyclohexylacetate* (**9**): 50 µmol Shikonin, 0.15 mmol DCC, 30 µmol DMAP, and 53 µmol cyclohexylacetic acid (**9a**); reaction time 19 h; flash CC on silica (8 g) with cyclohexane/CH_2_Cl_2_ = 2:1 to cyclohexane/CH_2_Cl_2_ = 0:1) and an additional PTLC on silica (developed three times with cyclohexane/CH_2_Cl_2_ = 2:1 and one time with cyclohexane/CH_2_Cl_2_ = 1:2); **9**, yield: 36%. **9**: R_f_ = 0.21 (silica, cyclohexane/CH_2_Cl_2_ = 1:4); IR (ATR): 2920 (s), ≈2950 (br), 2850 (m), 1738 (s), 1610 (s), 1570 (m), 1450 (s), 1230 (s), 1204 (s), 1160 (m), 1111 (m), 782 (w) cm^−1^; ^1^H-NMR (CDCl_3_): 0.92–1.05 (m, 2H, H-2′′ax, H-6′′ax), 1.09–1.22 (m, 1H, H-4′′ax), 1.21-1.33 (m, 2H, H-3′′ax, H-5′′ax), 1.59 (s, 3H, H-6′), 1.62-1.76 (m, 5H, H-2′′eq, H-3′′eq, H-4′′eq, H-5′′eq, H-6′′eq), 1.76–1.86 (m, 1H, H-1′′), 2.24 (dd, 12.4, 4.7 Hz, 1H, H-α), 2.29 (dd, 12.4, 5.0 Hz, 1H, H-α), 2.47 (dtm, *J* = 14.8, 7.5 Hz, 1H, H-2′), 2.61 (dtm, *J* = 15.1, 5.7 Hz, 1H, H-2′), 5.12 (tm, *J* = 7.2 Hz, 1H, H-3′), 6.04 (ddd, *J* = 7.3, 4.3, 0.8 Hz, 1H, H-1′), 6.99 (d, *J* = 0.8 Hz, 1H, H-3), 7.18 (s, 2H, H-6, H-7), 12.42 (s, 1H, C5-OH), 12.58 (s, 1H, C8-OH); ^13^C-NMR (CDCl_3_): δ 18.0 (C-6′), 25.7 (C-5′), 2 × 26.0, 26.1 (C-3′′, C-4′′, C-5′′), 3 × 33.0 (C-2′, C-2′′, C-6′′), 35.0 (C-1′′), 42.1 (C-α), 69.2 (C-1′), 111.6 (C-4a), 111.8 (C-8a), 117.9 (C-3′), 131.5 (C-3), 132.7 (C-7), 132.8 (C-6), 136.0 (C-4′), 148.5 (C-2), 166.9 (C-5), 167.4 (C-8), 171.8 (COO), 176.8 (C-1), 178.3 (C-4); MS (ESI^−^) *m*/*z* (%): 845 (12) [2(M − H)+Na]^−^, 412.29 (75) [M]^−^, 411.12 (100) [M − H]^−^; [M]^−^ calculated for C_24_H_28_O_6_: 412.1886.

*(R)-1-(1,4-Dihydro-5,8-dihydroxy-1,4-dioxonaphthalen-2-yl)-4-methylpent-3-enyl 1-cyclohexen-1-ylacetate* (**10**): 50 µmol Shikonin, 0.12 mmol DCC, 15 µmol DMAP, and 53 µmol 1-cyclohexen-1-ylacetic acid (**11a**); reaction time 13 h; PTLC on silica with cyclohexane/CH_2_Cl_2_ = 1:1 (three times developed); **10**, yield 5%. **10**: R_f_ = 0.48 (silica, cyclohexane/CH_2_Cl_2_ = 1:4); IR (ATR): 2926 (m), 2857 (w), 2837 (w), 1738 (m), 1608 (s), 1569 (m), 1436 (m), 1409 (m), 1335 (m), 1229 (s), 1202 (s), 1147 (m), 1112 (m), 777 (m) cm^−1^; ^1^H-NMR (CDCl_3_): 1.51–1.68 (m, 4H, H-4′′, H-5′′), 1.55 (s, 3H, H-6′), 1.69 (s, 3H, H-5′), 1.97–2.03 (m, 2H, H-6′′), 2.03–2.09 (m, 2H, H-3′′), 2.47 (dtm, *J* = 15.1, 7.6 Hz, 1H, H-2′), 2.62 (dtm, *J* = 15.0, 5.7 Hz, 1H, H-2′), 3.01 (s, 2H, H-α), 5.13 (tm, *J* = 7.2 Hz, 1H, H-3′), 5.62 (s br, 1H, H-2′′), 6.01 (ddd, *J* = 6.5, 3.5, 0.9 Hz, 1H, H-1’), 6.97 (d, *J* = 1.0 Hz, 1H, H-3), 7.18 (s, 2H, H-6, H-7), 12.42 (s, 1H, C5-OH), 12.57 (s, 1H, C8-OH); ^13^C-NMR (CDCl_3_): δ 18.0 (C-6’), 21.9 (C-5′′), 22.7 (C-4′′), 25.3 (C-3′′), 25.8 (C-5’), 28.6 (C-6’), 32.9 (C-2’), 43.6 (C-α), 69.5 (C-1’), 111.6 (C-4a), 111.9 (C-8a), 117.9 (C-3’), 126.5 (C-2′′), 130.6 (C-1′′), 131.4 (C-3), 132.7 (C-7), 132.9 (C-6), 136.0 (C-4’), 148.4 (C-2), 167.0 (C-5), 167.5 (C-8), 170.7 (COO), 176.7 (C-1), 178.2 (C-4); MS (ESI^−^) *m*/*z* (%): 841 (5) [2(M − H) + Na]^−^, 410.23 (35) [M]^−^, 409.33 (100) [M − H]^−^; [M]^−^ calculated for C_24_H_26_O_6_: 410.1729.

*(R)-1-(1,4-Dihydro-5,8-dihydroxy-1,4-dioxonaphthalen-2-yl)-4-methylpent-3-enyl cyclopropanecarboxylate* (**11**): 0.1 mmol Shikonin, 0.15 mmol DCC, 25 µmol DMAP, and 0.1 mmol cyclopropanecarboxylic acid (**11a**); reaction time 22 h; CC on silica (7 g) with cyclohexane/CH_2_Cl_2_ = 1:1 to 1:4; **11**, yield 20%. **11**: R_f_ = 0.28 (silica, cyclohexane/CH_2_Cl_2_ = 1:4); NMR data fit with literature [20].

*(R)-1-(1,4-Dihydro-5,8-dihydroxy-1,4-dioxonaphthalen-2-yl)-4-methylpent-3-enyl cyclobutanecarboxylate* (**12**): 0.1 mmol Shikonin, 0.15 mmol DCC, 25 µmol DMAP, and 0.1 mmol cyclobutane carboxylic acid (**12a**); reaction time 22 h; double PTLC on silica with cyclohexane/CH_2_Cl_2_ = 1:4; **12**, yield 40%. **12**: R_f_ = 0.35 (silica, cyclohexane/CH_2_Cl_2_ = 1:4); IR (ATR): 2980 (w), ≈2950 (vbr), 2947 (m), 2866 (w), 1736 (s), 1611 (vs), 1570 (m), 1455 (s), 1204 (s), 1162 (s), 787 (w) cm^−1^; ^1^H-NMR (CDCl_3_): 1.58 (s, 3H, H-6′), 1.69 (s, 3H, H-5′), 1.87–2.07 (m, 2H, H-3′′), 2.19–2.36 (m, 4H, H-2′′, H-4′′), 2.47 (dtm, *J* = 14.7, 7.7 Hz, 1H, H-2′), 2.61 (dtm, *J* = 14.9, 5.7 Hz, 1H, H-2′), 3.22 (quintd, *J* = 8.5, 0.7 Hz, 1H, H-1′′), 5.12 (tm, *J* = 7.3 Hz, 1H, H-3′), 6.02 (ddd, *J* = 7.4, 4.5, 0.9 Hz, 1H, H-1′), 6.96 (d, *J* = 1.0 Hz, 1H, H-3), 7.18 (s, 2H, H-6, H-7), 12.43 (s, 1H, C5-OH), 12.59 (s, 1H, C8-OH); ^13^C-NMR (CDCl_3_): δ 17.9 (C-6′), 18.4 (C-3′′), 25.1, 25.2 (C-2′′, C-4′′), 25.8 (C-5’), 32.9 (C-2’), 38.0 (C-1′′), 69.0 (C-1’), 111.6 (C-4a), 111.8 (C-8a), 117.8 (C-3’), 131.4 (C-3), 132.6 (C-7), 132.8 (C-6), 136.0 (C-4’), 148.5 (C-2), 166.7 (C-5), 167.2 (C-8), 174.1 (COO), 176.9 (C-1), 178.4 (C-4); MS (ESI^−^) *m*/*z* (%): 1165 (3) [3M − 3H + Na + K]^−^, 1147 (6) [3M − 2H + K]^−^, 1231 (8) [3M − 2H + Na]^−^, 370.11 (42) [M]^−^, 369.17 (100) [M − H]^−^; [M]^−^ calculated for C_21_H_22_O_6_: 370.1416.

*(R)-1-(1,4-Dihydro-5,8-dihydroxy-1,4-dioxonaphthalen-2-yl)-4-methylpent-3-enyl cyclopentanecarboxylate* (**13**): 0.1 mmol Shikonin, 0.15 mmol DCC, 25 µmol DMAP, and 0.1 mmol cyclopentanecarboxylic acid (**13a**); reaction time 17 h; double PTLC on silica with cyclohexane/CH_2_Cl_2_ = 1:4; **13**, yield 41%. **13**: R_f_ = 0.32 (silica, cyclohexane/CH_2_Cl_2_ = 1:4); IR (ATR): 3050 (w), 2954 (m), 2919 (w), 2870 (w), 1737 (s), 1608 (s), 1570 (m), 1452 (m), 1205 (s), 1141 (s), 767 (m) cm^−1^; ^1^H-NMR (CDCl_3_): 1.58 (s, 3H, H-6′), 1.57–1.66 (m, 2H, H-3′′, H-4′′), 1.66–1.75 (m, 2H, H-3′′, H-4′′), 1.69 (s, 3H, H-5′), 1.75–1.87 (m, 2H, H-2′′, H-5′′), 1.87–1.99 (m, 2H, H-2′′, H-5′′), 2.47 (dtm, *J* = 14.7, 7.5 Hz, 1H, H-2′), 2.62 (dtm, *J* = 14.8, 5.7 Hz, 1H, H-2′), 2.82 (tt, *J* = 7.3, 8.4 Hz, 1H, H-1′′), 5.12 (tm, *J* = 7.3 Hz, 1H, H-3′), 6.02 (ddd, *J* = 7.3, 4.4, 0.8 Hz, 1H, H-1′), 6.98 (d, *J* = 0.9 Hz, 1H, H-3), 7.19 (s, 2H, H-6, H-7), 12.43 (s, 1H, C5-OH), 12.59 (s, 1H, C8-OH); ^13^C-NMR (CDCl_3_): δ 18.0 (C-6′), 2 × 25.8 (C-5′, C-3′′, C-4′′), 29.9, 30.0 (C-2′′, C-5′′), 33.0 (C-2′), 43.8 (C-1′′), 69.0 (C-1′), 111.6 (C-4a), 111.8 (C-8a), 117.8 (C-3′), 131.4 (C-3), 132.6 (C-7), 132.8 (C-6), 135.9 (C-4′), 148.7 (C-2), 166.7 (C-5), 167.2 (C-8), 175.4 (COO), 176.9 (C-1), 178.5 (C-4); MS (ESI^−^) *m*/*z* (%): 1189 (7) [3M − 2H + K]^−^, 384.13 (62) [M^−^], 383.28 (100) [M − H]^−^; [M]^−^ calculated for C_22_H_24_O_6_: 384.1573.

*(R)-1-(1,4-Dihydro-5,8-dihydroxy-1,4-dioxonaphthalen-2-yl)-4-methylpent-3-enyl cyclohexanecarboxylate* (**14**): 50 µmol Shikonin, 0.12 mmol DCC, 15 µmol DMAP, and 53 µmol cyclohexanecarboxylic acid (**14a**); reaction time 16 h; PTLC on silica with cyclohexane/CH_2_Cl_2_ = 1:4 (twice developed) and an additional PTLC on silica (developed twice with cyclohexane/CH_2_Cl_2_ = 1:1 and twice with cyclohexane/CH_2_Cl_2_ = 1:2); **14**, yield 10%. **14**: R_f_ = 0.24 (silica, cyclohexane/CH_2_Cl_2_ = 1:4); IR (ATR): 2927 (m), ≈2950 (br), 2852 (w), 1736 (s), 1609 (s), 1570 (m), 1451 (m), 1230 (m), 1203 (m), 1162 (m), 1159 (s) cm^−1^; ^1^H-NMR (CDCl_3_): 1.18–1.38 (m, 3H, H-3′′ax, H-4′′ax, H-5′′ax), 1.40-1.54 (m, 2H, H-2′′ax, H-6′′ax), 1.58 (s, 3H, H-6′), 1.62–1.69 (m, 1H, H-4′′eq), 1.69 (s, 3H, H-5′), 1.74–1.81 (m, 2H, H-3′′eq, H-5′′eq), 1.94 (dm, *J* = 11.9 Hz, 2H, H-2′′eq, H-6′′eq), 2.38 (tt, *J* = 11.2, 3.7 Hz, 1H, H-1′′), 2.47 (dtm, *J* = 15.0, 7.3 Hz, 1H, H-2′), 2.61 (dtm, *J* = 14.8, 5.8 Hz, 1H, H-2′), 5.12 (tm, *J* = 7.3 Hz, 1H, H-3′), 6.03 (ddd, *J* = 7.3, 4.5, 0.9 Hz, 1H, H-1′), 6.97 (d, *J* = 1.0 Hz, 1H, H-3), 7.18 (s, 2H, H-6, H-7), 12.43 (s, 1H, C5-OH), 12.58 (s, 1H, C8-OH); ^13^C-NMR (CDCl_3_): δ 18.0 (C-6′), 2 × 25.4 (C-3′′, C-5′′), 25.7 (C-4′′), 26.9 (C-5′), 28.9, 29.0 (C-2′′, C-6′′), 33.0 (C-2’), 43.3 (C-1′′), 68.9 (C-1’), 111.6 (C-4a), 111.9 (C-8a), 117.8 (C-3’), 131.4 (C-3), 132.6 (C-7), 132.8 (C-6), 135.9 (C-4’), 148.7 (C-2), 166.7 (C-5), 167.3 (C-8), 174.7 (COO), 177.0 (C-1), 178.5 (C-4); MS (ESI^−^) *m*/*z* (%): 817 (5) [2(M − H) + Na]^−^, 398.16 (51) [M]^−^, 397.28 (100) [M − H]^−^; [M]^−^ calculated for C_23_H_26_O_6_: 398.1729.

*(R)-1-(1,4-Dihydro-5,8-dihydroxy-1,4-dioxonaphthalen-2-yl)-4-methylpent-3-enyl cyclohex-1-enecarboxylate* (**15**): 0.1 mmol Shikonin, 0.17 mmol DCC, 25 µmol DMAP, and 0.1 mmol cyclohex-1-enecarboxylic acid (**15a**); reaction time 16 h; PTLC on silica (developed twice with cyclohexane/CH_2_Cl_2_ = 2:1 and one time with cyclohexane/CH_2_Cl_2_ = 1:2) and an additional PTLC on silica (developed twice with cyclohexane / CH_2_Cl_2_ = 2:1); **15**, yield 13%. **15**: R_f_ = 0.37 (silica, cyclohexane / CH_2_Cl_2_ = 1:4); IR (ATR): 2917 (m), ≈2950 (br), 2890 (w), 1712 (m), 1609 (s), 1569 (m), 1452 (m), 1229 (s), 1203 (s), 1077 (m) cm^−1^; ^1^H-NMR (CDCl_3_): 1.57 (s, 3H, H-6′), 1.60–1.69 (m, 4H, H-4′′, H-5′′), 1.69 (s, 3H, H-5′), 2.20–2.32 (m, 4H, H-3′, H-6′′), 2.52 (dtm, *J* = 15.0, 7.2 Hz, 1H, H-2′), 2.64 (dtm, *J* = 15.1, 5.4 Hz, 1H, H-2′), 5.14 (tm, *J* = 7.2 Hz, 1H, H-3′), 6.03 (ddd, *J* = 7.1, 4.6, 0.8 Hz, 1H, H-1′), 6.96 (d, *J* = 1.0 Hz, 1H, H-3), 7.09 (m, 1H, H-2′′), 7.19 (s, 2H, H-6, H-7), 12.43 (s, 1H, C5-OH), 12.59 (s, 1H, C8-OH); ^13^C-NMR (CDCl_3_): δ 18.0 (C-6′), 21.4, 22.0 (C-5′′, C-4′′), 24.1 (C-3′′*), 25.8 (C-5′), 25.9 (C-6′′*), 32.9 (C-2′), 69.2 (C-1′), 111.6 (C-4a), 111.9 (C-8a), 117.8 (C-3′), 129.9 (C-1′′), 131.5 (C-3), 132.5 (C-7), 132.6 (C-6), 135.9 (C-4′), 141.1 (C-2′′), 148.9 (C-2), 166.1 (COO), 166.3 (C-5), 166.9 (C-8), 177.4 (C-1), 178.9 (C-4); MS (ESI^−^) *m*/*z* (%): 813 (11) [2(M − H) + Na]^−^, 396.24 (44) [M]^−^, 395.29 (100) [M − H]^−^; [M]^−^ calculated for C_23_H_24_O_6_: 396.1573.

*(R)-1-(1,4-Dihydro-5,8-dihydroxy-1,4-dioxonaphthalen-2-yl)-4-methylpent-3-enyl cyclohex-3-enecarboxylate* (**16**): 0.1 mmol Shikonin, 0.20 mmol DCC, 30 µmol DMAP, and 0.1 mmol cyclohex-3-enecarboxylic acid (**16a**); reaction time 16 h; two subsequent PTLC on silica each one developed twice with cyclohexane/CH_2_Cl_2_ = 2:1 and twice with cyclohexane/CH_2_Cl_2_ = 1:2); **16**, yield: 29%. **16**: 1:1 mixture of diastereomers; R_f_ = 0.28 (silica, CH_2_Cl_2_); IR (ATR): 3025 (vw), 2917 (m), ≈2950 (br), 2849 (w), 1735 (s), 1608 (s), 1568 (m), 1452 (m), 1220 (s), 1201 (s), 1155 (s) cm^−1^; ^1^H-NMR (CDCl_3_): 1.59 (s, 3H, H-6′), 1.70 (s, 3H, H-5′), 1.68–1.81 (m, 1H, H-6′′), 2.00–2.08 (m, 1H, H-6′′), 2.10–2.17 (m, 2H, H-5′′), 2.26–2.32 (m, 2H, H-2′′), 2.49 (dtm, *J* = 15.0, 7.3 Hz, 1H, H-2′), 2.59–2.71 (m, 2H, H-2′ and H-1′′), 5.13 (tm, *J* = 6.6 Hz, 1H, H-3′), 5.66–5.76 (m, 2H, H-3′′, H-4′′), 6.05 (m, 1H, H-1′), 6.98 (2 d, 1H, *J* ≈1 Hz, H-3), 7.19 (s, 2H, H-6, H-7), 12.42 (s, 1H, C5-OH), 12.58 (2 s, 1H, C8-OH); ^13^C-NMR (CDCl_3_): δ 18.0 (C-6′), 24.2 and 24.3 (C-5′′), 24.9 and 25.0 (C-6′′), 25.8 (C-5′), 27.3, 27.4 (C-2′′), 33.0 (C-2′), 2 × 39.3 (C-1′′), 69.1, 69.2 (C-1′), 111.6 (C-4a), 111.8 (C-8a), 117.7, 117.8 (C-3′), 124.9, 125.0 (C-3′′), 2 × 126.8 (C-4′′), 131.3 (C-3), 2 × 132.7 (C-7), 2 × 132.9 (C-6), 136.0 (C-4′), 2 × 148.5 (C-2), 2 × 167.0 (C-5), 167.5, 167.6 (C-8), 174.4, 174.5 (COO), 176.6, 176.7 (C-1), 178.1, 178.2 (C-4); MS (ESI^−^) *m*/*z* (%): 813.41 (7) [2(M − H) + Na]^−^, 395.16 [M − H]^−^; [M − H]^−^ calculated for C_23_H_24_O_6_: 395.1495.

*(R)-1-(1,4-Dihydro-5,8-dihydroxy-1,4-dioxonaphthalen-2-yl)-4-methylpent-3-enyl trans 2-methylcyclopropanecarboxylate* (**17**): 0.1 mmol Shikonin, 0.2 mmol DCC, 82 µmol DMAP, and 0.11 mmol 2-methyl-cyclopropane carboxylic acid (cis/trans mixture ca. 1:3,5) (**17a**); reaction time 15 h; CC on silica (7 g) with cyclohexane/CH_2_Cl_2_ = 2:1 to 0:1 and PTLC on silica with cyclohexane/CH_2_Cl_2_ = 2:1 (five times developed); two diastereomers of **17**, yield 3%. **17**: R_f_ = 0.26 (silica, CH_2_Cl_2_); IR (ATR): 2960 (w), 2919 (w), ≈2950 (br), 2856 (w), 1732 (s), 1609 (s), 1569 (m), 1559 (m), 1454 (m), 1406 (m), 1205 (m), 1177 (m), 1159 (s), 781 (m) cm^−1^; ^1^H-NMR (CDCl_3_): 0.70–0.79 (m, 2H, 2 × H-3′′), 1.12–1.20 (m, 7H, 2 × cyclopropyl-CH_3_, 1 × H-3′′), 1.20–1.26 (m, 1H, H-3′′), 1.36–1.48 (m, 4H, 2 H-1′′, 2 × H-2′′), 1.58 (s, 6H, H-6′), 1.70 (s, 6H, H-5′), 2.42-2.53 (m, 2H, 2 × H-2′), 2.57–2.66 (m, 2H, 2 × H-2′), 5.08–5.16 (m, 2H, 2 × H-3′), 6.00 (tm, *J* = 5.9 Hz, 2H, H-1′), 6.99 (d, *J* = 0.9 Hz, 1H, H-3), 7.00 (d, *J* = 0.9 Hz, 1H, H-3), 7.18 (s, 4H, H-6, H-7), 12.44 (2s, 2H, C5-OH), 12.58 (s, 2H, C8-OH); ^13^C-NMR (CDCl_3_): δ 2 × 17.2 (C-3′′), 2 × 17.7 (C-2′′), 3 × 17.9 (cyclopropyl-CH_3_, C-6′), 2 × 21.2 (C-1′′), 25.8 (C-5′), 32.9 (C-2′), 69.2, 69.3 (C-1′), 111.6 (C-4a), 111.8 (C-8a), 117.7 (C-3′), 2 × 131.6 (C-3), 132.6 (C-7), 132.7 (C-6), 136.0 (C-4′), 148.6 (C-2), 166.5 (C-5), 167.0 (C-8), 2 × 173.3 (COO), 2 × 177.2 (C-1), 178.7, 178.8 (C-4); MS (ESI^−^) *m*/*z* (%): 761.32 (12) [2(M − H)+Na]^−^, 370.14 (46) [M]^−^, 369.26 (100) [M − H]^−^; [M]^−^ calculated for C_21_H_22_O_6_: 370.1416. 

*Exo-(R)-1-(1,4-dihydro-5,8-dihydroxy-1,4-dioxonaphthalen-3-yl)-4-methylpent-3-enyl bicyclo[2 .2.1]hept-5-ene-2-carboxylate* (**18**) and *endo-(R)-1-(1,4-dihydro-5,8-dihydroxy-1,4-dioxonaphthalen-3-yl)-4-methylpent-3-enyl bicyclo[2.2.1]hept-5-ene-2-carboxylate* (**19**): 0.1 mmol Shikonin, 0.20 mmol DCC, 25 µmol DMAP, and 0.11 mmol bicyclo[2.2.1]hept-5-ene-2-carboxylic acid (Exo (**18a**)/Endo (**19a**)/ = 1.0:4.1); reaction time 16 h; PTLC on silica (developed three times with cyclohexane/CH_2_Cl_2_ = 2:1 and one time with cyclohexane/CH_2_Cl_2_ = 1:2) and PTLC on silica (developed twice with cyclohexane/CH_2_Cl_2_ = 2:1 and three times with cyclohexane/CH_2_Cl_2_ = 1:2), yield: **18**, 6% and **19** 10%. **18**: 1:1 mixture of diastereomers, R_f_ = 0.31 (silica, cyclohexane/CH_2_Cl_2_ = 1:4); IR (ATR): 2972 (w), 2917 (m), ≈2950 (br), 2850 (w), 1734 (m), 1609 (s), 1568 (m), 1453 (m), 1230 (m), 1203 (m), 1163 (s), 1147 (m) cm^−1^; ^1^H-NMR (CDCl_3_): δ 1.37–1.51 (m, 4H, H-3′′, H-7′′a, H-7′′b), 1.58 (s, 3H, H-6′), 1.60 (s, 3H, H-6′), 1.69 (s, 3H, H-5′), 1.70 (s, 3H, H-5′), 1.88–1.95 (m, 2H, H-3′′), 2.28-2.34 (m, 2H, H-2′′), 2.48 (dt, *J* = 14.8, 2H, 7.7 Hz, H-2′), 2.62 (dt, *J* = 15.0, 2H, 5.7 Hz, H-2′); 2.94 (s, br, 2H, H-4′′), 3.06 (s, br, 1H, H-1′′), 3.08 (s, br, 1H, H-1′′), 5.13 (tm, *J* = 7.3 Hz, 1H, H-3’), 5.15 (tm, *J* = 7.3 Hz, 1H, H-3’), 6.04 (td, *J* = 4.3, 0.9 Hz, 1H, H-1’), 6.06 (td, *J* = 4.4, 0.9 Hz, 1H, H-1’), 6.12–6.20 (m, 4H, H-5′′, H-6′′), 7.00 (d, *J* = 1.0 Hz, 1H, H-3), 7.01 (d, *J* = 1.0 Hz, 1H, H-3), 6.12–6.19 (m, 4H, H-5′′, H-6′′), 7.18, 7.19 (2s, 4H, H-6, H-7), 12.43 (2s, 2H, C5-OH), 12.59 (2s, 2H, C8-OH); ^13^C-NMR (CDCl_3_): δ 2 × 18.0 (C-6’), 25.8 (C-5’), 30.3, 30.5 (C-3′′), 33.0, 33.1 (C-2’), 41.6, 41.7 (C-4′′), 43.1, 43.3 (C-2′′), 2 × 46.4 (C-7′′), 46.5, 46.7 (C-1′′), 69.2 (C-1’), 111.6 (C-4a), 111.9 (C-8a), 117.8, 117.9 (C-3’), 2 × 131.4 (C-3), 2 × 132.7 (C-7), 2 × 132.9 (C-6), 135.6 (C-6′′), 2 × 136.0 (C-4’), 138.2, 138.3 (C-5′′), 148.6 (C-2), 2 × 166.9, 167.0 (C-5), 2 × 167.5 (C-8), 174.9, 175.0 (COO), 176.7, 176.8 (C-1), 2 × 178.3 (C-4); MS (ESI^−^) *m*/*z* (%): 837 (5) [2(M − H) + Na]^−^, 408.18 (42) [M]^−^, 407.22 (100) [M − H]^−^; [M]^−^ calculated for C_24_H_24_O_6_: 408.1573. **19**: 1:1 mixture of diastereomers, R_f_ = 0.26 (silica, cyclohexane/CH_2_Cl_2_ = 1:4); IR (ATR): 2973 (w), 2917 (m), ≈2950 (br), 2850 (w), 1733 (m), 1609 (s), 1569 (m), 1452 (m), 1231 (m), 1202 (m), 1165 (s), 1108 (m), 709 (w) cm^−1^; ^1^H-NMR (CDCl_3_): 1.32 (d, *J* = 8.3 Hz, 2H, H-7′′a), 1.39–1.51 (m, 4H, H-3′′, H-7′′b), 1.58 (s, 3H, H-6′), 1.61 (s, 3H, H-6′), 1.69 (s, 3H, H-5′), 1.72 (s, 3H, H-5′), 1.88–2.00 m, 2H, H-3′′), 2.46 (dt, *J* = 14.9, 7.5 Hz, 2H, H-2′), 2.59 (dt, *J* = 15.0, 5.3 Hz, 2H, H-2′), 2.93 (s, br, 2H, H-4′′), 3.04 (dtd, *J* = 8.9, 4.0, 1.1 Hz, 2H, H-2′′), 3.28 (s, br, 2H, H-1′′), 5.11 (tm, *J* = 7.1 Hz, 1H, H-3′), 5.16 (tm, *J* = 7.3 Hz, 1H, H-3′), 5.88 (dd, *J* = 5.7, 2.8 Hz, 1H, H-6′′), 5.90 (dd, *J* = 5.7, 2.8 Hz, 1H, H-6′′), 5.94 (ddd, *J* = ≈7.5, 4.2, 0.8 Hz, 1H, H-1′), 5.96 (ddd, *J* = ≈7.5, 4.6, 0.6 Hz, 2H, H-1′), 6.19 (dd, *J* = 5.7, 3.0 Hz, 1H, H-5′′), 6.22 (dd, *J* = 5.7, 3.1 Hz, 1H, H-5′′), 6.96 (d, *J* = 0.9 Hz, 1H, H-3), 6.99 (d, *J* = 0.9 Hz, 1H, H-3), 7.18 (s, 4H, H-6, H-7), 12.42 (s, 2H, C5-OH), 12.57 (2s, 1H, C8-OH); ^13^C-NMR (CDCl_3_): δ 2 × 18.0 (C-6′), 2 × 25.8 (C-5′), 29.0), 29.3 (C-3′′), 32.9, 33.0 (C-2′), 2 × 42.6 (C-4′′), 2 × 43.4 (C-2′′), 46.0 (C-1′), 49.7, 49.9 (C-7′′), 69.2, 69. 4 (C-1′), 2 × 111.6 (C-4a), 111.9 (C-8a), 117.9, 118.1 (C-3′), 131.3, 131.4 (C-3), 132.0, 132.2 (C-6′′), 132.6, 2 × 132.8, 132.9 (C-6, C-7), 135.8, 135.9 (C-4′), 138.0, 138.1 (C-5′′), 148.6 (C-2), 167.0 (C-5), 167.7 (C-8), 173.6 (COO), 176.5 (COO, C-1), 176.7 (C-1), 178.2 (C-4); more polar diastereomer: MS (ESI^−^) *m*/*z* (%): 408.25 (33) [M]^−^, 407.31 (100) [M − H]^−^; less polar diastereomer: MS (ESI^−^) *m*/*z* (%): 408.17 (43) [M]^−^, 407.21 (100) [M − H]^−^; [M]^−^ calculated for C_24_H_24_O_6_: 408.1573.

*(R)-1-(1,4-Dihydro-5,8-dihydroxy-1,4-dioxonaphthalen-2-yl)-4-methylpent-3-enyl bicyclo[2.2.1]heptane-2-ylacetate* (**20**): 50 µmol Shikonin, 0.12 mmol DCC, 15 µmol DMAP, and 53 µmol bicyclo[2.2.1]heptane-2-ylacetic acid (**20a**); reaction time 16 h; PTLC on silica with cyclohexane/CH_2_Cl_2_ = 1:4 (twice developed) and an additional PTLC on silica (developed twice with cyclohexane/CH_2_Cl_2_ = 1 : 1 and twice with cyclohexane/CH_2_Cl_2_ = 1:2); **20**, yield 19% (sum of isomers). **20**: R_f_ = 0.24 (silica, cyclohexane/CH_2_Cl_2_ = 1:4); IR (ATR): 2948 (m), 2917 (m), ≈2950 (br), 2868 (w), 1738 (s), 1609 (s), 1570 (m), 1453 (m), 1204 (s), 1173 (m), 1159 (s), 783 (m) cm^−1^; ^1^H-NMR (CDCl_3_): 1.06 (m, 1H, H-3′′), 1.13 (m, 1H, H-7′′), 1.15 (m, 1H, H-5′′), 1.24 (m, 1H, H-6′′), 1.32 (dquint, *J* = 9.9, 1.5 Hz, 1H, H-7′′), 1.48 (m, 1H, H-5′′), 1.51 (m, 1H, H-6′′), 1.53 (m, 1H, H-3′′), 1.58 (s, 3H, H-6′), 1.69 (s, 3H, H-5′), 1.91 (quint, *J* = 6.5 Hz, 1H, H-2′′), 1.98 (s, br, 1H, H-1′′), 2.20 (m, 1H, H-α), 2.23 (m, 1H, H-4′′), 2.34 (dd, *J* = 15.2, 7.9 Hz, 1H, H-α), 2.46 (dt, *J* = 15.0, 7.5 Hz, 1H, H-2′), 2.60 (dt, *J* = 15.5, 5.2 Hz, 1H, H-2′), 5.12 (t, *J* = 7.2 Hz, 1H, H-3′), 6.02 (dd, *J* = 7.4, 4.7 Hz, 1H, H-1′), 6.98 (s, 1H, H-3), 7.18 (s, 2H, H-6, H-7), 12.43 (s, 1H, C5-OH), 12.58 (s, 1H, C8-OH); ^13^C-NMR (CDCl_3_): δ 18.0 (C-6′), 25.8 (C-5′), 28.5 (C-5′′), 29.8 (C-6′′), 2 × 33.0 (C-2′), 2 × 35.2 (C-7′′), 36.8 (C-4′′), 37.8, 37.9 (C-3′′), 38.5, 38.6 (C-2′′), 41.1, 41.3 (C-1′′), 2 × 41.3 (C-α), 69.2, 69.3 (C-1’), 111.6 (C-4a), 111.9 (C-8a), 117.9 (C-3’), 131.4, 131.5 (C-3), 132.7 (C-7), 132.8 (C-6), 136.0 (C-4’), 148.5 (C-2), 2 × 166.9 (C-5), 2 × 167.4 (C-8), 171.9 (COO), 2 × 176.8 (C-1), 2 × 178.3 (C-4); MS (ESI^−^) *m*/*z* (%): 869.37 (9) [2(M − H) + Na]^−^, 424.23 (49) [M]^−^, 423.25 (100) [M − H]^−^, [M]^−^ calculated for C_25_H_28_O_6_: 424.1886.

Synthesis of carboxylic acids are described in the Supplementary Material.

### 3.2. Isolation of β,β-dimethylacrylshikonin *(**1**)*

Dried roots of *Onosma paniculata* Bureau & Franchet (Boraginaceae) were acquired in Kunming, China at a medicinal market in October 2003. The identity of the plant material was determined by genomic analysis [46]. Compound **1** was isolated as described previously [8]. In brief, freshly ground roots were extracted with petroleum ether by exhaustive Soxhlet extraction and **1** was isolated by preparative HPLC consisting of a Varian R PrepStar SD-1 (Agilent Technologies, Santa Clara, CA, USA) with Dynamax R solvent delivery system and an absorbance detector model UV-1. A VDSpher 100 RP18 column (250 × 25 mm, 10 µm) (VDS Optilab Chromatographie Technik GmbH, Berlin, Germany) was used as stationary phase. The mobile phase consisted of (A) water and (B) acetonitrile and the following gradient was used. 0−45 min: 70−100% B; 45−60 min: 100% B. **1** was identified by NMR (Varian 400 MHz UnityINOVA spectrometer, Varian, Palo Alto, CA, USA) and CD measurements (Jasco J-715 Spectropolarimeter, Eurisotop, Saint-Aubin CEDEX, France); the purity was determined by HPLC and was 95%.

### 3.3. Cell Culture

Human melanoma cell lines (SBcl2, WM9, WM164, and MUG-Mel2) were cultured in RPMI 1640 medium (Gibco^®^, Thermo Fisher Scientific, Waltham, MA, USA), 2 mM L-glutamine (Gibco^®^), 10% fetal bovine serum (FBS, Gibco^®^), and 1% penicillin-streptomycin (Pen/Strep, Gibco^®^). Human juvenile fibroblasts were isolated from donated foreskin tissue. Pieces were excised and treated with Dispase II (Roche, Vienna, Austria). After enzymatic digestion, cells were grown in high-glucose Dulbecco’s modified Eagle’s medium (DMEM, Gibco^®^) supplemented with 2 mM l-glutamine, 10% FBS, and 1% Pen/Strep. Human epithelial cells (HEK-293) were kept in Dulbecco’s Modified Eagle’s Medium: Nutrient Mixture F-12 (DMEM/F12, Gibco^®^), 2 mM l-glutamine, 10% FBS, and 1% Pen/Strep. Human adult fibroblasts were kindly provided by Ass. Prof. Dr. Beate Rinner (Medical University of Graz) and culture in DMEM, 2mM L-glutamine, and 10% FBS. All cells were kept in a humidified 5% CO_2_ atmosphere at 37 °C and passaged at 90% confluence by trypsinization with 0.25% trypsin–EDTA solution (Gibco^®^).

### 3.4. Sample Preparation for In-Vitro Experiments

All stock solutions were prepared in ethanol, stored at −20 °C and diluted to the respective concentrations in fresh medium before each experiment. Final ethanol concentration was 0.5% in each well. Therefore, control cells represent vehicle-treated cells (0.5% ethanol). This concentration did not affect the cells as shown in benchmark experiments (without ethanol).

### 3.5. Cell Viability Assay (XTT)

Cell viability was analyzed in accordance with the manufacturer’s protocol (Roche Diagnostics, Mannheim, Germany; cell proliferation kit II (XTT), cat. no. 11 465 015 001). This assay is based on the cleavage of the yellow tetrazolium salt XTT (sodium 3′-[1-(phenylaminocarbonyl)-3,4-tetrazolium]-bis(4-methoxy-6-nitro) benzene sulfonic acid hydrate) into an orange formazan dye by metabolic active cells. The color changes only in viable cells and can be directly quantified using a scanning multiwell spectrophotometer. In brief, 5000 cells/well were seeded into 96-well plates (100 µL, flat bottom) and grown for 24 h in a 37 °C, 5% CO_2_ atmosphere before various concentrations of test compounds were added. Control cells were treated with 0.5% ethanol. After 72 h, 50 µL of a freshly prepared XTT solution (5 mL of XTT plus 100 µL of electron coupling reagent) was added, incubated for 2 h or 4 h, and analyzed using a Hidex Sense Microplate Reader (Hidex, Turku, Finland). Compound **1** served as reference (5.0 µM). The assay was performed at least two times, with two or three replicates each.

### 3.6. ApoTox-Glo™ Triplex Assay

This assay was conducted following the manufacturer’s instructions (Promega, Fitchburg, WI, USA, cat. no. G6320). In short, 10,000 cells/well of WM9, WM164, and MUG-Mel2 cells were seeded in 96-well plates (100 µL, white, flat bottom) and treated with various concentrations of **6** for 6 h, 24 h, and 48 h. Staurosporine (10.0 µM) served as positive control for apoptosis induction. Afterwards, 20 µL of a freshly prepared viability/cytotoxicity reagent was added to each well, briefly mixed for 30 sec by orbital shaking (300–500 rpm), and incubated for 30 min at 37 °C. Fluorescence was measured at 400_EX_/505_Em_ (viability) and 485_EX_/520_Em_ (cytotoxicity) by using a Hidex Sense Microplate Reader. Subsequently, 100 µL of a freshly prepared Caspase-Glo^®^ 3/7 reagent was added to each well, briefly mixed by orbital shaking (300–500 rpm, 30 s), and incubated at room temperature for another 30 min. Luminescence was measured using a Hidex Sense Microplate Reader. Each assay was performed at least two times, with three replicates each.

### 3.7. Western Blot Analysis

Cells were seeded at a density of 200,000 c/mL and grown over night before the test compound was added for 24 h. Whole cell protein extracts were prepared with lysis buffer (50 mM Tris-HCl pH 7.4, 150 mM NaCl, 50 mM NaF, 1 mM EDTA, 10% NP-40, 1% Triton-X, and protease inhibitors), subjected to SDS-PAGE (10 or 12%), and blotted onto PVDF membranes (Roth, Karlsruhe, Germany). Primary antibodies against PARP, phosphoH2AX (Ser139), and β-actin were purchased from Cell Signaling Technology (Danvers, MA, USA). Blots were developed using corresponding horseradish peroxidase-conjugated secondary antibodies (Dako, Jena, Germany) at room temperature for 1 h and the Amersham™ ECL™ prime Western blotting detection reagent (GE Healthcare, in accordance with the manufacturer’s protocol). Chemiluminescence signals were detected by the ChemiDocTouch Imaging System (BioRad Laboratories Inc., Herkules, CA, USA). Images were processed using ImageLab 5.2 Software (BioRad Laboratories Inc.) and normalized to their loading controls.

### 3.8. Caspase 3 Cleavage Measured by FACS

Cells were seeded at a density of 200,000 c/mL and grown over night before the test compound was added for 24 h. After incubation with **6** in presence or absence of a caspase inhibitor (Z-VAD-FMK), cells were harvested by trypsinization and fixed and permeabilized with Cytofix/CytopermTM solution (BD Biosciences, Frankling Lakes, NJ, USA). The pellet was resuspended in Perm/Wash^TM^ buffer and stained with FITC-conjugated monoclonal active caspase 3 antibody (BD Biosciences). Finally, cells were resuspended in 1 mL PBS and analyzed with a LSRII™ (BD Biosciences) flow cytometer equipped with a 488 nm argon ion laser and a 635 nm red diode laser (BD Biosciences). Untreated cells were used as a negative control.

### 3.9. CytoTox 96^®^ Non-Radioactive Cytotoxicity Assay (LDH Assay)

Cell membrane integrity was analyzed using the CytoTox 96^®^ Non-Radioactive Cytotoxicity Assay (G1780, Promega, Fitchburg, WI, USA) according to the manufacturer’s instructions. Aliquots (100 µL) of 100,000 c/mL were seeded into 96-well plates (flat bottom), grown for 24 h, and treated with various concentrations of **6** (2.5 µM, 5.0 µM, 7.5 µM, and 10.0 µM) for 6 h, 24 h, and 48 h. To measure maximal LDH release, 10 µL of 10X Lysis Solution were added to control wells 45 min before adding the CytoTox96^®^ Reagent. To measure the amount of released LDH, 50 µL of each well were transferred to a fresh 96-well plate and 50 µL of CytoTox96^®^ Reagent were added followed by a 30 min incubation period. Finally, 50 µL of Stop Solution was added and absorbance was recorded at 490 nm (Hidex Sense Microplate Reader). Absorbance values were corrected by background values and the percentage of LDH release was calculated using the following formula. 100 × experimental LDH release (OD490)/maximum LDH release (OD490).

### 3.10. Cell Cycle Analysis

Cells (200,000 c/mL) were treated with the respective IC_50_ concentration for 24 h and harvested by trypsinization. Afterwards, they were fixed with 70% ice cold ethanol for 10 min and at 4 °C. After washing with PBS, the cell pellet was resuspended in PI-staining buffer (50 µL/mL PI, RNAse, Beckman Coulter, Brea, CA, USA) and incubated for 15 min at 37 °C. Cell cycle distribution was analyzed using a LSRII™ (BD Biosciences) flow cytometer.

### 3.11. Statistical Analysis

Statistical analysis was carried out using SigmaPlot 13.0 (Systat Software Inc., Chicago, IL, USA). IC_50_ values were determined using the four-parameter logistic curve, at least five concentrations of the test compound, and two different cell passages each tested in two or three independent wells. Results are expressed as mean ± SEM. *P* values < 0.05 were considered as statistically significant.

## 4. Conclusions

In this study, eighteen novel shikonin derivatives and one known derivative were synthesized. Afterwards, their cytotoxic effects against several melanoma and skin fibroblast cell lines were investigated. Compound **1** was chosen as reference because it was the most active isolated derivative so far. To keep close to this lead compound, the two methyl groups of the side chain of **1** were formally connected with different numbers of methylene groups and the influence of the double bond and the spacing CH resp. CH_2_ group was investigated as well. In addition, three cycloalkenyl and three bicyclic derivatives were prepared to examine the effect of unsaturation and space filling with an additional methylene moiety.

In general, all derivatives exhibited cytotoxic activity, but most of them were less active than **1**. For example, the prepared cycloalkylideneacetates **2** to **5** and cycloalkylacetates **7** to **10** were less active against the metastatic cell lines than **1**. Furthermore, neither the removal of the spacing CH resp. CH_2_ group nor bicyclic derivatives led to an overall increase of cytotoxicity compared to **1**. However, the cyclopropane derivative **6** was considerably more active against the metastatic cell lines WM164 and MUG-Mel2 than **1**. The effect was also stronger than the effect of the already known derivative **11**. Therefore, **6** was pharmacologically investigated in more detail. We could show that **6** led to apoptosis induction after 24 h while the cell membrane was not damaged within this time and at the concentration used. Apoptosis was induced caspase dependently as shown by the ApoToxGlo™ assay, FACS experiments in combination with a caspase inhibitor, and Western blot experiments. Moreover, **6** led to double-stranded DNA breaks as shown by phosphorylation of H2AX which can lead to subsequent apoptosis induction. However, **6** did not lead to cell cycle arrest as often caused by H2AX phosphorylation. In summary, the results demonstrate that **6** could represent a novel lead compound to develop antimelanoma compounds.

## Figures and Tables

**Table 1:** List of investigated shikonin esters.

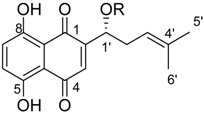				
**Cpd.**	**R**		**Cpd.**	**R**
1	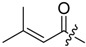		11	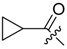
2	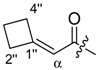		12	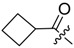
3	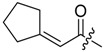		13	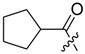
4	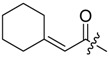		14	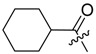
5	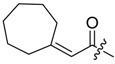		15	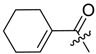
6	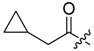		16	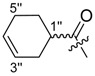
7	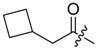		17	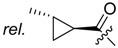
8	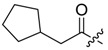		18	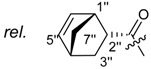
9	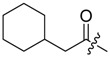		19	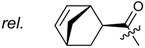
10	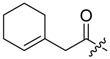		20	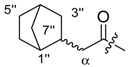

**Table 2:** IC_50_ values (µM) after 72 h treatment with **1** or **6** (mean ± SEM, *n* = 4). IC_50_ values were determined using the four-parameter logistic curve and individual values of all independent experiments.

Compound	SBcl2	WM9	WM164	MUG-Mel2
**1**	1.1 ± 0.1	2.7 ± 0.3	8.3 ± 0.3	7.2 ± 0.5
**6**	0.9 ± 0.7	3.2 ± 0.8	4.9 ± 1.7	3.2 ± 0.3

## Supplementary Materials

The following are available online at http://www.mdpi.com/1420-3049/23/11/2820/s1. Studies on the enantiomeric purity and associated pharmacological effects of **1**; NMR-spectra (^1^H and ^13^C or HMBC) of the new shikonin derivatives; synthesis of cycloalkylidenacetic acids **2a** to **5a** and cyclobutylacetic acid (**7a**); ^1^H-NMR spectra of synthesized acids; HPLC runs of synthesized compounds; results of the XTT assay.
