# Supplementary material for: Synthesis of Novel Shikonin Derivatives and Pharmacological Effects of Cyclopropylacetylshikonin on Melanoma Cells
Source: Molecules. 2018 Oct 30;23(11):2820. doi: 10.3390/molecules23112820 (PMC6278577; doi:10.3390/molecules23112820)
Supplement: Supplementary file 1 [file molecules-23-02820-s001.pdf]

molecules

Supplementary Material

Synthesis of novel shikonin derivatives and pharmacological effects of cyclopropylacetylshikonin on melanoma cells

Christin Durchschein [1], Antje Hufner [2], Beate Rinner [3], Alexander Stallinger [3], Alexander Deutsch [4], Birgit Lohberger [5], Rudolf Bauer [1]*, Nadine Kretschmer [1]

[1] Institute of Pharmaceutical Sciences, Department of Pharmacognosy, University of Graz, Universitaetsplatz 4, 8010 Graz, Austria
[2] Institute of Pharmaceutical Sciences, Department of Pharmaceutical Chemistry, University of Graz, Universitaetsplatz 1, 8010 Graz, Austria
[3] Division of Biomedical Research, Medical University of Graz, Roseggerweg 48, 8036 Graz, Austria
[4] Division of Hematology, Medical University of Graz, Auenbruggerplatz 15, 8036 Graz, Austria
[5] Department of Orthopedics and Trauma, Medical University Graz, Auenbruggerplatz 5, 8036 Graz, Austria
*** Correspondence: rudolf.bauer@uni-graz.at; Tel.: +43 316 380 8700**

Content

1. Studies on the enantiomeric purity and associated pharmacological effects of **1**

2. NMR-spectra (^{1}H and ^{13}C or HMBC) of novel shikonin derivatives **2-10** and **12-20**

3. Syntheses of cycloalkylidenacetic acids **2a** to **5a** and cyclobutylacetic acid (**7a**)

4. ^{1}H NMR-spectra of synthesized acids

5. HPLC runs of synthesized compounds

6. Results of the XTT assay of compounds **1-20**

1. Studies on the enantiomeric purity and associated pharmacological effects of 1

Shikonin and derivatives possess a chiral center in the side chain. In nature, the enantiomeric ratios of shikonin (*R*-isomer) and alkannin (*S*-isomer) varies. Therefore, we determined the optical purity of the isolated shikonins as well as synthesized derivatives by chiral HPLC.

1.1. General

Chiral separations were performed using an Agilent 1100 Series Liquid Chromatograph (Agilent Technologies, Waldbronn, Germany) equipped with an autosampler and a VWL detector. UV-data were collected at 520 nm. Experiments were carried out at ambient temperature under isocratic conditions with a flow rate of 1.0 ml/min and an injection volume of 15 µl. Data evaluation was performed using a ChemStation for LC 3D Systems Rev. B. 04.03 (Agilent Technologies, Waldbronn, Germany) software. A Chiralcel OD, 250 x 4.6 mm, 3.5 µm from Daicel Chemical Industries (Osaka, Japan) served as CSP with immobilized cellulose tris (3,5-dimethylphenylcarbamate) as chiral selector. Mobile phase consisted of *n*-hexane / 2-propanol = 3 : 1 (v/v).

1.2. Identification of shikonin and alkannin

An authentic sample of previously isolated **1** [8] was hydrolyzed with diluted sodium hydroxide solution [S1]. Chiral HPLC [S2] of the isolated product revealed 70% shikonin and 30% alkannin. The shikonin batch supplied from Chengdu Biopurify Phytochemicals Ltd., Chengdu, People's Republic of China, which was also used for syntheses, and, in addition, an alkannin lot supplied from Chengdu Push Bio-Technology Co. Ltd., Chengdu, People's Republic of China were submitted to the same analysis. Shikonin turned out to consist of 100% *R*-isomer, alkannin was almost a racemate (*R* : *S* = 49 : 51).

References:

S1: Pekin, G.; Ganzera, M.; Senol, S.; Bedir, E.; Korkmaz, K.S.; Stuppner, H. Determination of Naphthazarin Derivatives in Endemic Turkish Alkanna Species by Revered Phase High Performance Liquid Chromatography. *Planta Med* **2007**, *73*, 267-272, DOI: 10.1055/s-2007-967110.

S2: Ikeda, Y.; Ishida, N.; Fukaya, C.; Yokoyama, K.; Tabata, M.; Fukui, H.; Honda, G. Determination of the Ratio between Optical Isomers, Shikonin and Alkannin by High Performance Liquid Chromatography Analysis. *Chem Pharm Bull* **1991**, *39*, 2351-2153.

1.3. Determination of enantiomeric purity of ß,ß-dimethylacrylshikonin samples

To investigate the influence of the chiral center on the cytotoxicity, different hydrolysates of **1** were prepared and analyzed in accordance to the method above (Table S1). These investigations also show that the enantiomeric ratio did not change during synthesis.

Table S1: Enantiomeric ratios of samples of **1**. Racemic *ß,ß*-dimethylacrylshikonin was prepared by acylation of racemic shikonin/alkannin mixture with *ß,ß*-dimethylacrylic acid according to the procedure in the plain text.

Source of 1	Ratio of enantiomers (after hydrolysis)
Isolated from *O. paniculata* [8]	70% *R*-isomer and 30% *S*-isomer
synthesized from 100% shikonin	100% *R*-isomer
synthesized from alkannin	49% *R*-isomer and 51% *S*-isomer

*1.4. Influence of enantiomeric purity of **1** on cytotoxicity*

Cytotoxicity was investigated using WM164 melanoma cells and pure *R*-isomer, 49% *R*-isomer and two 70:30 mixtures of *R*-isomer (a: mixture of 100% and 49% R-isomer to yield 70/30; b: isolated **1**). Melanoma cells were treated with these compounds up to 10.0 µM and 72 h of incubation (Figure S1). We did not detect significant differences in the cytotoxicity indicating that the chiral center as well as the synthesis process has no influence on the activity.

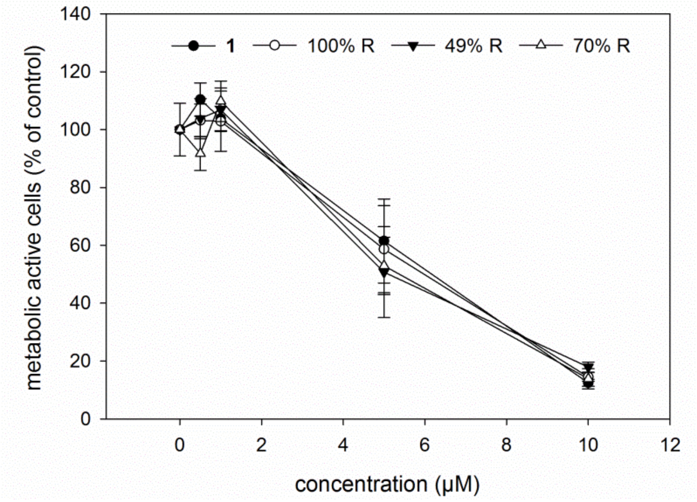

Figure S1. Cytotoxicity of the isolated **1** compared to 100% synthesized *R*-isomer of **1** (100% R), 49% *R*-isomer (49% R) and "mixed" 70% *R*-isomer (70% R) as determined by the XTT viability assay and after 72 h incubation with different concentration of the compounds (WM164 cells, mean ± sem, *n* = 6).

2. NMR-spectra (^{1}H and ^{13}C or HMBC) of the new shikonin derivatives 2 to 10 and 12 to 20

^{1}H and ^{13}C NMR spectra were recorded on Varian 400 MHz UnityINOVA spectrometer (400 and 100 MHz, respectively) using deuterated chloroform ($CDCl_3$) as solvent and TMS as internal standard.

After acylation of shikonin, the side chain was proven by NMR: The ^{1}H NMR signal of H-1′ of the side chain was shifted from 4.92 ppm in shikonin to 6.0 - 6.1 ppm in the esters **2** to **20**, whereas, the signals of the aromatic and the phenolic hydrogen atoms remained almost constant. In the HMBC spectra a cross peak from ester carbonyl carbon to H-1′ of the shikonin moiety proves the esterification at the side chain of shikonin.

NMR-Spectra:

^{1}H-NMR spectrum of (*R*)-1-(1,4-dihydro-5,8-dihydroxy-1,4-dioxonaphthalen-2-yl)-4-methylpent-3-enyl 2-cyclobutylidenacetate (**2**):

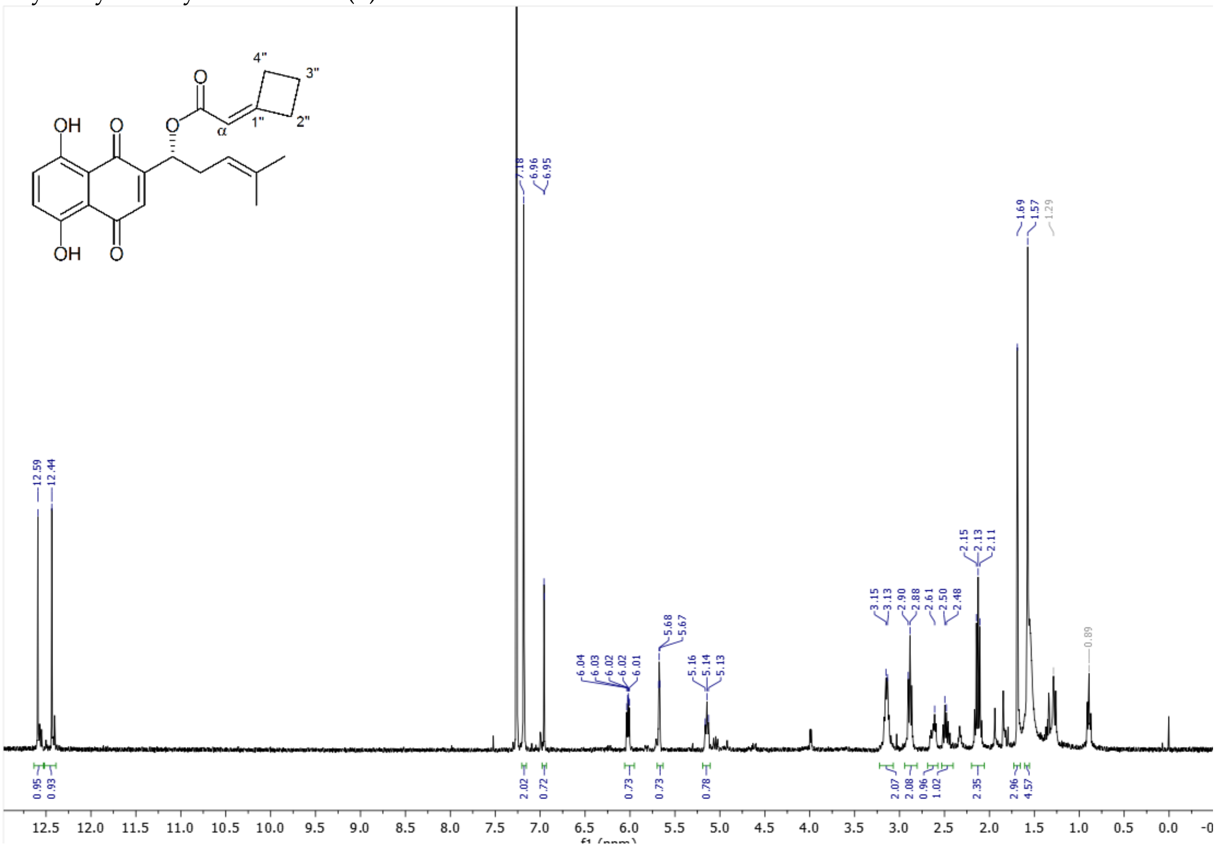

HMBC spectrum of (*R*)-1-(1,4-dihydro-5,8-dihydroxy-1,4-dioxonaphthalen-2-yl)-4-methylpent-3-enyl 2-cyclobutylidenacetate (**2**):

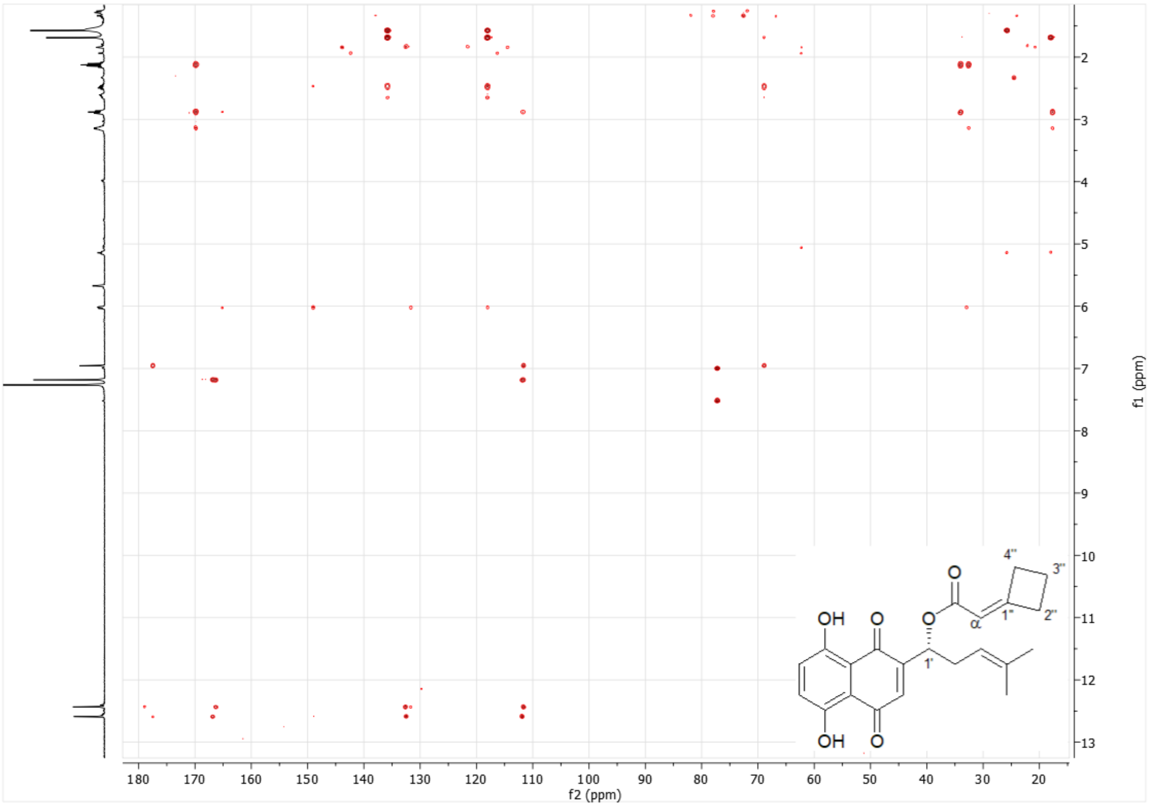

^{1}H-NMR spectrum of (*R*)-1-(1,4-dihydro-5,8-dihydroxy-1,4-dioxonaphthalen-2-yl)-4-methylpent-3-enyl 2-cyclopentylidenacetate (**3**):

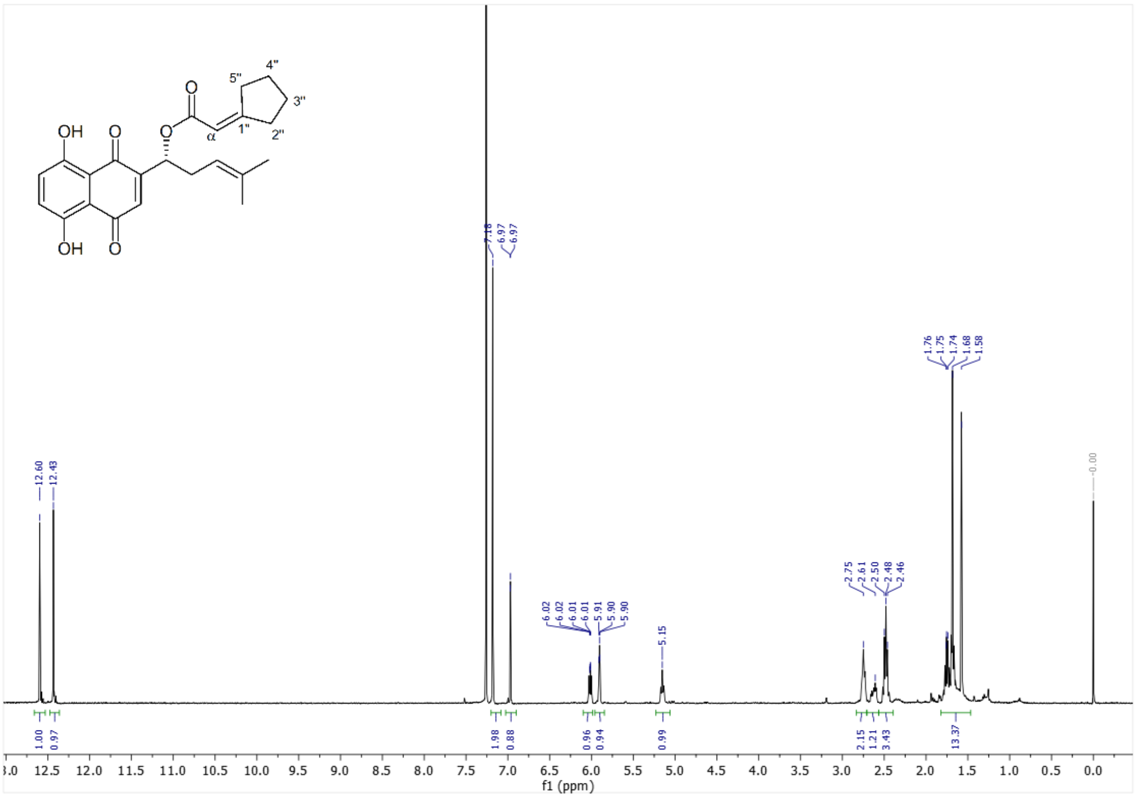

^{13}C-NMR spectrum of (*R*)-1-(1,4-dihydro-5,8-dihydroxy-1,4-dioxonaphthalen-2-yl)-4-methylpent-3-enyl 2-cyclopentylidenacetate (**3**):

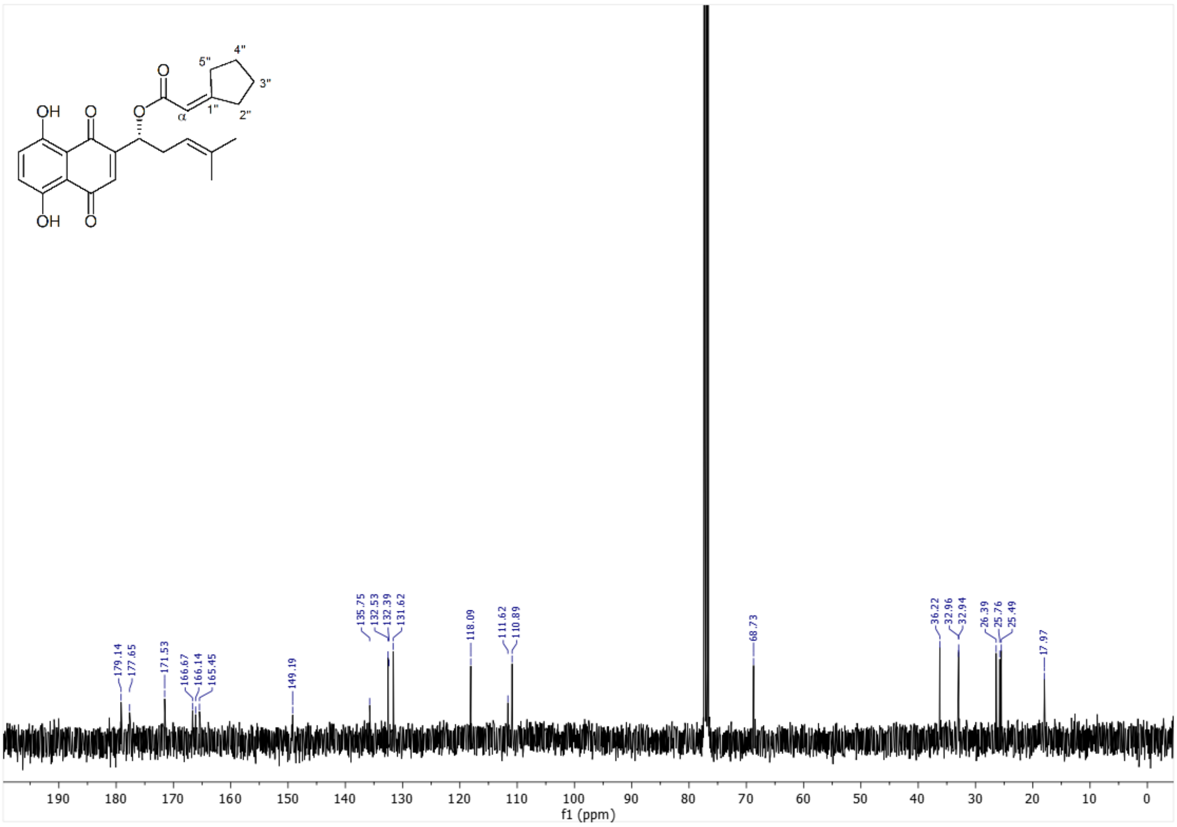

HMBC spectrum of (*R*)-1-(1,4-dihydro-5,8-dihydroxy-1,4-dioxonaphthalen-2-yl)-4-methylpent-3-enyl 2-cyclopentylidenacetate (**3**):

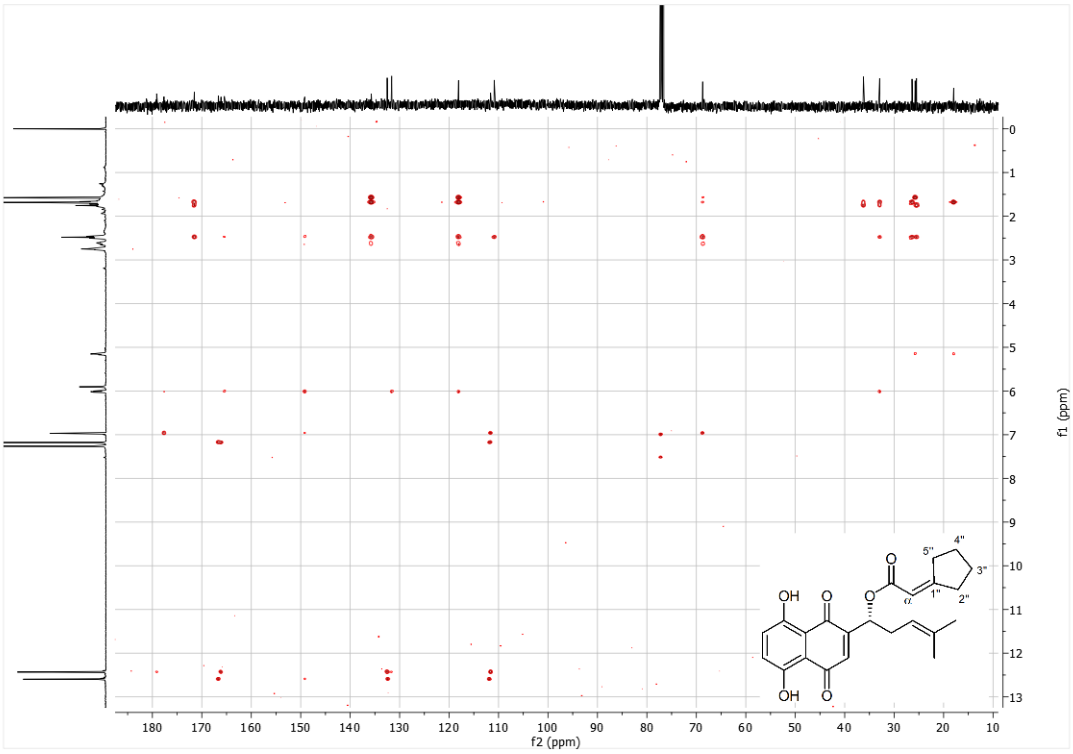

^{1}H-NMR spectrum of (*R*)-1-(1,4-dihydro-5,8-dihydroxy-1,4-dioxonaphthalen-2-yl)-4-methylpent-3-enyl 2-cyclohexylidenacetate (**4**):

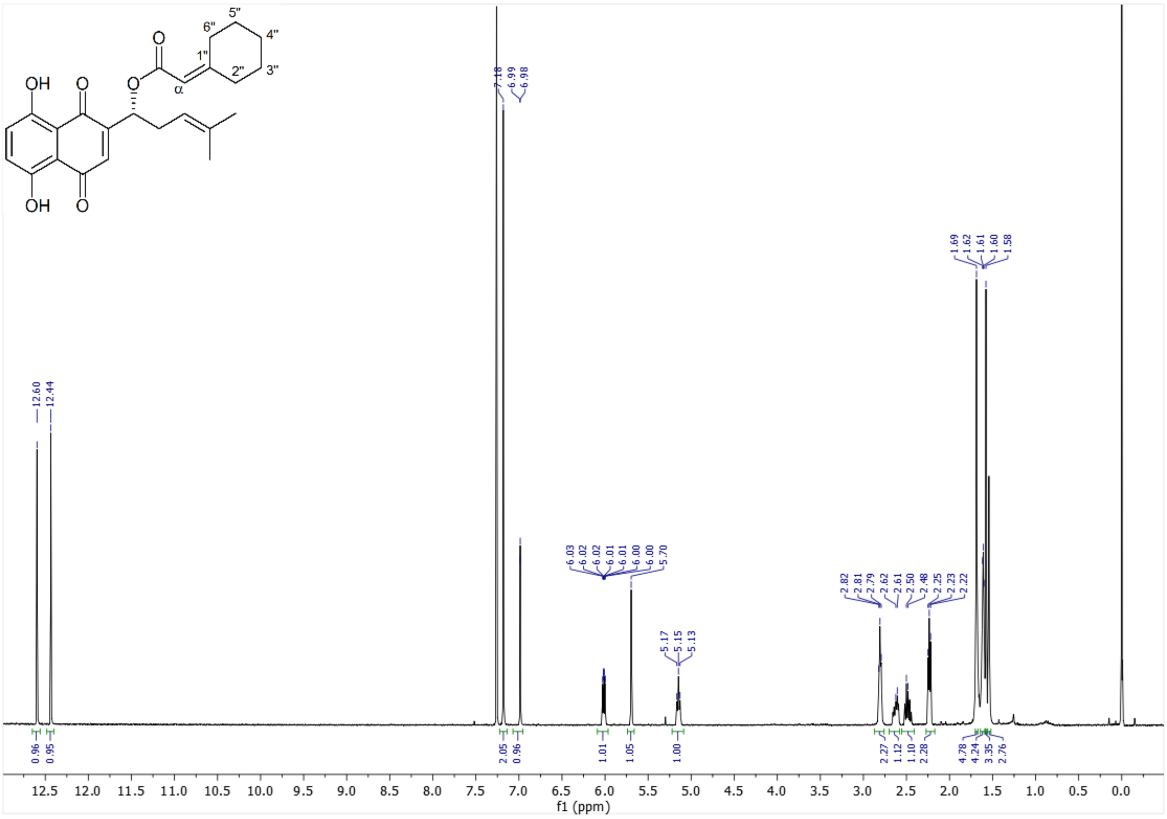

^{13}C-NMR spectrum of (*R*)-1-(1,4-dihydro-5,8-dihydroxy-1,4-dioxonaphthalen-2-yl)-4-methylpent-3-enyl 2-cyclohexylidenacetate (**4**):

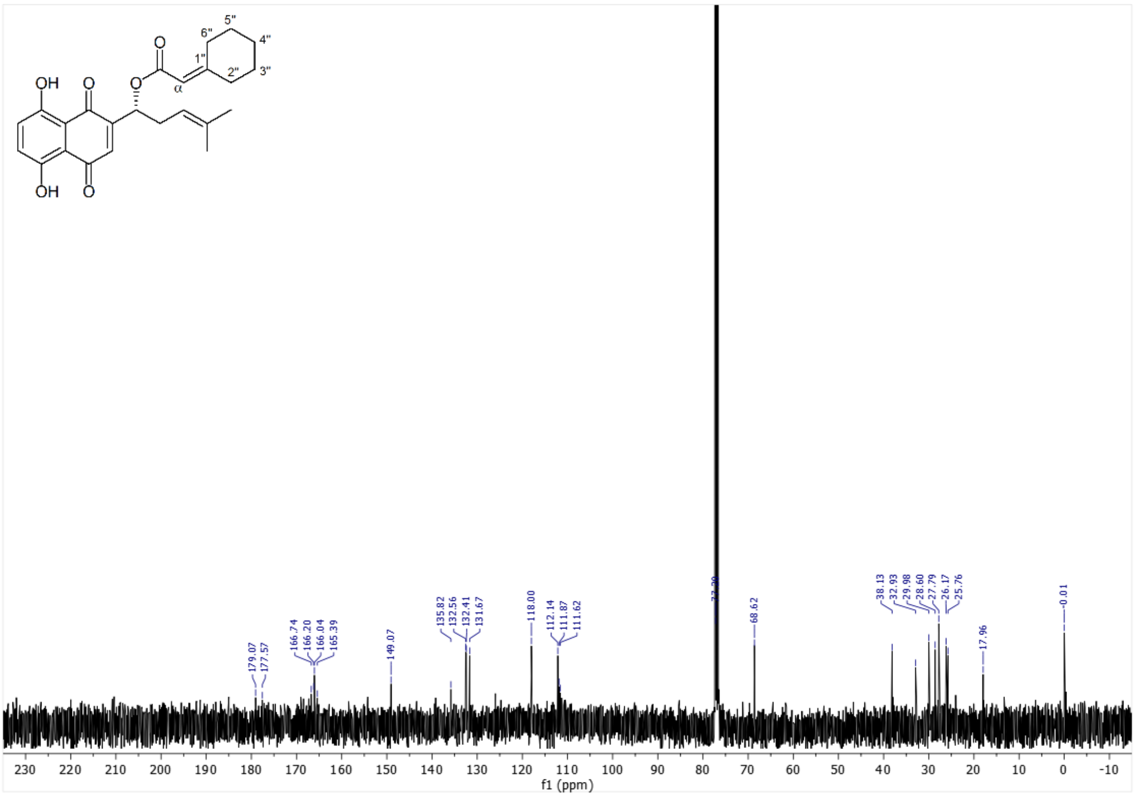

HMBC spectrum of (*R*)-1-(1,4-dihydro-5,8-dihydroxy-1,4-dioxonaphthalen-2-yl)-4-methylpent-3-enyl 2-cyclohexylidenacetate (**4**):

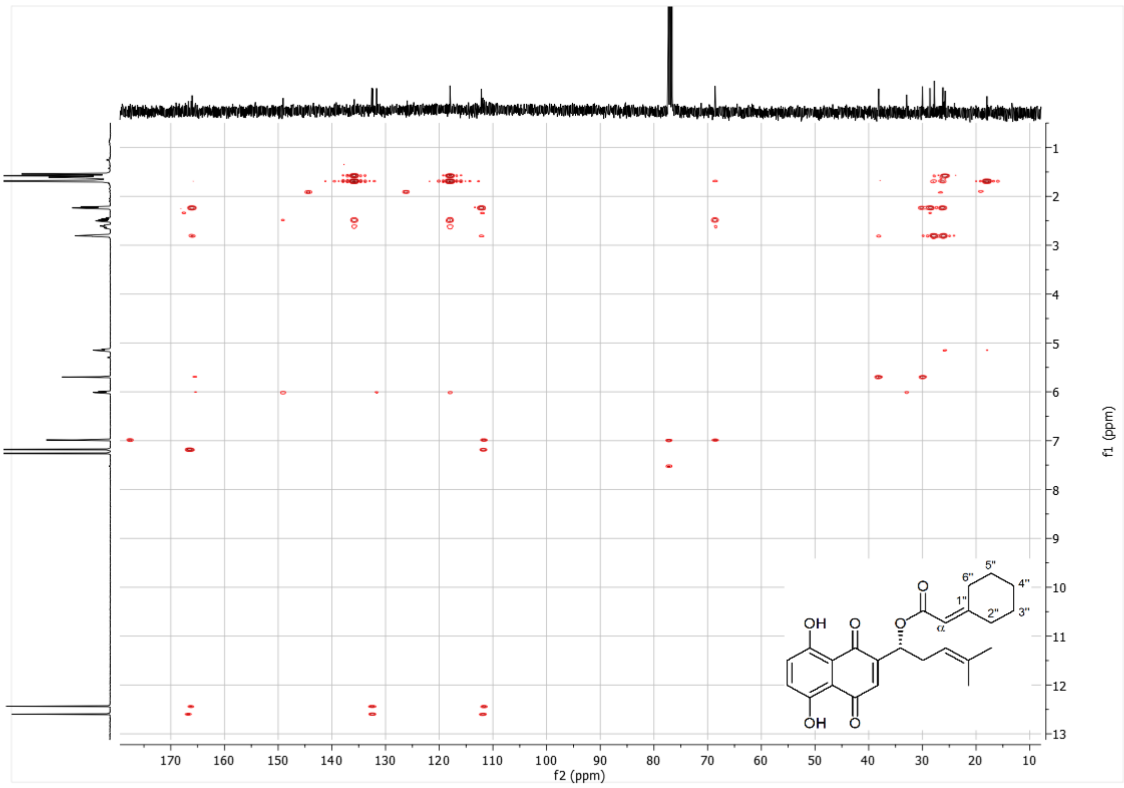

^{1}H-NMR spectrum of (*R*)-1-(1,4-dihydro-5,8-dihydroxy-1,4-dioxonaphthalen-2-yl)-4-methylpent-3-enyl 2-cycloheptylidenacetate (**5**):

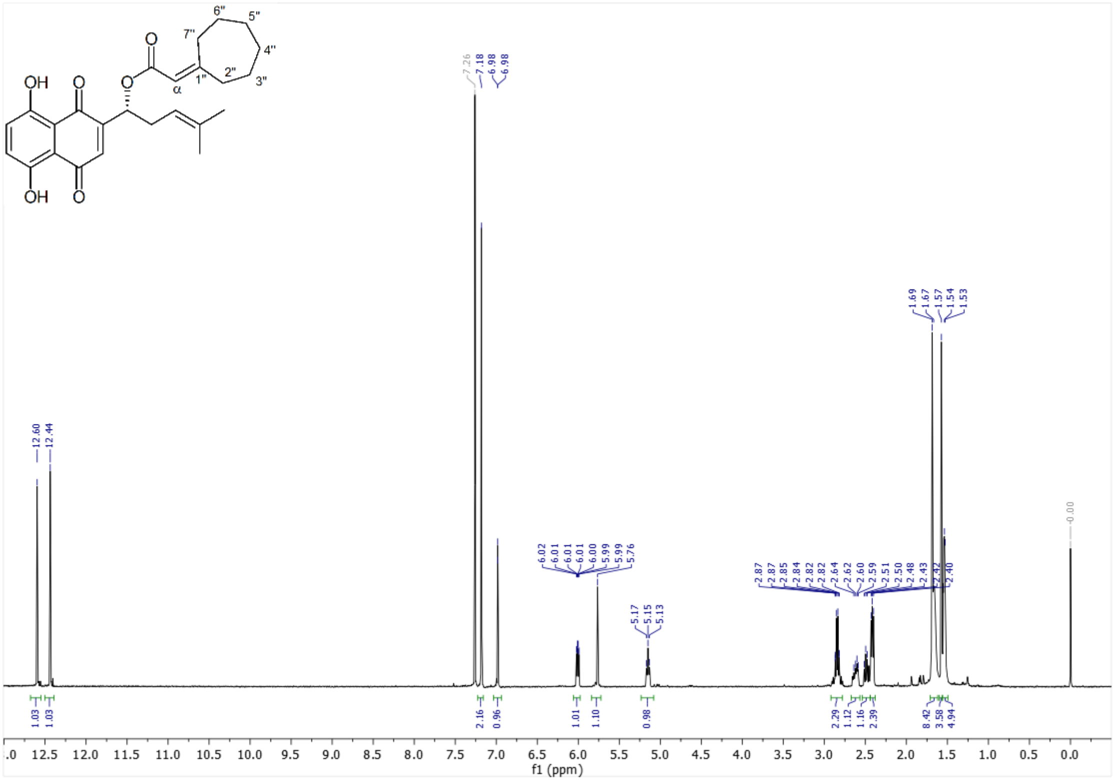

^{13}C-NMR spectrum of (*R*)-1-(1,4-dihydro-5,8-dihydroxy-1,4-dioxonaphthalen-2-yl)-4-methylpent-3-enyl 2-cycloheptylidenacetate (**5**):

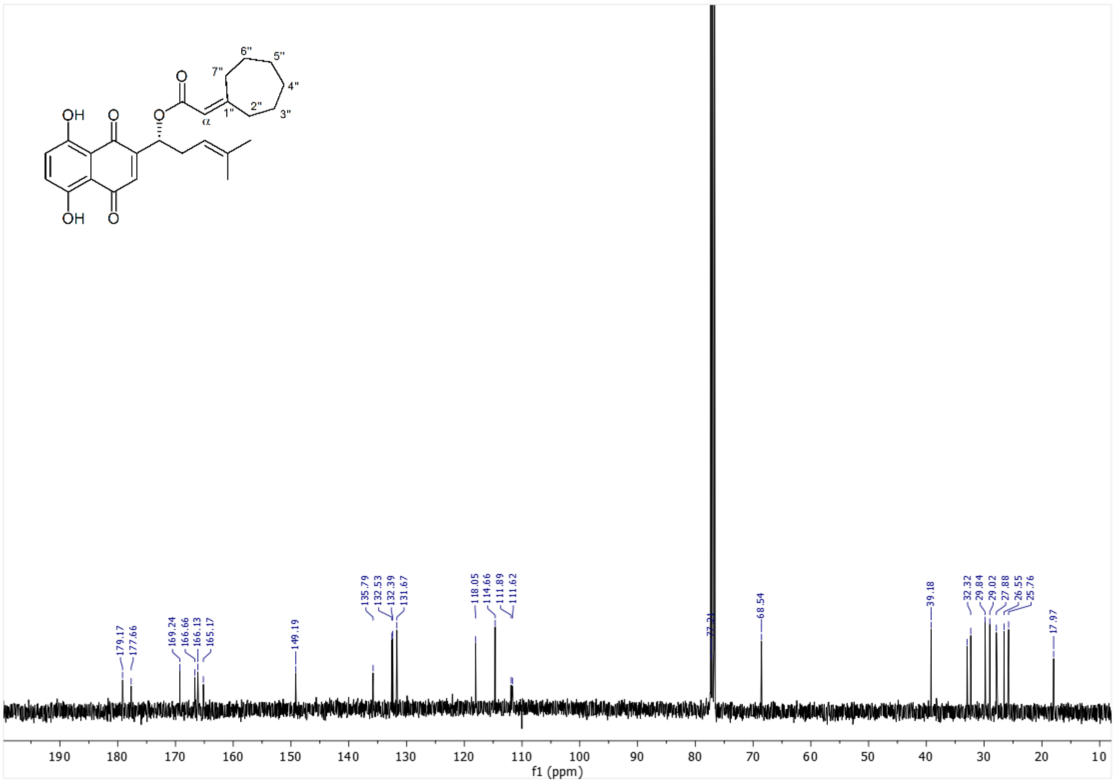

^{1}H-NMR spectrum of (*R*)-1-(1,4-dihydro-5,8-dihydroxy-1,4-dioxonaphthalen-2-yl)-4-methylpent-3-enyl cyclopropylacetate (**6**):

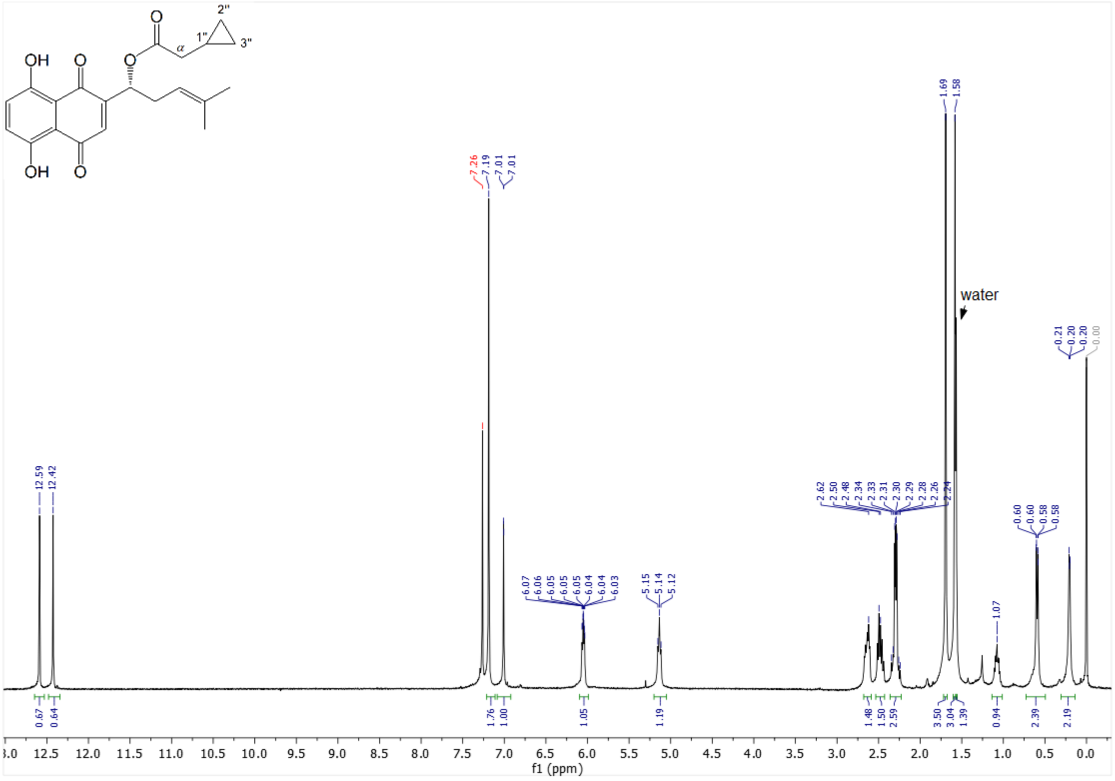

^{13}C-NMR spectrum of (*R*)-1-(1,4-dihydro-5,8-dihydroxy-1,4-dioxonaphthalen-2-yl)-4-methylpent-3-enyl cyclopropylacetate (**6**):

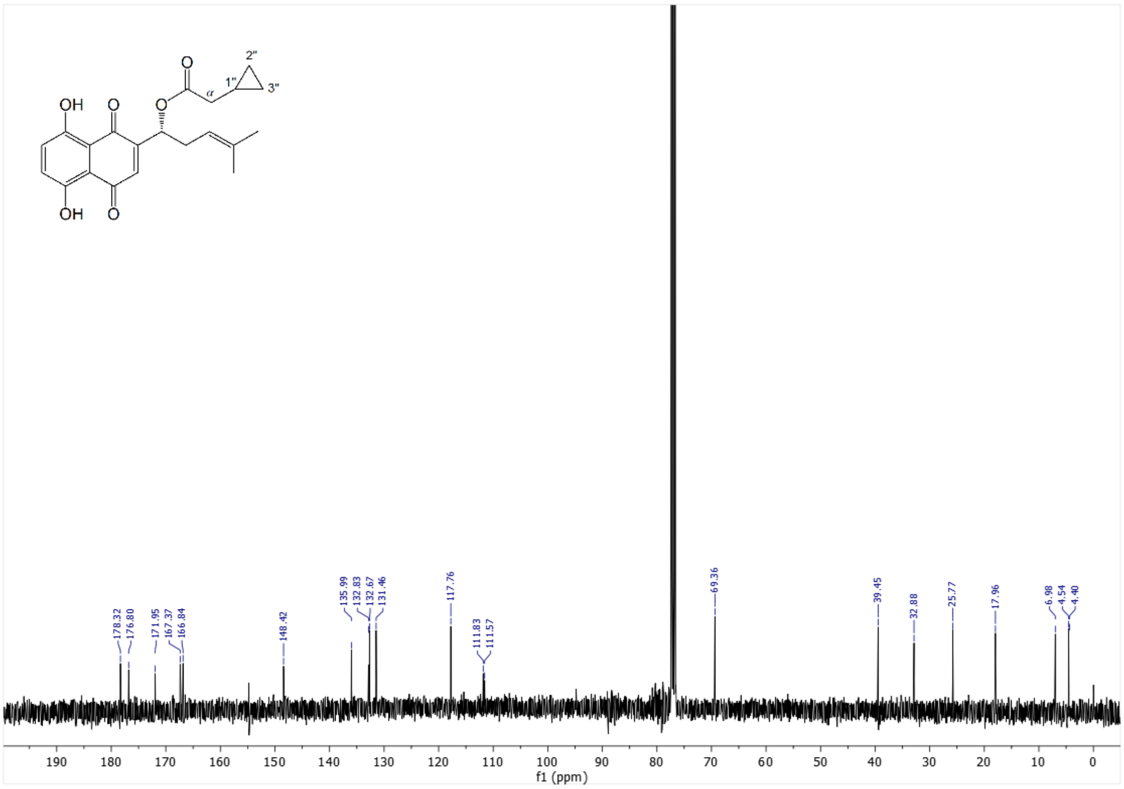

^{1}H-NMR spectrum of (*R*)-1-(1,4-dihydro-5,8-dihydroxy-1,4-dioxonaphthalen-2-yl)-4-methylpent-3-enyl cyclobutylacetate (**7**):

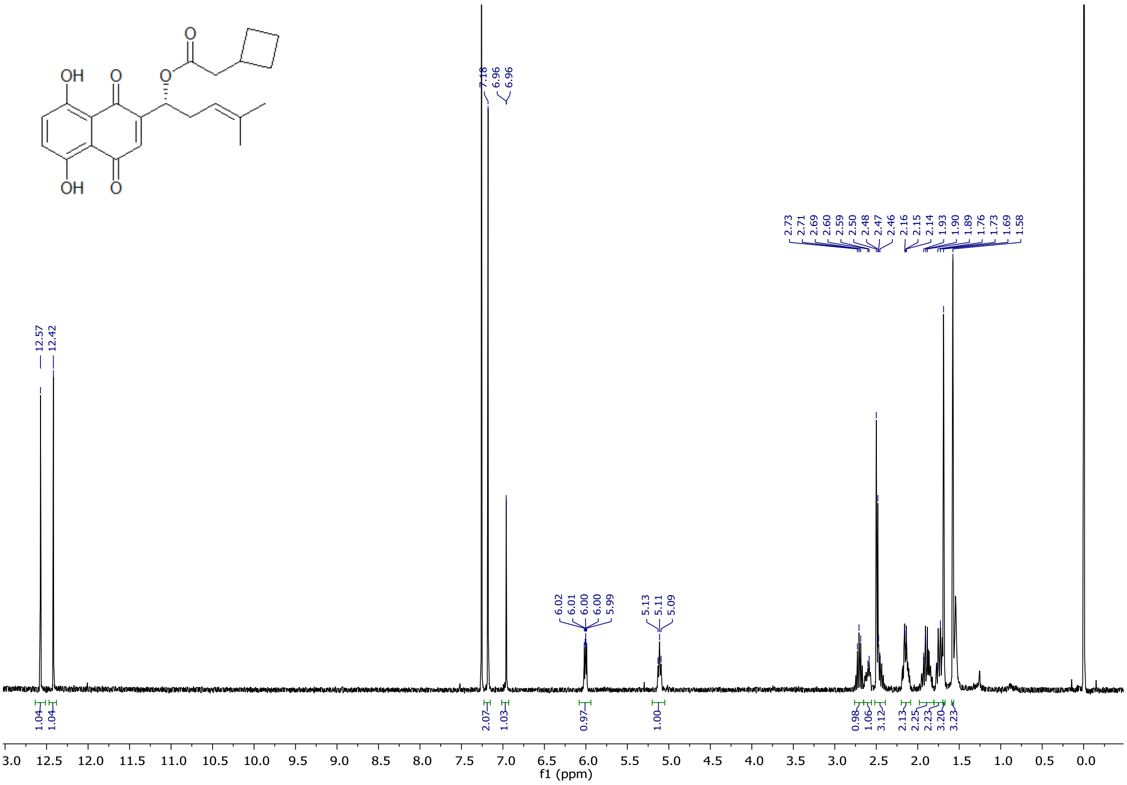

^{13}C-NMR spectrum of (*R*)-1-(1,4-dihydro-5,8-dihydroxy-1,4-dioxonaphthalen-2-yl)-4-methylpent-3-enyl cyclobutylacetate (**7**):

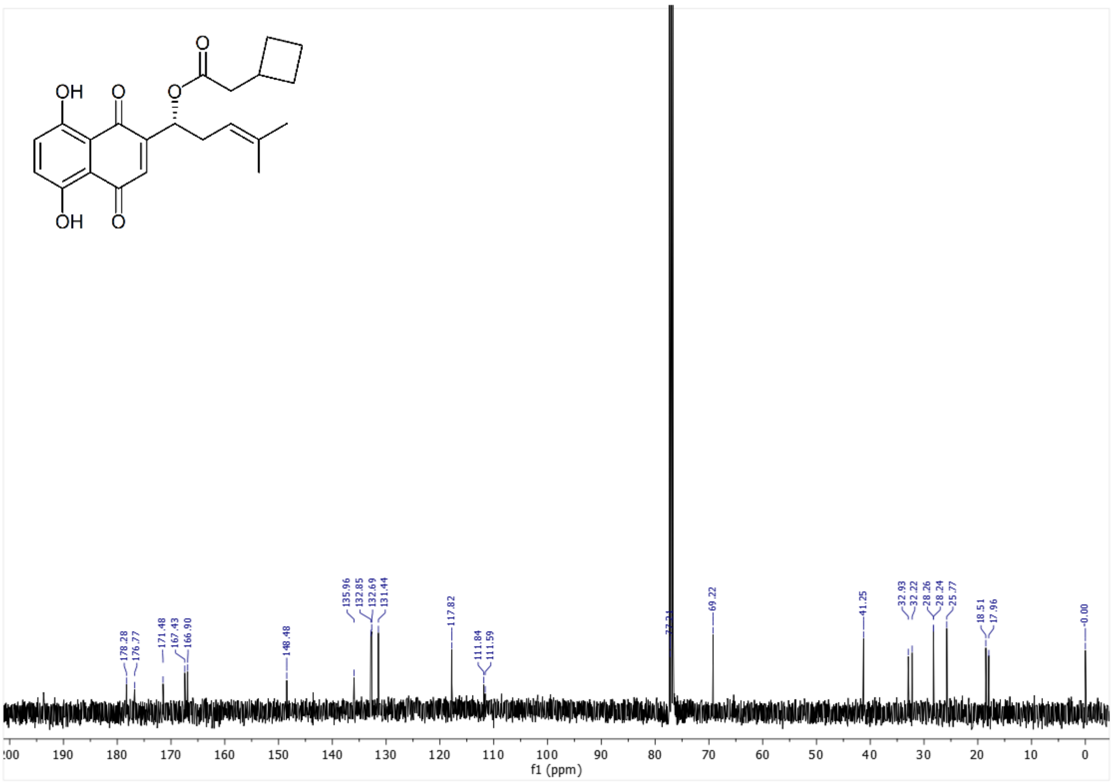

^{1}H-NMR spectrum of (*R*)-1-(1,4-dihydro-5,8-dihydroxy-1,4-dioxonaphthalen-2-yl)-4-methylpent-3-enyl cyclopentylacetate (**8**):

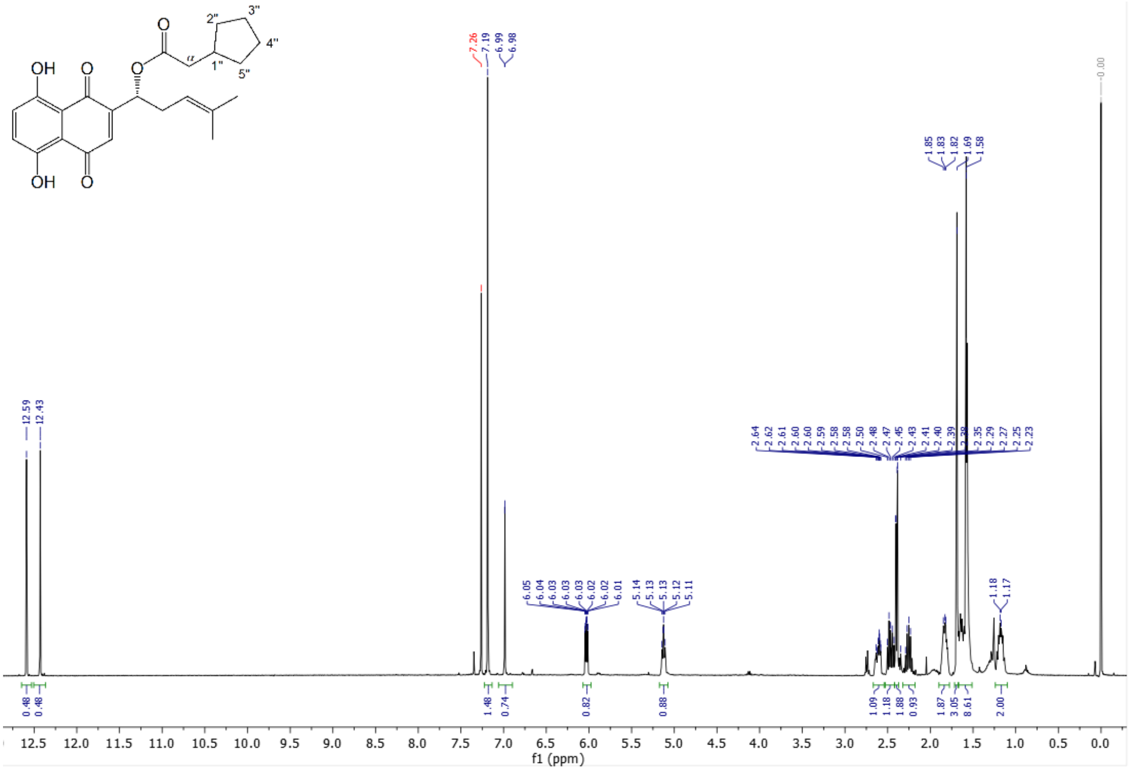

^{1}H-NMR spectrum of (*R*)-1-(1,4-dihydro-5,8-dihydroxy-1,4-dioxonaphthalen-2-yl)-4-methylpent-3-enyl cyclopentylacetate (**8**):

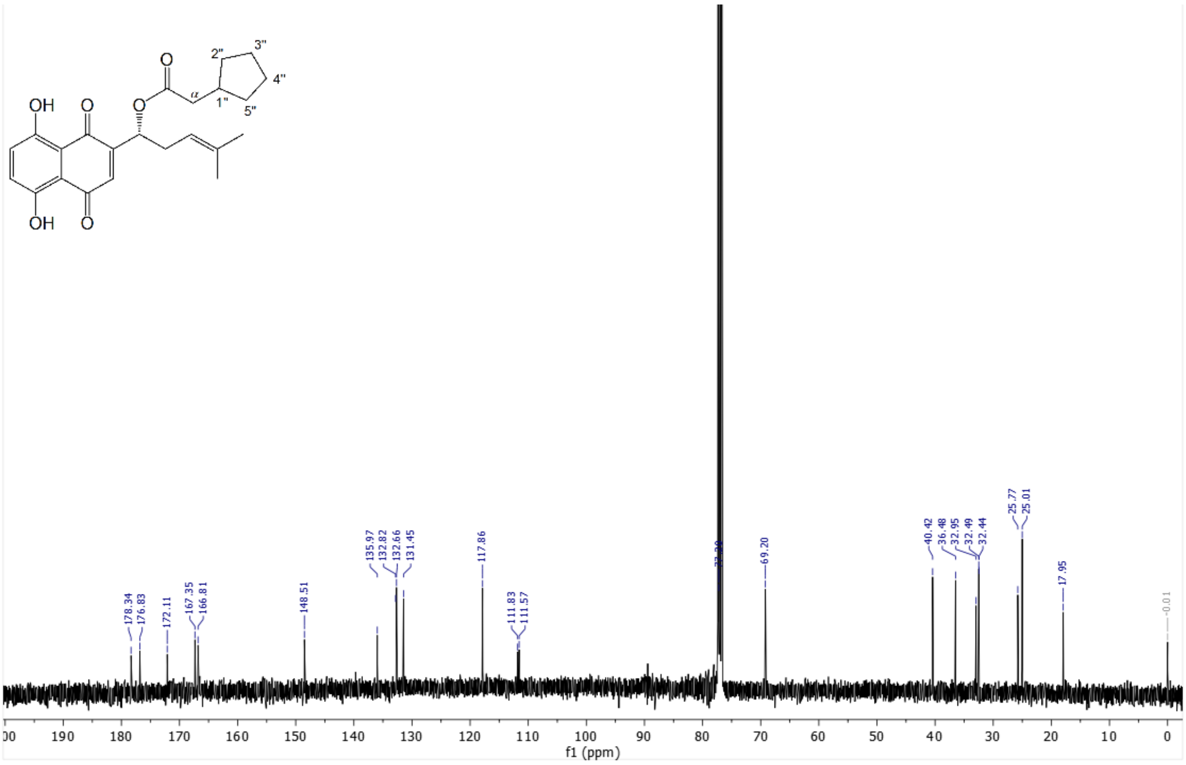

^{1}H-NMR spectrum of (*R*)-1-(1,4-dihydro-5,8-dihydroxy-1,4-dioxonaphthalen-2-yl)-4-methylpent-3-enyl cyclohexylacetate (**9**):

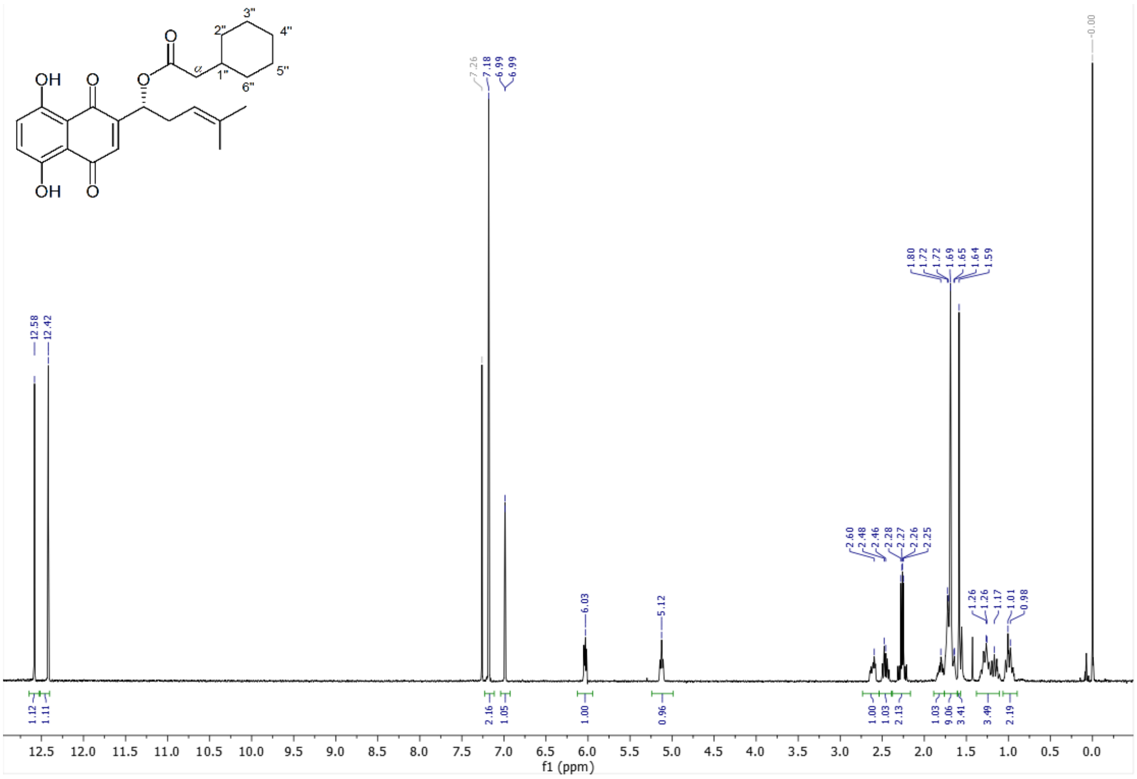

^{13}C-NMR spectrum of (*R*)-1-(1,4-dihydro-5,8-dihydroxy-1,4-dioxonaphthalen-2-yl)-4-methylpent-3-enyl cyclohexylacetate (**9**):

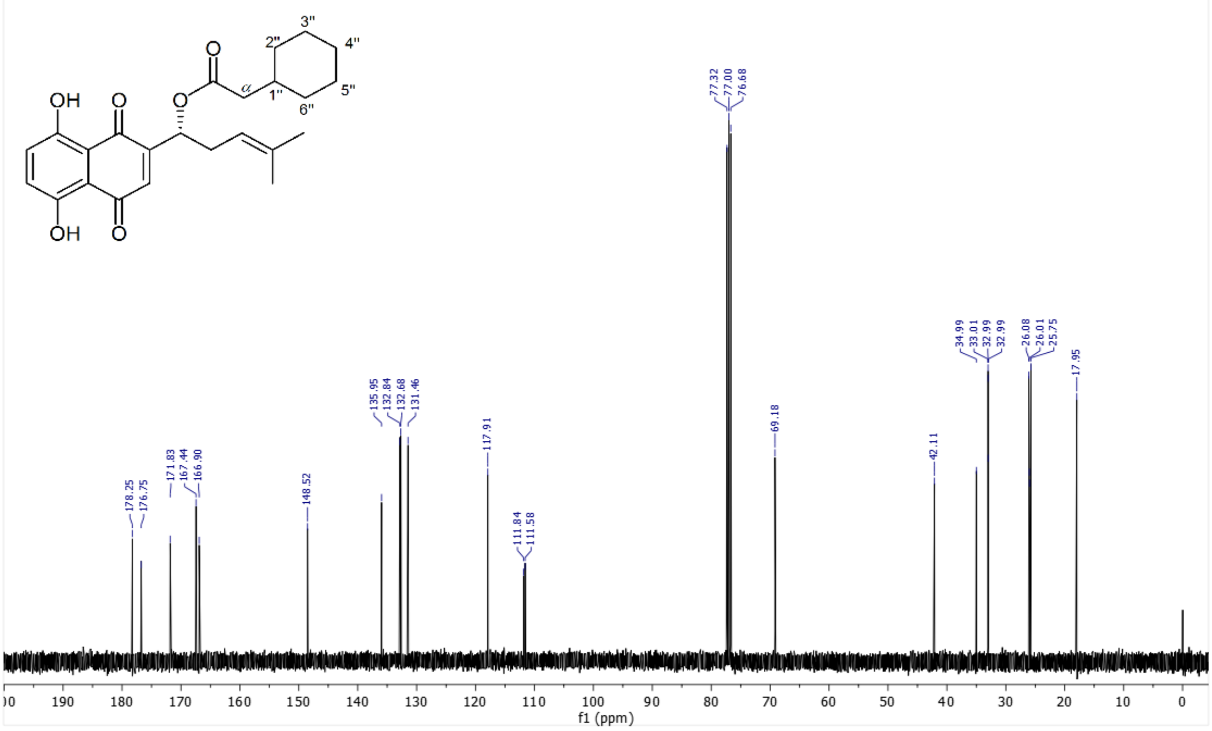

^{1}H-NMR spectrum of (*R*)-1-(1,4-dihydro-5,8-dihydroxy-1,4-dioxonaphthalen-2-yl)-4-methylpent-3-enyl 1-cyclohexen-1-ylacetate (**10**):

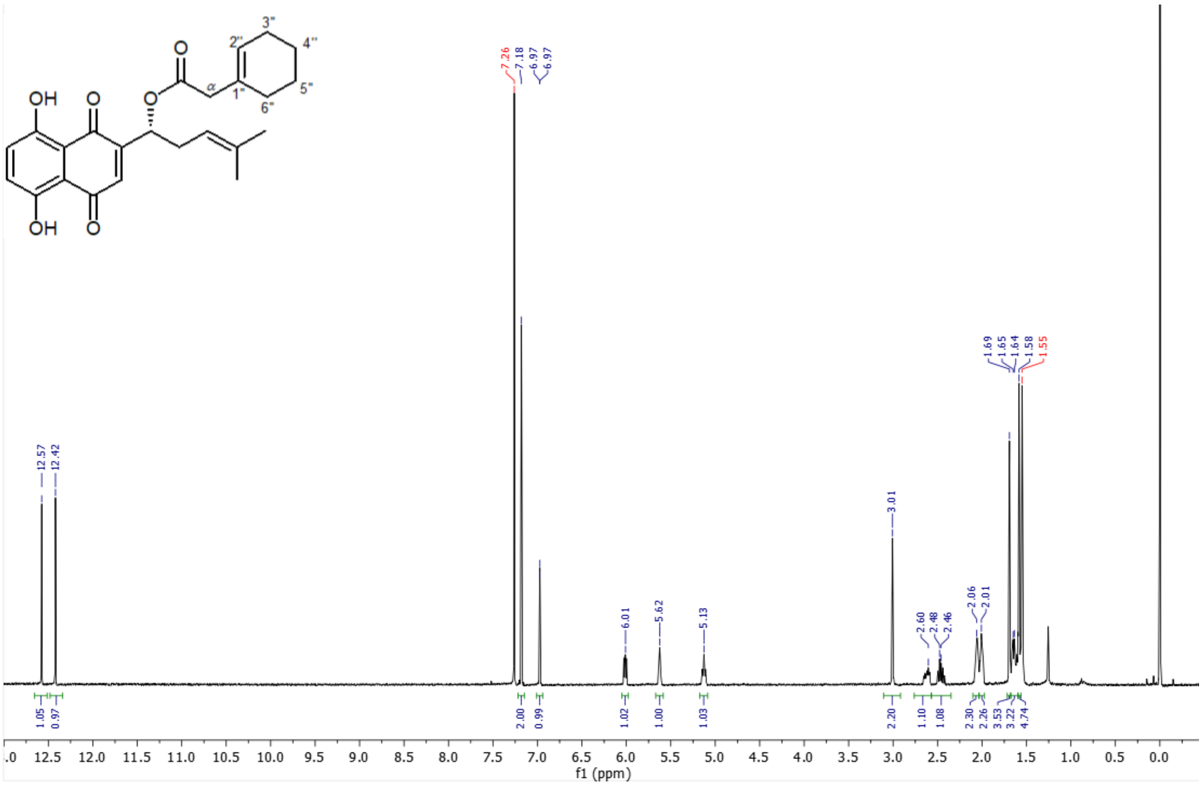

^{13}C-NMR spectrum of (*R*)-1-(1,4-dihydro-5,8-dihydroxy-1,4-dioxonaphthalen-2-yl)-4-methylpent-3-enyl 1-cyclohexen-1-ylacetate (**10**):

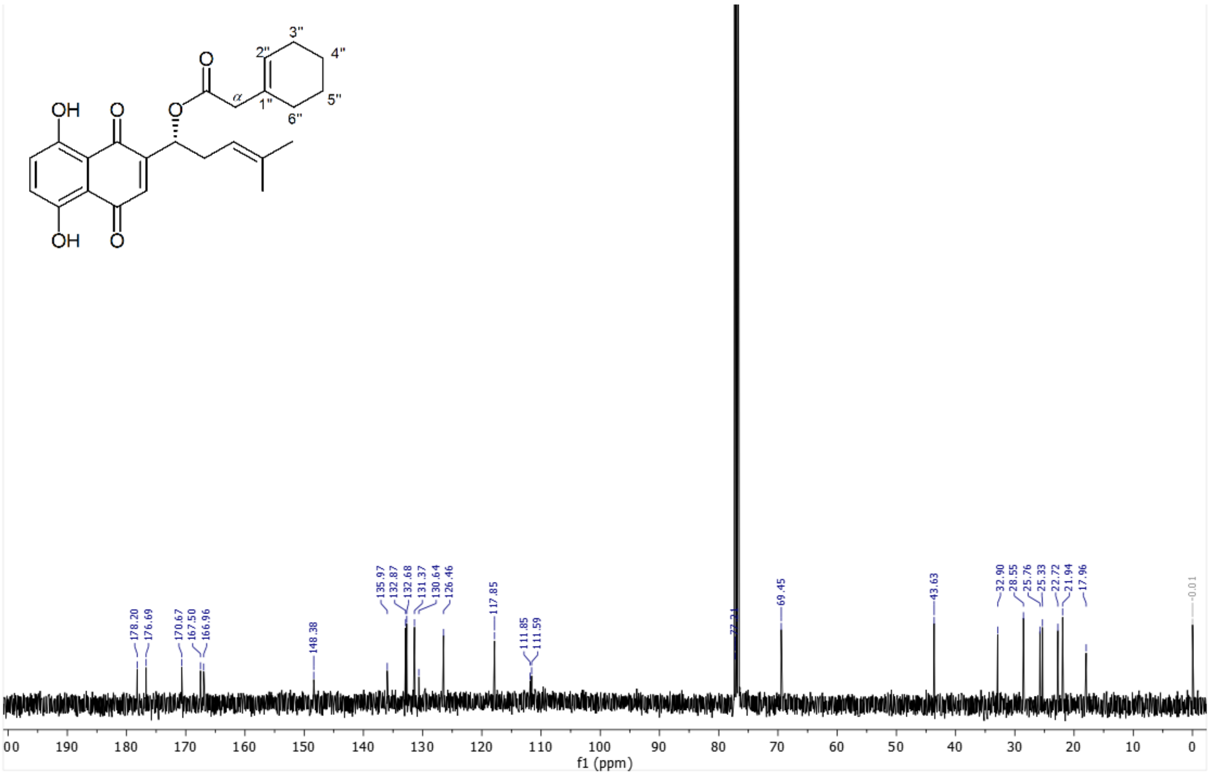

^{1}H-NMR spectrum of (*R*)-1-(1,4-dihydro-5,8-dihydroxy-1,4-dioxonaphthalen-2-yl)-4-methylpent-3-enyl cyclobutanecarboxylate (**12**):

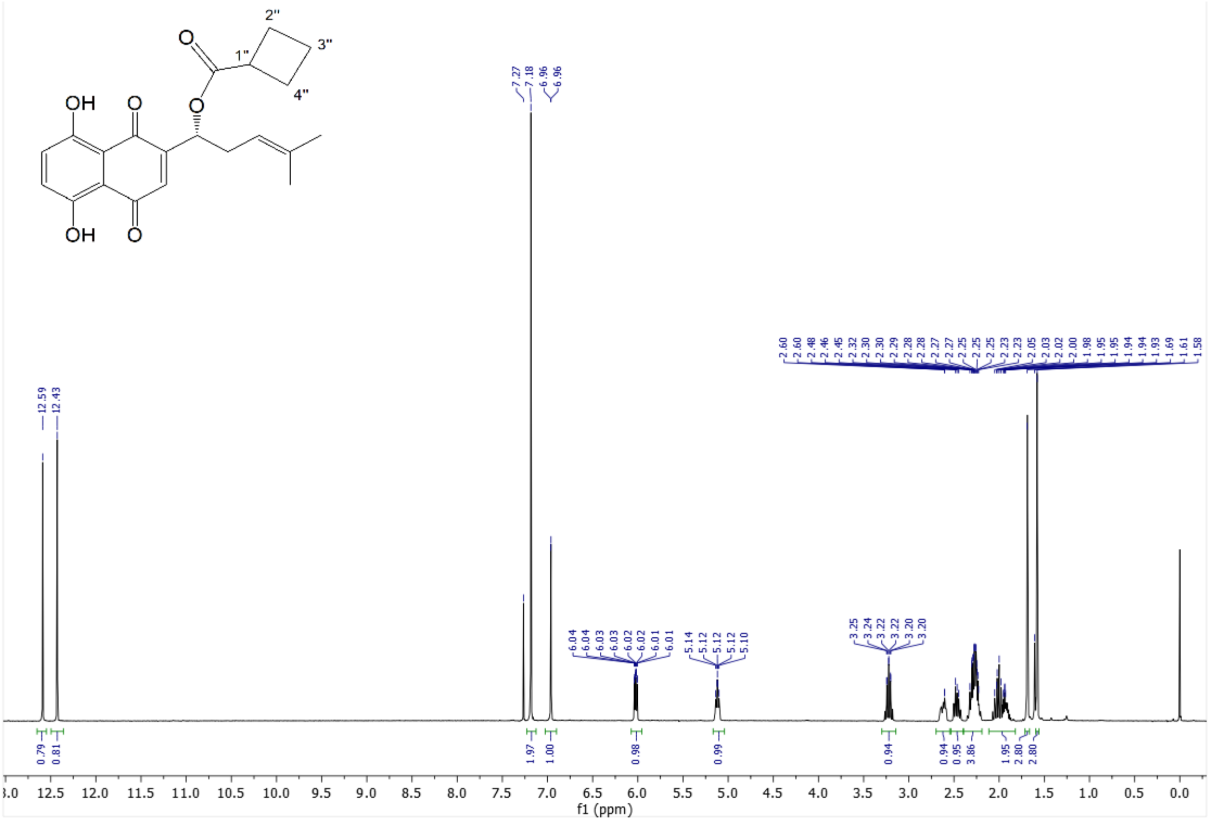

^{13}C-NMR spectrum of (*R*)-1-(1,4-dihydro-5,8-dihydroxy-1,4-dioxonaphthalen-2-yl)-4-methylpent-3-enyl cyclobutanecarboxylate (**12**):

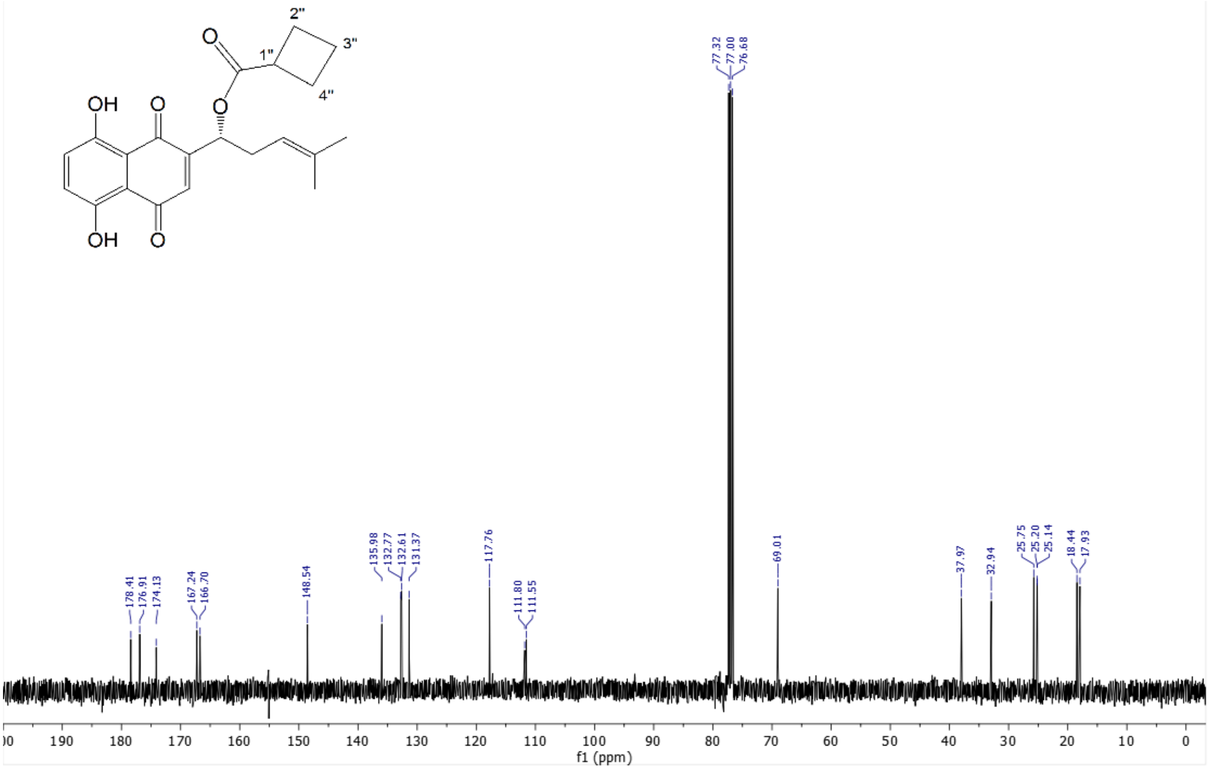

^{1}H-NMR spectrum of (*R*)-1-(1,4-dihydro-5,8-dihydroxy-1,4-dioxonaphthalen-2-yl)-4-methylpent-3-enyl cyclopentanecarboxylate (**13**):

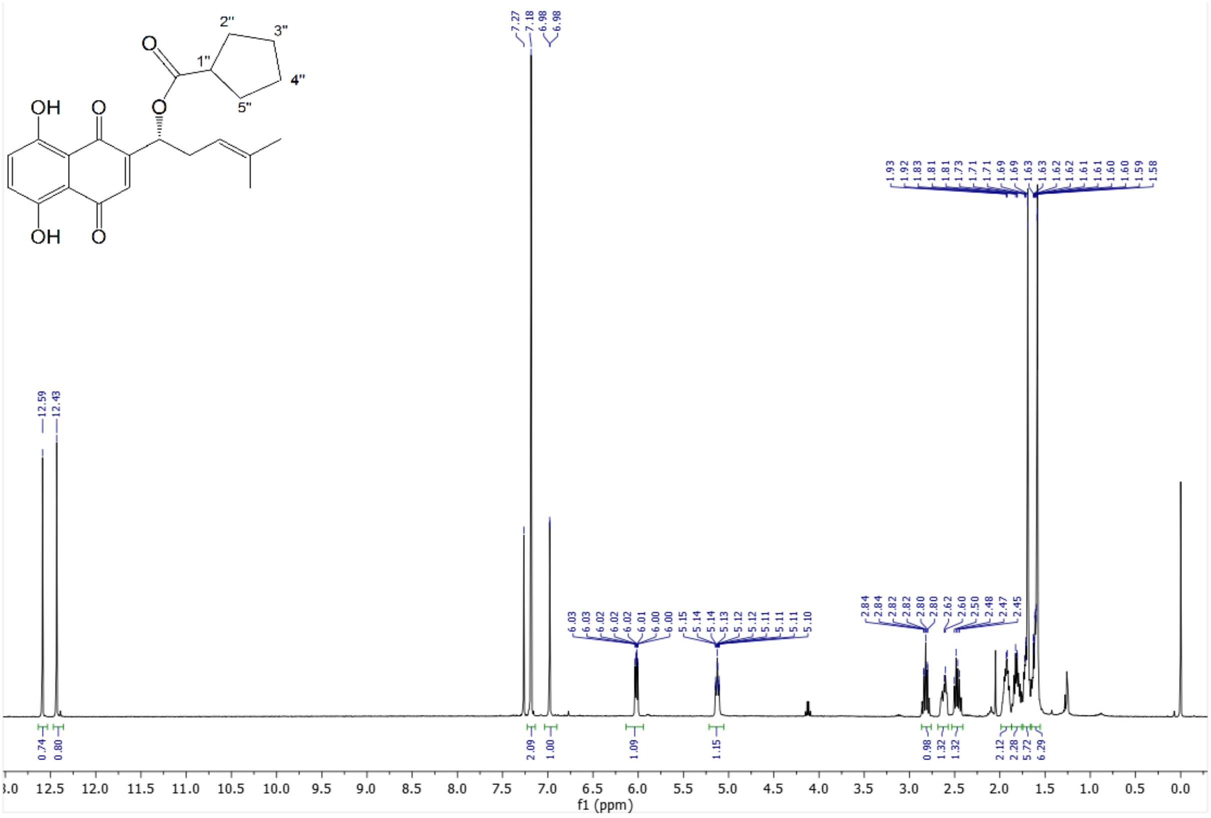

^{13}C-NMR spectrum of (*R*)-1-(1,4-dihydro-5,8-dihydroxy-1,4-dioxonaphthalen-2-yl)-4-methylpent-3-enyl cyclopentanecarboxylate (**13**):

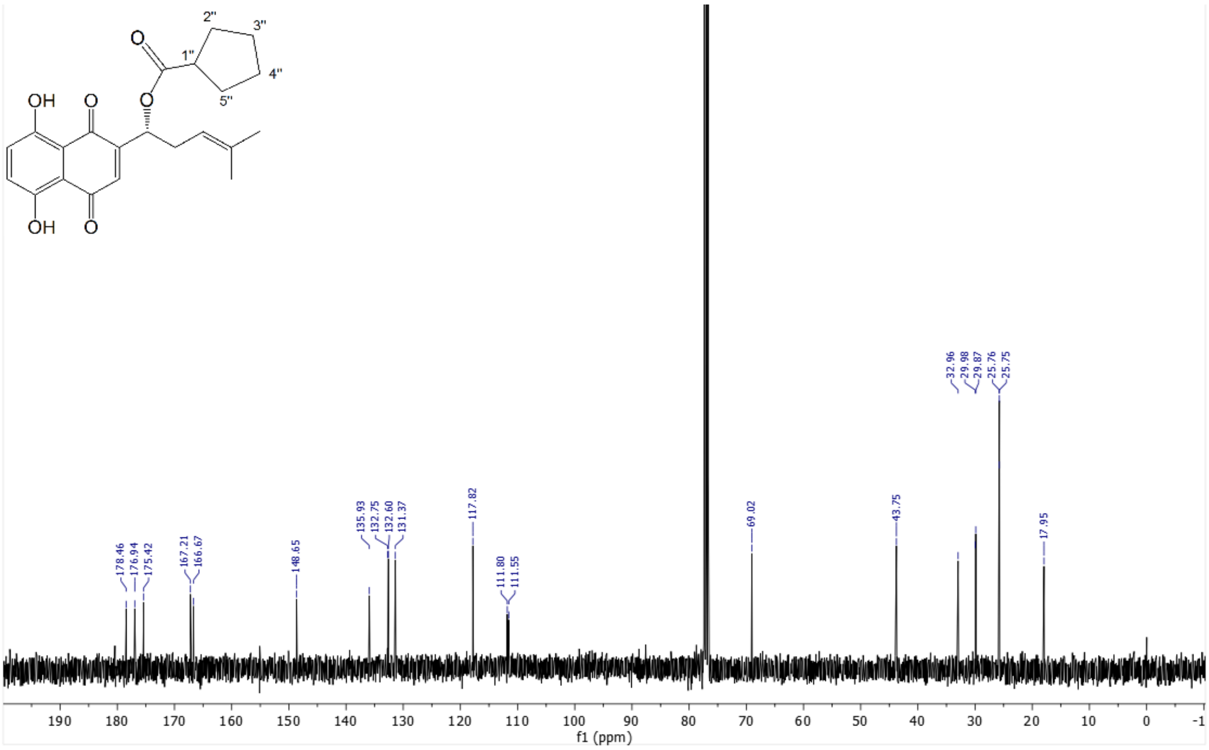

^{1}H-NMR spectrum of (*R*)-1-(1,4-dihydro-5,8-dihydroxy-1,4-dioxonaphthalen-2-yl)-4-methylpent-3-enyl cyclohexanecarboxylate (**14**):

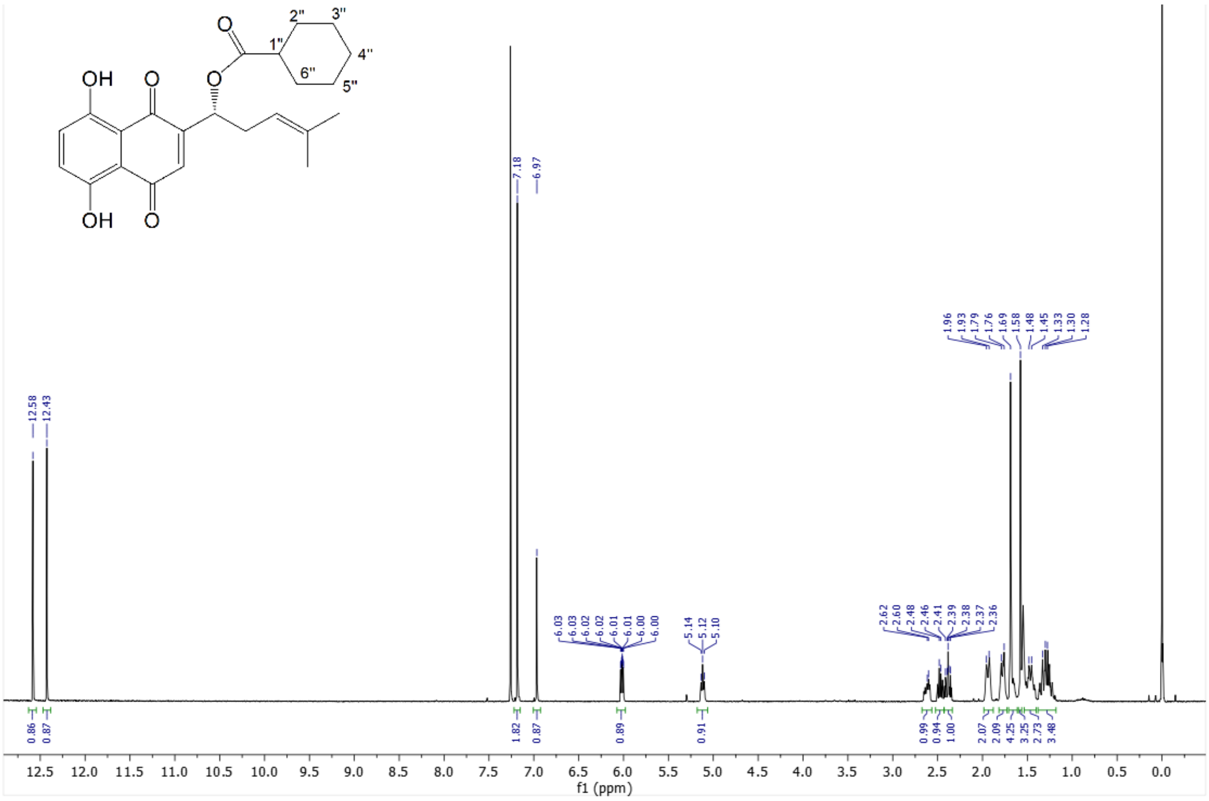

^{13}C-NMR spectrum of (*R*)-1-(1,4-dihydro-5,8-dihydroxy-1,4-dioxonaphthalen-2-yl)-4-methylpent-3-enyl cyclohexanecarboxylate (**14**):

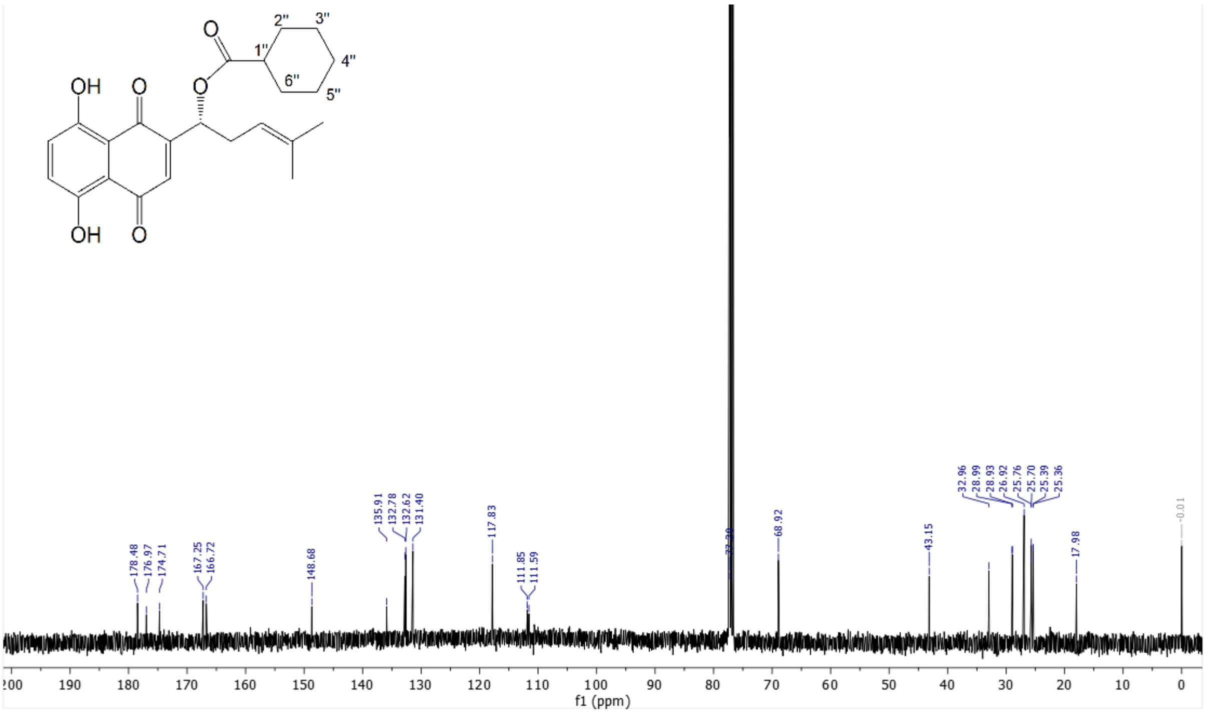

^{1}H-NMR spectrum of (*R*)-1-(1,4-dihydro-5,8-dihydroxy-1,4-dioxonaphthalen-2-yl)-4-methylpent-3-enyl cyclohex-1-enecarboxylate (**15**):

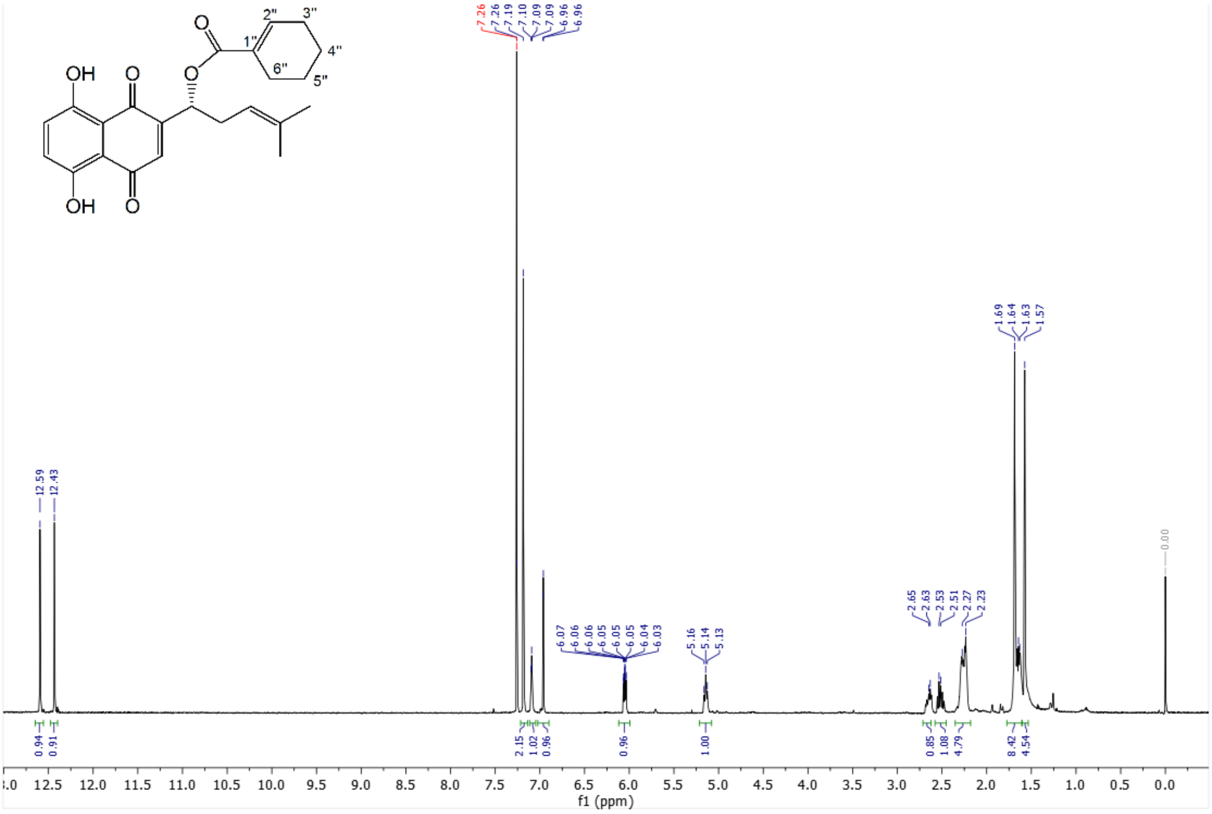

^{13}C-NMR spectrum of (*R*)-1-(1,4-dihydro-5,8-dihydroxy-1,4-dioxonaphthalen-2-yl)-4-methylpent-3-enyl cyclohex-1-enecarboxylate (**15**):

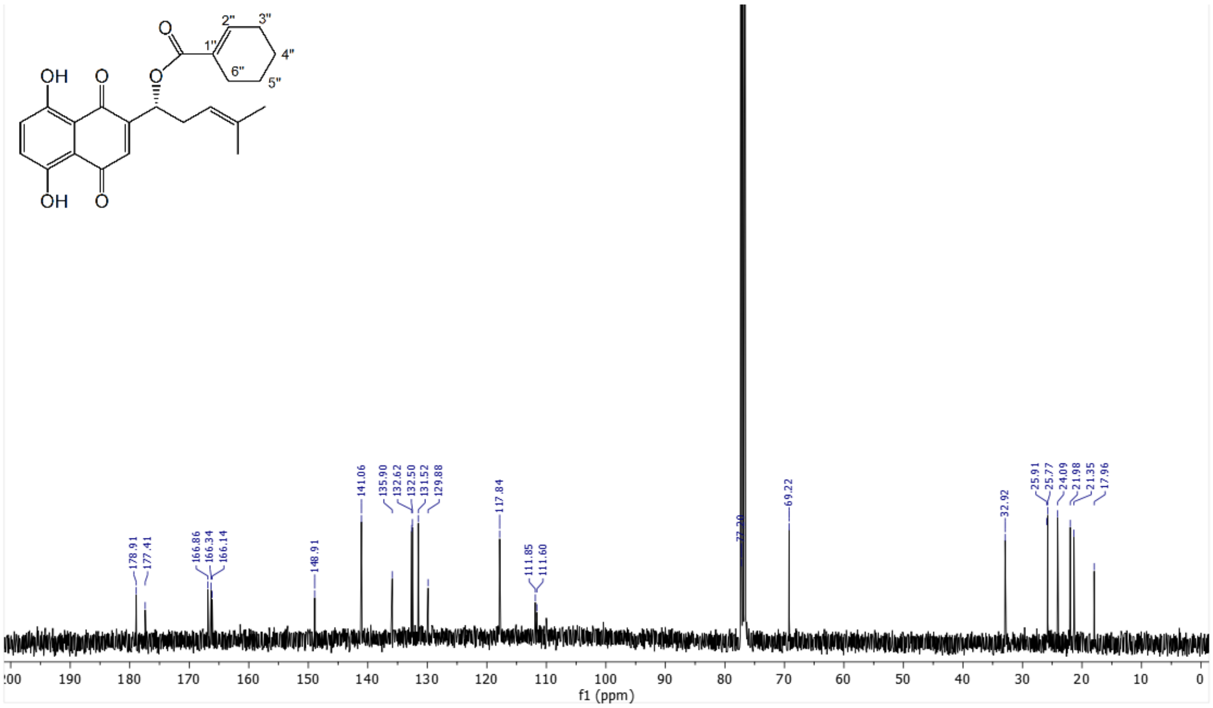

^{1}H-NMR spectrum of (*R*)-1-(1,4-dihydro-5,8-dihydroxy-1,4-dioxonaphthalen-2-yl)-4-methylpent-3-enyl cyclohex-3-enecarboxylate (**16**):

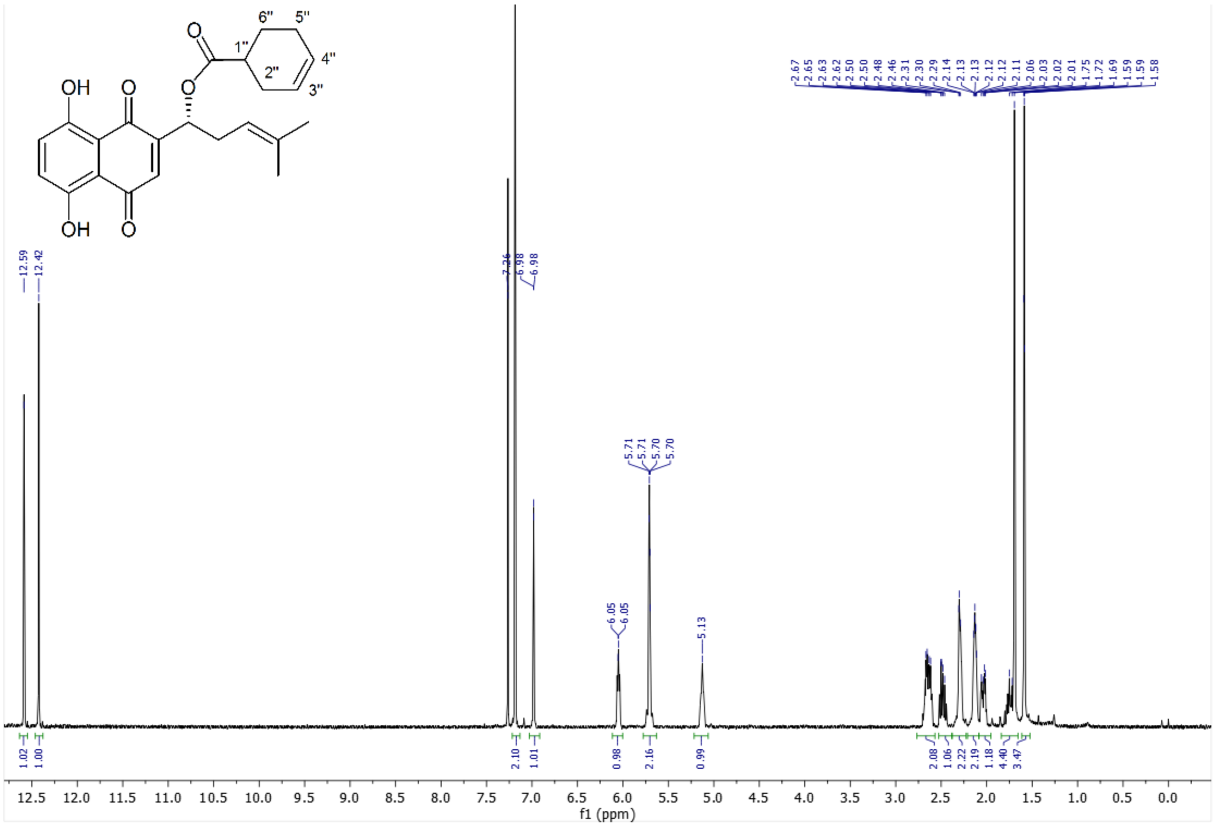

^{13}C-NMR spectrum of (*R*)-1-(1,4-dihydro-5,8-dihydroxy-1,4-dioxonaphthalen-2-yl)-4-methylpent-3-enyl cyclohex-3-enecarboxylate (**16**):

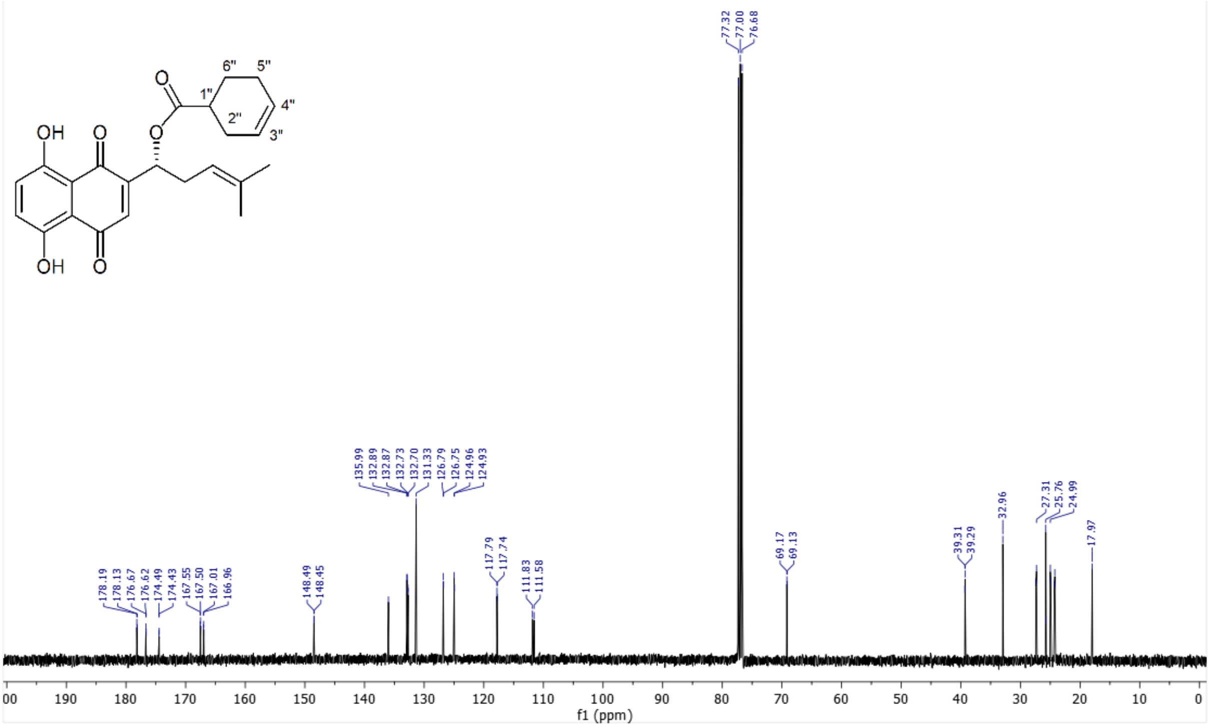

^{1}H-NMR spectrum of (*R*)-1-(1,4-dihydro-5,8-dihydroxy-1,4-dioxonaphthalen-2-yl)-4-methylpent-3-enyl trans 2-methylcyclopropanecarboxylate (**17**):

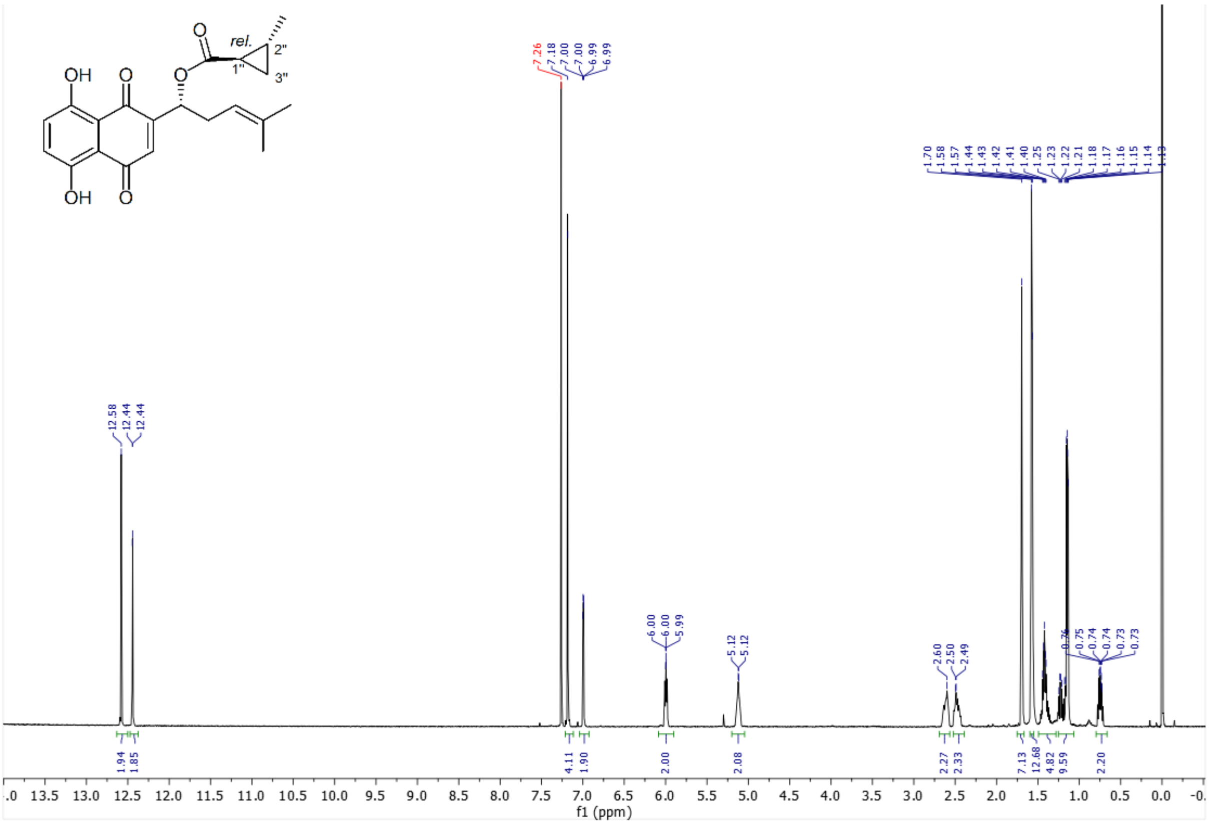

^{13}C-NMR spectrum of (*R*)-1-(1,4-dihydro-5,8-dihydroxy-1,4-dioxonaphthalen-2-yl)-4-methylpent-3-enyl cyclohex-3-enecarboxylate (**17**):

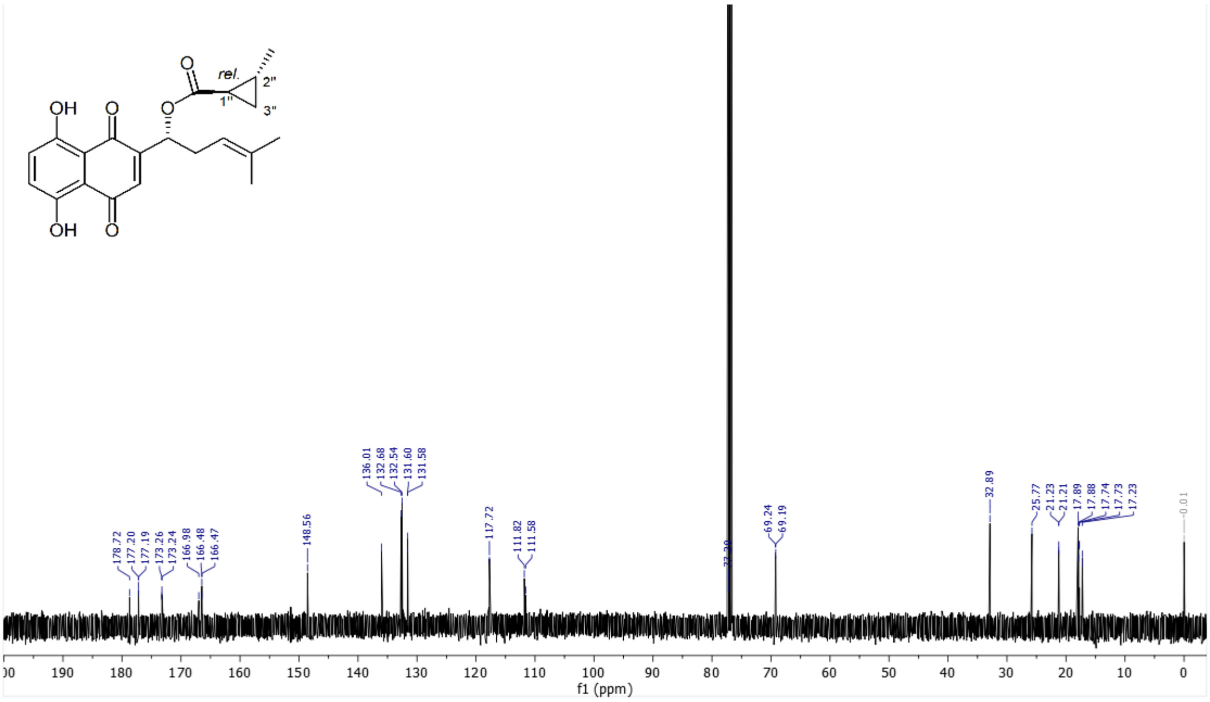

^{1}H-NMR spectrum of Exo-(*R*)-1-(1,4-dihydro-5,8-dihydroxy-1,4-dioxonaphthalen-3-yl)-4-methylpent-3-enyl bicyclo[2.2.1]hept-5-ene-2-carboxylate (**18**):

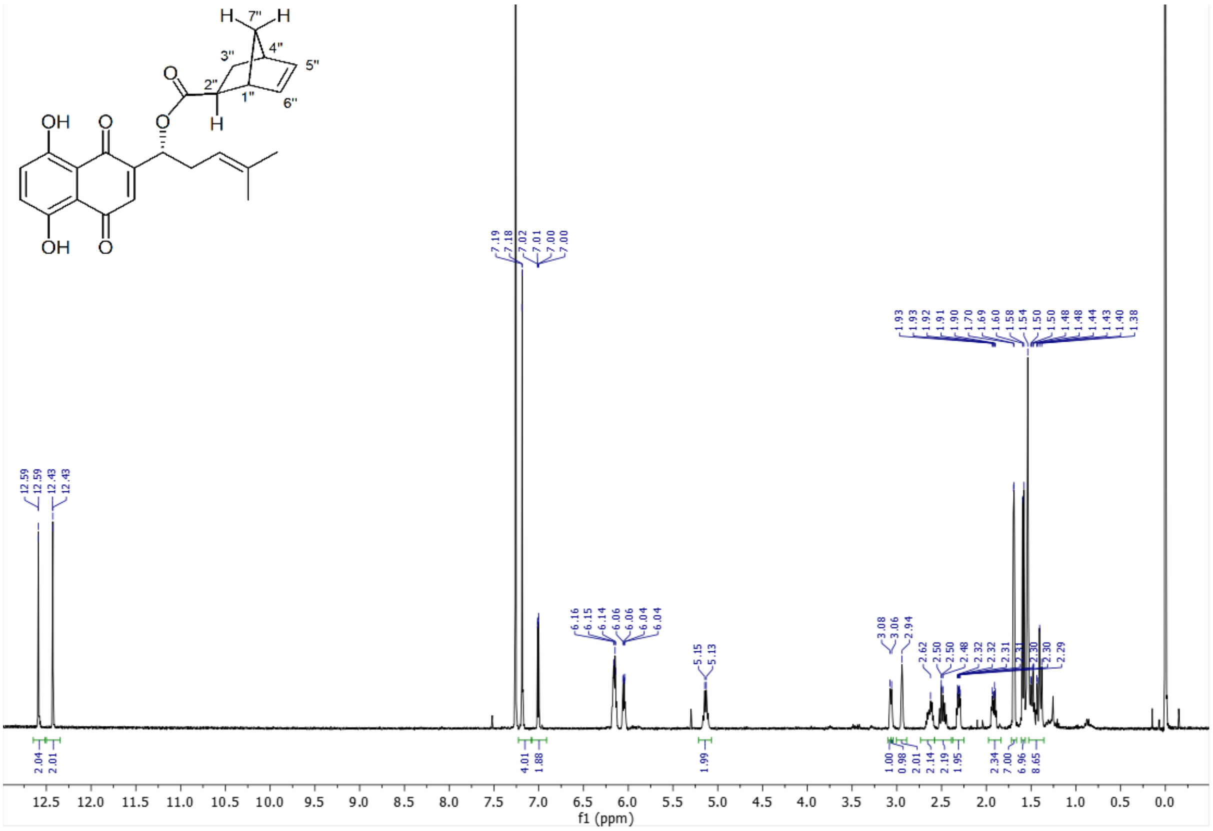

^{13}C-NMR spectrum of Exo-(*R*)-1-(1,4-dihydro-5,8-dihydroxy-1,4-dioxonaphthalen-2-yl)-4-methylpent-3-enyl cyclohex-3-enecarboxylate (**18**):

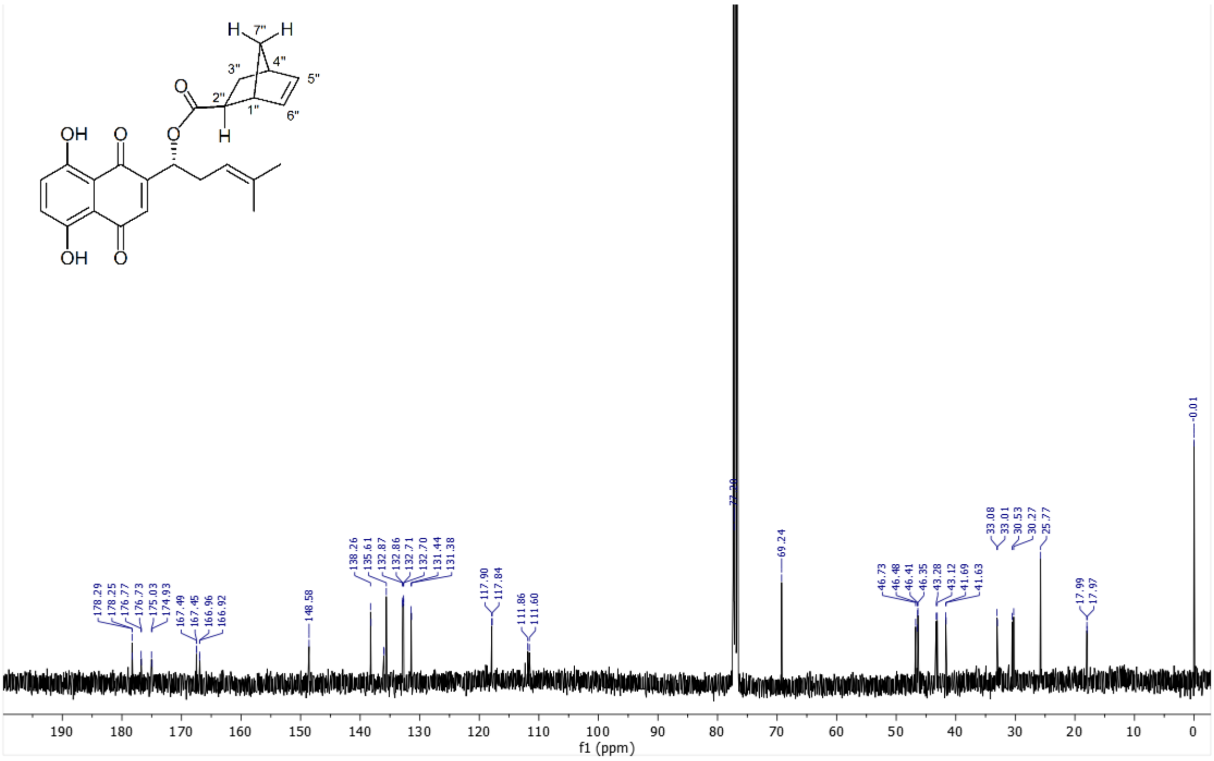

^{1}H-NMR spectrum of Endo-(*R*)-1-(1,4-dihydro-5,8-dihydroxy-1,4-dioxonaphthalen-3-yl)-4-methylpent-3-enyl bicyclo[2.2.1]hept-5-ene-2-carboxylate (**19**):

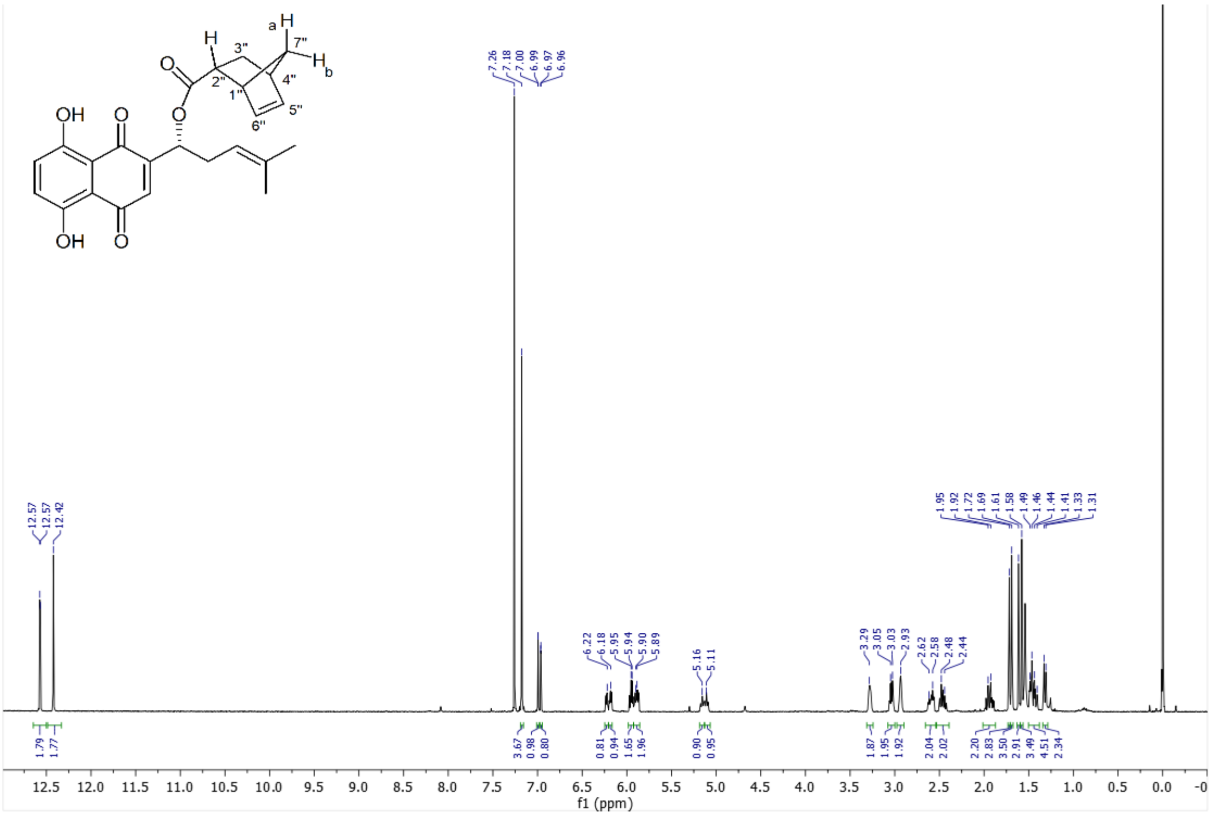

^{13}C-NMR spectrum of Endo-(*R*)-1-(1,4-dihydro-5,8-dihydroxy-1,4-dioxonaphthalen-2-yl)-4-methylpent-3-enyl cyclohex-3-enecarboxylate (**19**):

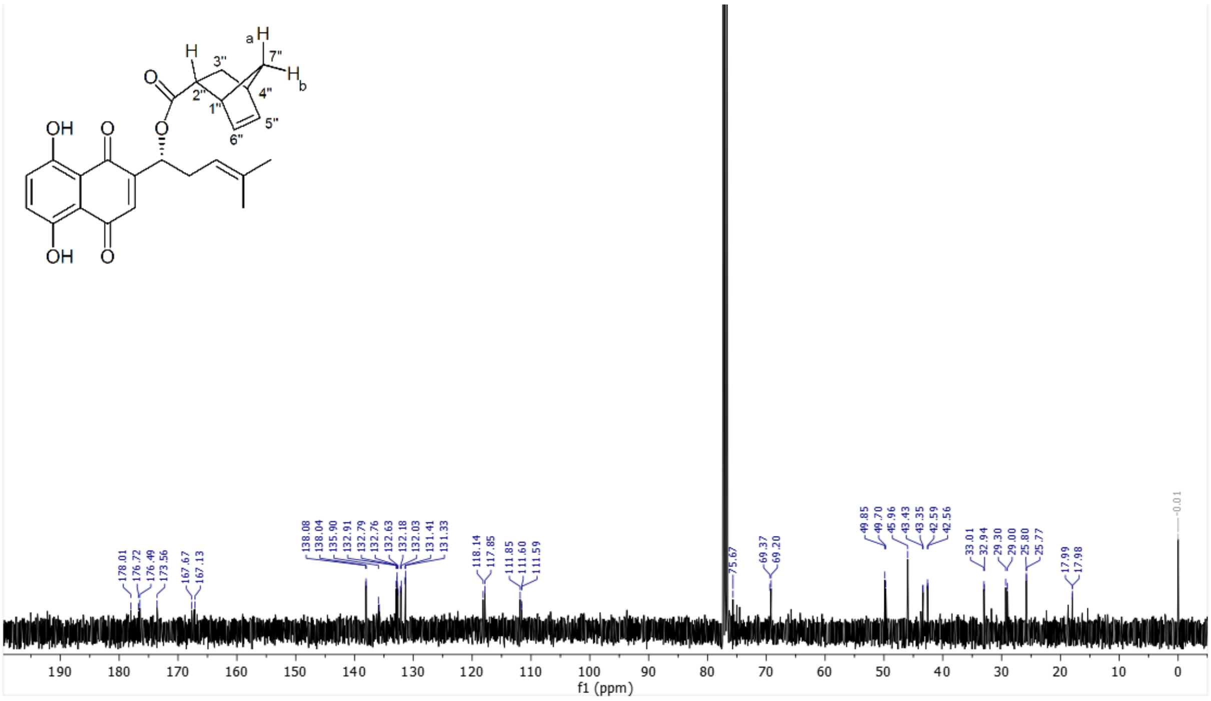

HMBC spectrum of Endo-(*R*)-1-(1,4-dihydro-5,8-dihydroxy-1,4-dioxonaphthalen-2-yl)-4-methylpent-3-enyl cyclohex-3-enecarboxylate (**19**):

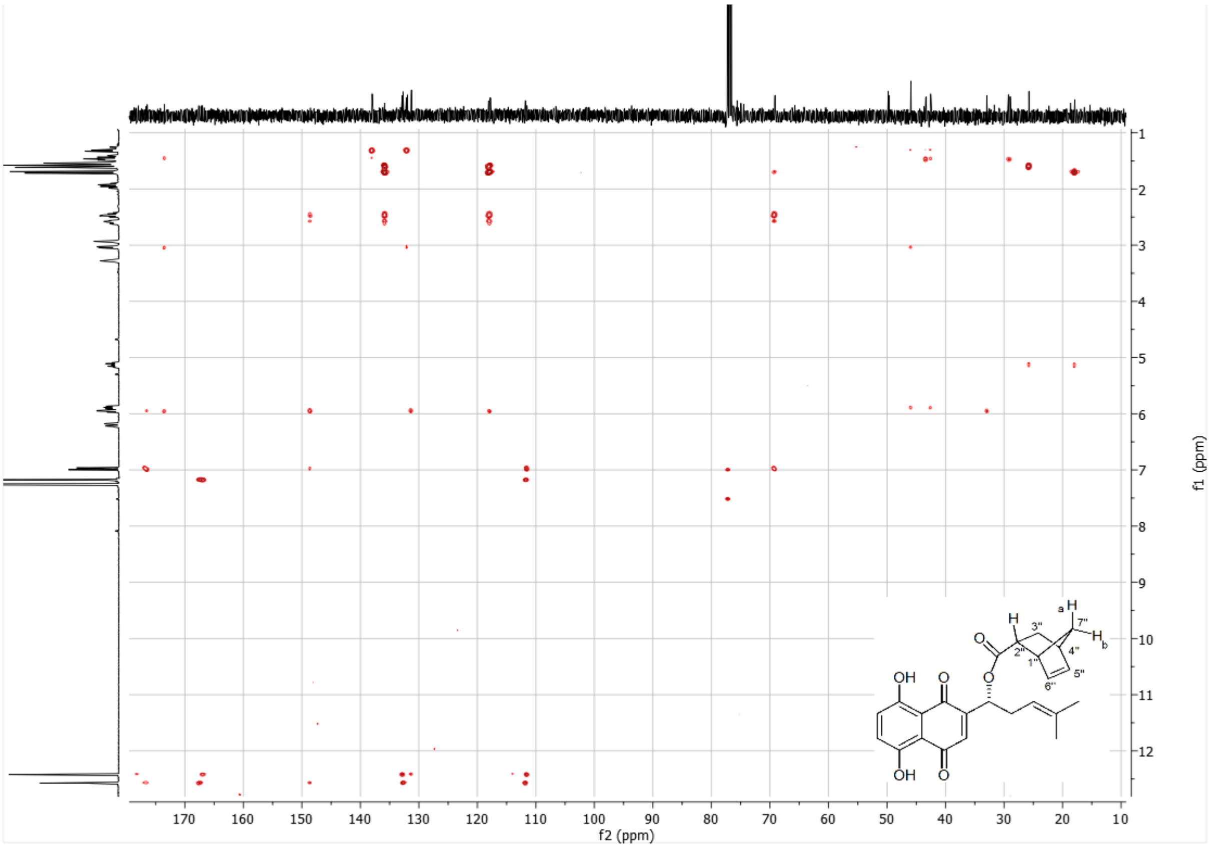

^{1}H-NMR spectrum of (*R*)-1-(1,4-dihydro-5,8-dihydroxy-1,4-dioxonaphthalen-2-yl)-4-methylpent-3-enyl bicyclo[2.2.1]heptane-2-ylacetate (**20**):

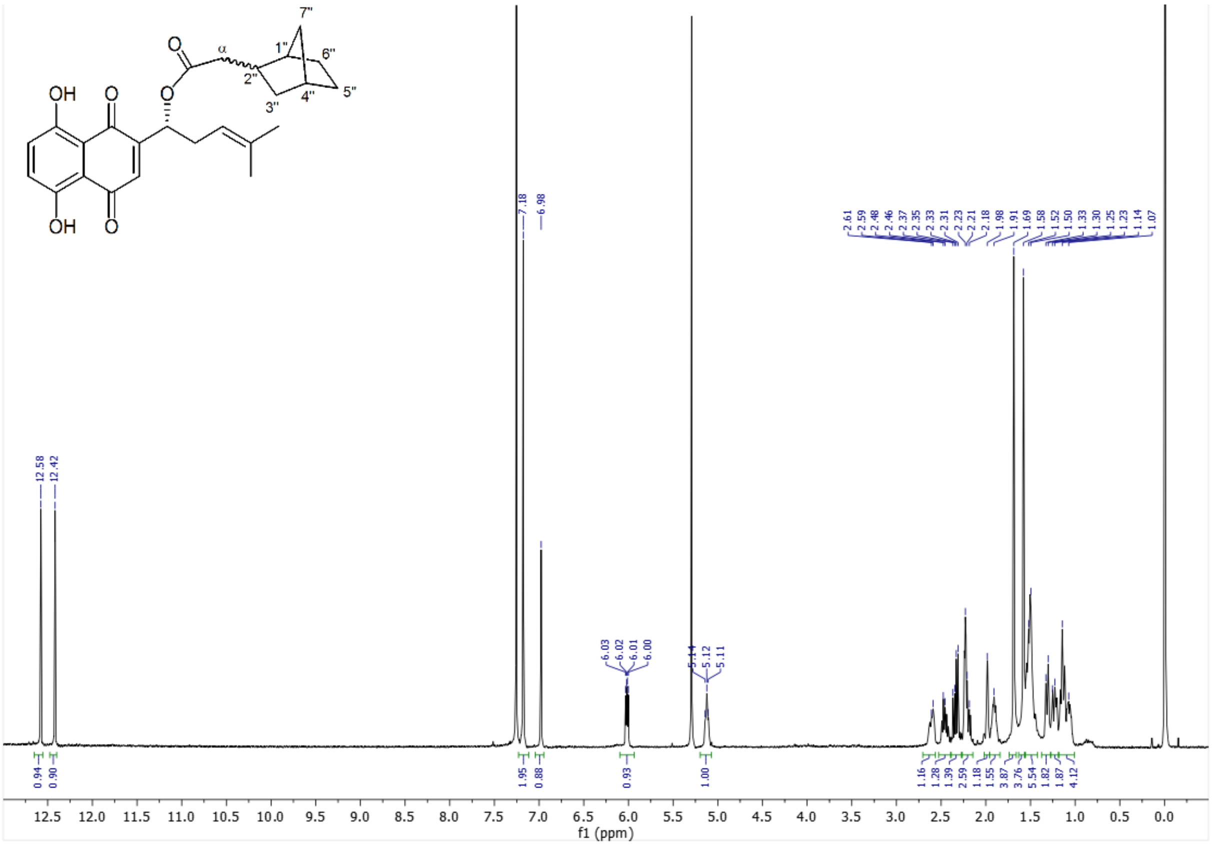

^{13}C-NMR spectrum of (*R*)-1-(1,4-dihydro-5,8-dihydroxy-1,4-dioxonaphthalen-2-yl)-4-methylpent-3-enyl bicyclo[2.2.1]heptane-2-ylacetate (**20**):

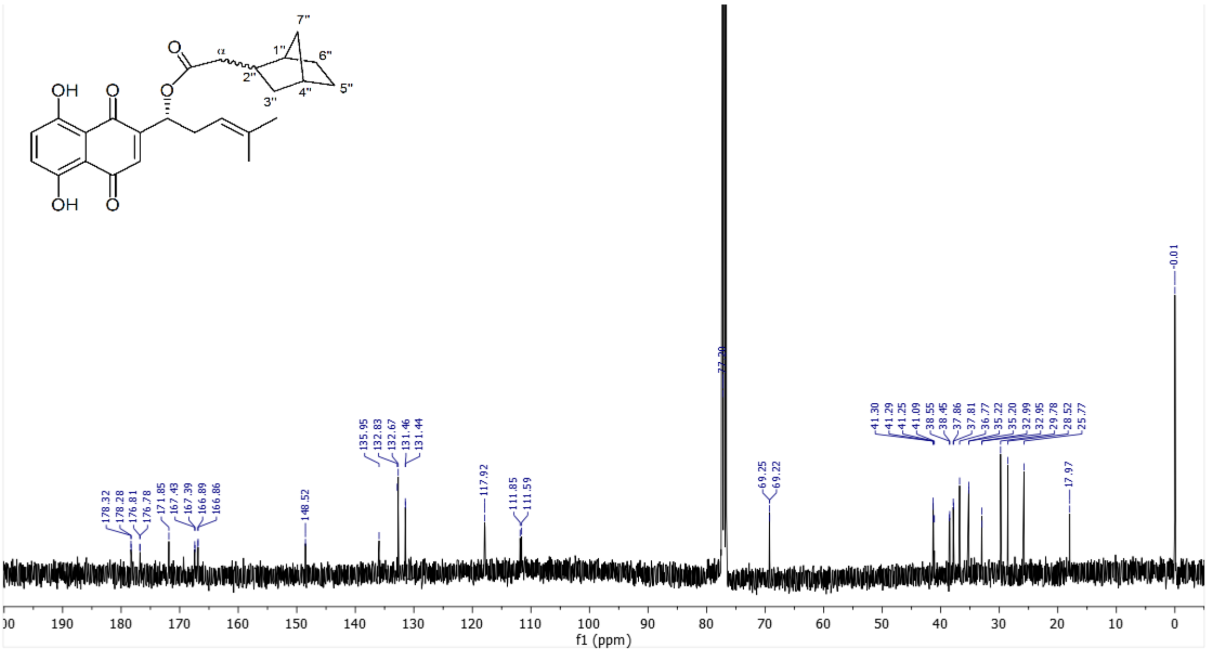

207 HMBC spectrum of (*R*)-1-(1,4-dihydro-5,8-dihydroxy-1,4-dioxonaphthalen-2-yl)-4-methylpent-3-enyl
208 bicyclo[2.2.1]heptane-2-acetate (**20**):

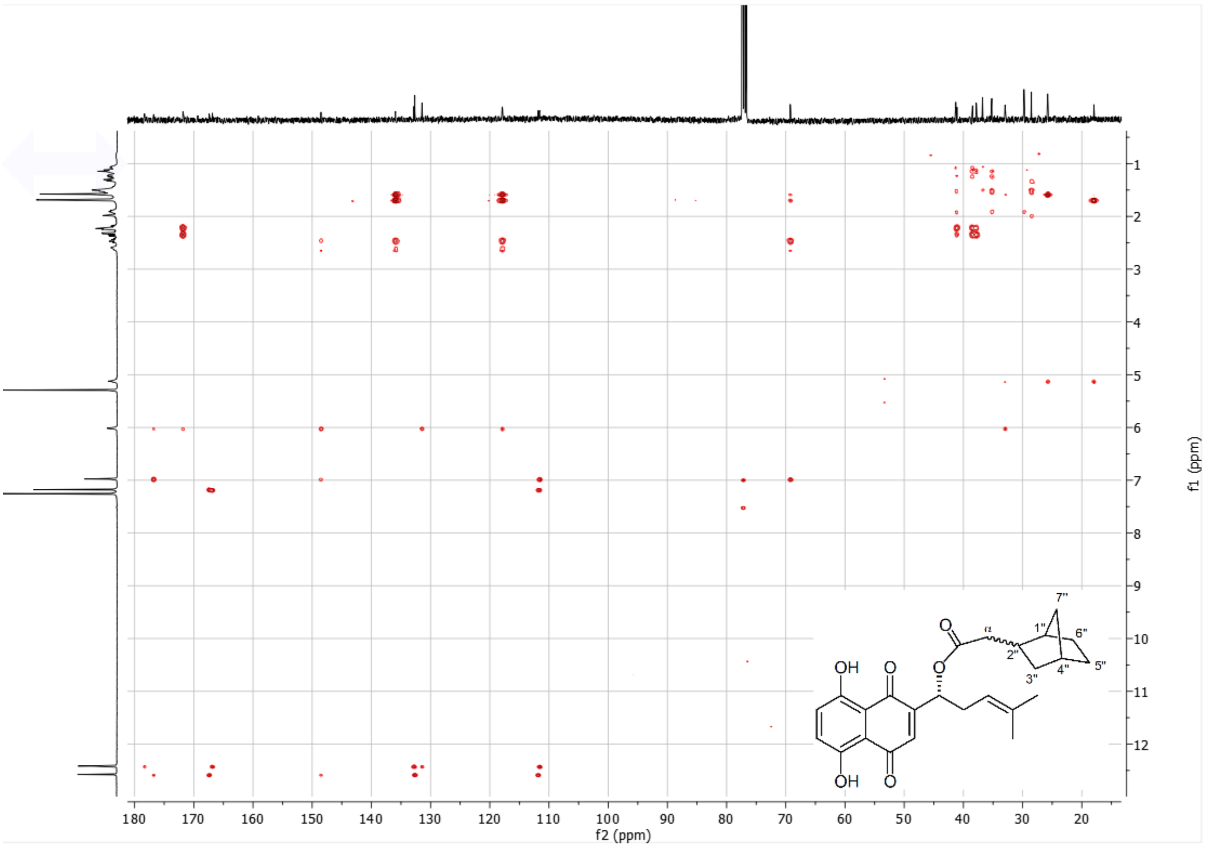

209
210

3. Syntheses of cycloalkylideneacids 2a to 5a and cyclobutylacetic acid (7a)

*Cyclobutylideneacetic acid (**2a**)*

At 0 °C triethylphosphonoacetate (10.2 mL, 11.5 g, 51.4 mmol) was added to a suspension NaH (60% in mineral oil; 2.05 g, 66.8 mmol) in abs. Et_2O (120 mL). After stirring for 5 min, a solution of cyclobutanone (3.74 mL, 3.50 g, 50 mmol) in abs. Et_2O (10 mL) was added. After 4 h at room temperature, water (100 mL) was added. The organic layer was separated and the aqueous layer was extracted with Et_2O (3 x 50 mL). Combined organic layers were dried over anhydrous Na_2SO_4 and evaporated resulting in 6.3 g raw ester (contained ca. 20 % ethyl cyclopentenylacetate) which was used for the next reaction without further purification.

A solution of LiOH · H_2O (4.20 g, 100 mmol) in water (25 mL) was added to a solution of raw ethyl cyclobutylidenacetat (1.40 g, 10 mmol) in THF (25 mL). The mixture was stirred for 18 h at room temperature. TLC showed very little conversion. THF, water and MeOH (10 mL each), were added and the mixture was stirred for further three days. No starting material was visible in TLC. The mixture was concentrated under reduced pressure to about 30 mL, washed with ether (2 x 10 mL), acidified with conc. HCl (ca. 8 mL) and extracted with Et_2O (3 x 10 mL). This extract was dried over anhydrous Na_2SO_4 and evaporated. 580 mg of the raw product (0.99 g) were purified by CC (silica, cyclohexane / EtOAc = 3:1 to 2:1) resulting in 352 mg cyclobutylideneacetic acid (**2a**) (54%).

^{1}H-NMR ($CDCl_3$): δ 2.11 (quint, *J* = 8.0 Hz, 2H, $CH_2CH_2CH_2$), 2.86 (tm, *J* = 7.9 Hz, 2H, trans CH_2-C=), 3.14 (tm, *J* = 8.1 Hz, 2H, cis CH_2-C=), 5.59 (quint, *J* = 2.3 Hz, 1H, =CH), 11.05 (s, br, 1H, COOH); ^{13}C-NMR ($CDCl_3$): δ 17.5 ($CH_2CH_2CH_2$), 32.5, 34.1 (2 x CH_2-C=), 112.0 (=CH), 171.4, 172.3 (C=CH-CO).

*Cyclopentylideneacetic acid (**3a**) and 2-cyclopentenyl acetic acid (**3b**)*

At 0 °C triethylphosphonoacetate (10.2 mL, 11.5 g, 51.4 mmol) was added to a suspension NaH (60% in mineral oil; 2.05 g, 66.8 mmol) in abs. Et_2O (120 mL). After stirring for 5 min, a solution of cyclopentanone (4.43 mL, 4.21 g, 50 mmol) in abs. Et_2O (10 mL) was added. After 4 h (stirrer stuck after ca. 1 h) at room temperature, water (100 mL) was added. The organic layer was separated and the aqueous layer was extracted with Et_2O (3 x 50 mL). Combined organic layers were dried over anhydrous Na_2SO_4 and evaporated resulting in 8.1 g raw ester (ethyl cyclopentylideneacetate with ca. 20 % ethyl 2-cyclopentenylacetate) which was used for the next reaction without further purification.

0.22 g (5.2 mmol) LiOH · H_2O in 20 mL water was added to a solution of 0.77 g (5 mmol) raw ethyl cyclopentylidenacetate in THF (25 mL) and MeOH (10 mL). After stirring for 2 h at 50 °C (bath temperature), the mixture was concentrated under reduced pressure to approx. 25 mL, washed with ether (3 x 10 mL), acidified with conc. HCl (ca. 0.4 mL) and extracted with ether (3 x 10 mL). The combined extracts were dried over Na_2SO_4 and concentrated under reduced pressure. The residue consisted in 500 mg of a mixture of **3a** and **3b** (1 : 3; 79 %).

The raw product was submitted to CC (silica, cyclohexane / EtOAc = 3 : 1 to 2 : 1) resulting in 66 mg **3a** (10 %), **3b** 100 mg (16 %) and 66 mg of an isomeric mixture (10 %).

Cyclopentylideneacetic acid (**3a**): ^{1}H-NMR ($CDCl_3$): δ 1.63-1.80 (m, 4H, $CH_2CH_2CH_2CH_2$), 2.47 (tm, *J* = 7.1 Hz, 2H, trans CH2-C=), 2.78 (tm, *J* = 7.1 Hz, 2H, cis CH2-C=), 5.83 (s, 1H, =CH), 11.63 (s, br, 1H, COOH); ^{13}C-NMR ($CDCl_3$): δ 25.4, 26.3 ($CH_2CH_2CH_2CH_2$), 33.0 (cis CH2-C=), 36.3 (trans CH_2-C=), 111.1 (=CH), 172.6, 173.0 (C=CH-COO).

2-Cyclopentenylacetic acid (**3b**): ^{1}H-NMR ($CDCl_3$): δ 1.91 (quint, *J* = 7.5 Hz, 2H, H-4), 2.35 (t, *J* = 7.5 Hz, 4H, H-3 and H-5), 3.17 (s, 2H, CH_2-α), 5.59 (sm, 1H, =CH), 11.25 (s, vbr, 1H, COOH); ^{13}C-NMR (CDCl3): δ 23.4, (C-4), 32.5 (C-α), 35.0, 36.7 (C-3 and C-5), 128.9 (C-2), 135.8 (C-1), 178.2 (COO).

*Cyclohexylideneacetic acid (**4a**)*

Diethylphosphit (1.29 mL, 1.38 mg, 10 mmol) was added to a suspension of NaH (60 % in mineral oil; 1.20 g, 30 mmol) in abs. glyme (32 mL). After stirring for 10 min, a solution of chloroacetic acid (945 mg, 10 mmol) in abs. glyme (10 mL) was added and stirred for 40 min till gas evolution had ceased. Cyclohexanone (1.03 mL, 980 mg, 10 mmol) was added. After stirring for further 90 min and addition of EtOH (1.7 mL), the mixture was poured into water (160 mL). Washing with Et_2O (4 x 50 mL), acidification with conc. HCl (ca. 3 mL), extraction with Et_2O (4 x 50 mL), drying over anhydrous Na_2SO_4 and evaporation resulted in 707 mg raw product. 668 mg of this material was submitted to CC (silica, cyclohexane / EtOAc = 3:1) resulting in 261 mg cyclohexylideneacetic acid (**4a**) (20 %).

^{1}H-NMR ($CDCl_3$): δ 1.56-1.71 (m, 6H, 3 x CH_2), 2.22 (t, *J* = 6.0 Hz, 2H, trans CH_2-C=), 2.83 (t, *J* = 5.8 Hz, 2H, cis CH2-C=), 5.70 (quint, *J* = 1.2 Hz, 1H, =CH), 11.15 (s, br, 1H, COOH); ^{13}C-NMR ($CDCl_3$): δ 26.1, 27.8, 28.6 (3 x CH_2), 30.1 (cis CH_2-C=), 38.3 (trans CH_2-C=), 115.2 (=CH), 167.5 (C=CH-CO), 172.6 (CO).

*Cycloheptylideneacetic acid (**5a**)*

Diethylphosphit (0.78 mL, 872 mg, 6.31 mmol) was added to a suspension of NaH (60 % in mineral oil; 1.10 g, 27.5 mmol) in abs. glyme (20 mL). After stirring for 10 min, a solution of chloroacetic acid (570 mg, 6.03 mmol) in abs. glyme (7 mL) was added and stirred for 30 min till gas evolution had ceased. Cycloheptanone (0.71 mL, 677 mg, 6.03 mmol) was added. After stirring for further 3 h and addition of EtOH (1 mL), the mixture was poured into water (100 mL). Washing with Et_2O (2 x 50 mL), acidification with conc. HCl to pH=4, extraction with Et_2O (4 x 30 mL), drying over anhydrous Na_2SO_4 and evaporation resulted in 360 mg raw product. 300 mg of this material was submitted to CC (silica, cyclohexane / EtOAc = 3:1 to 2:1) resulting in 85 mg cycloheptylideneacetic acid (**5a**) (11 %).

^{1}H-NMR ($CDCl_3$): δ 1.50-1.58 (m, 4H, $CH_2CH_2CH_2CH_2$), 1.62-1.72 (m, 4H, $CH_2CH_2CH_2CH_2$), 2.40 (ddd, *J* = 6.0, ~4.5, 0.6 Hz, 2H, trans CH2-C=), 2.88 (ddd, *J* = 6.2, ~5, 1.2 Hz, 2H, cis CH2-C=), 5.63 (s, 1H, =CH), 11.7 (s, br, 1H, COOH); ^{13}C-NMR ($CDCl_3$): δ 26.4, 27.9, 28.9, 29.7 (4 x CH2), 32.3(cis CH_2-C=), 39.2 (trans CH_2-C=), 115.2 (=CH), 169.9 (C=CH-CO), 172.0 (CO).

*Cyclobutylacetic acid (**7a**)*

Blank magnesium chips (370 mg, 15.2 mmol) were covered with abs. THF (ca. 0.5 mL). Two drops (bromomethyl)cyclobutane (from 2.09 g, 14.0 mmol) were added. As the reaction started, the rest of the bromide was solved in abs. THF (4 mL) and the solution was added dropwise with stirring. After addition, the mixture was allowed to stand for 18 h at room temperature. The mixture was diluted abs. THF (10 mL), warmed near to reflux und slowly poured on crushed dry ice (100 mL). After warming up to about 0 °C and addition of EtOAc (10 mL), the mixture was washed with 2 M HCl (10 mL) and saturated with NaCl. The organic layer was separated and the aqueous layer was extracted with EtOAc (10 mL), the combined organic layers were dried over Na_2SO_4 and concentrated under reduced pressure resulting in 1.02 g cyclobutylacetic acid (**7a**) (64 %).

^{1}H-NMR ($CDCl_3$): δ 1.66-1.77 (m, 2H, trans, $CH_2CH_2CH_2$), 1.79-1.95 (m, 2H, $CH_2CH_2CH_2$), 2.10-2.19 (m, 2H, cis, $CH_2CH_2CH_2$), 2.44 (d, *J* = 7.5 Hz, 2H, CH_2CO), 2.68 (sept., *J* = 7.8 Hz, 1H, CH), 11.08 (s, br, 1H, COOH); ^{13}C-NMR ($CDCl_3$): δ 18.4 ($CH_2CH_2CH_2$), 28.1 ($CH_2CH_2CH_2$), 31.9 (CH), 40.9 (C-α), 179.5 (COO).

4. ^{1}H NMR-spectra of synthesized acids (400 MHz; $CDCl_3$) TMS as internal standard

^{1}H-NMR spectrum of cyclobutylideneacetic acid (**2a**):

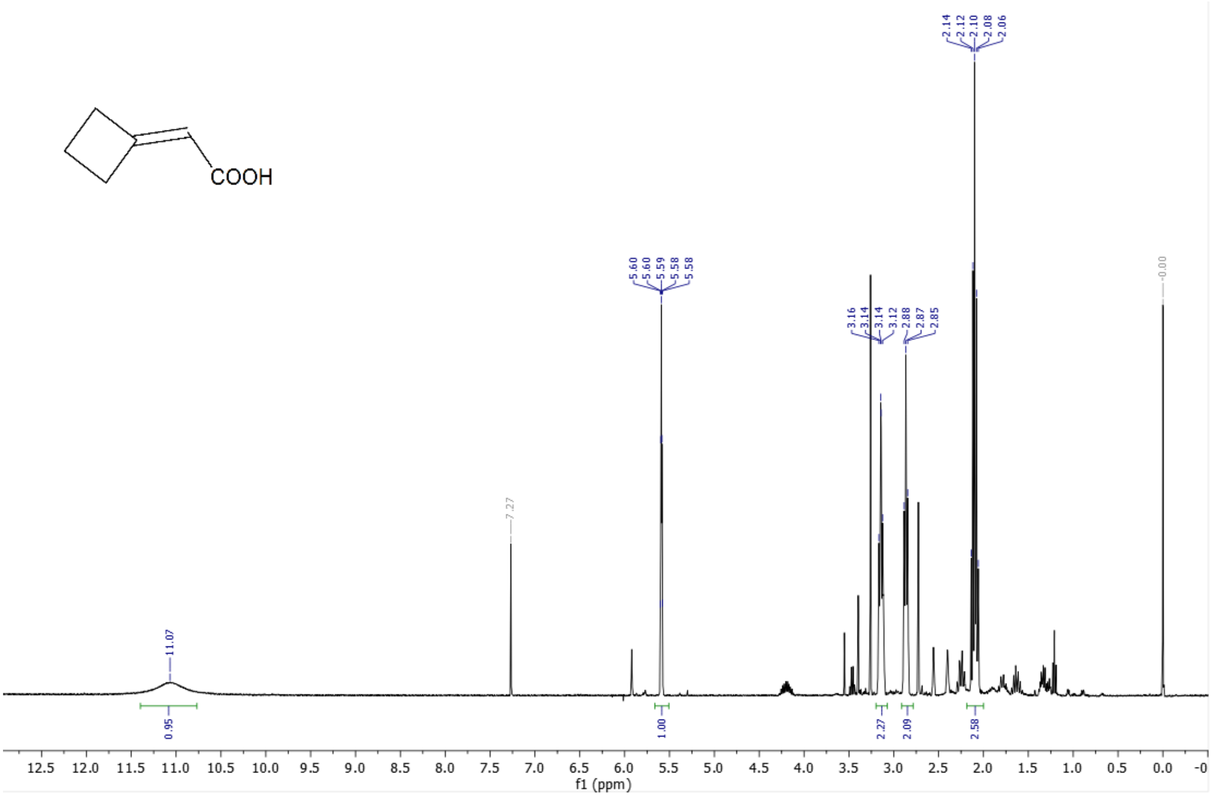

^{1}H-NMR spectrum of cyclopentylideneacetic acid (**3a**):

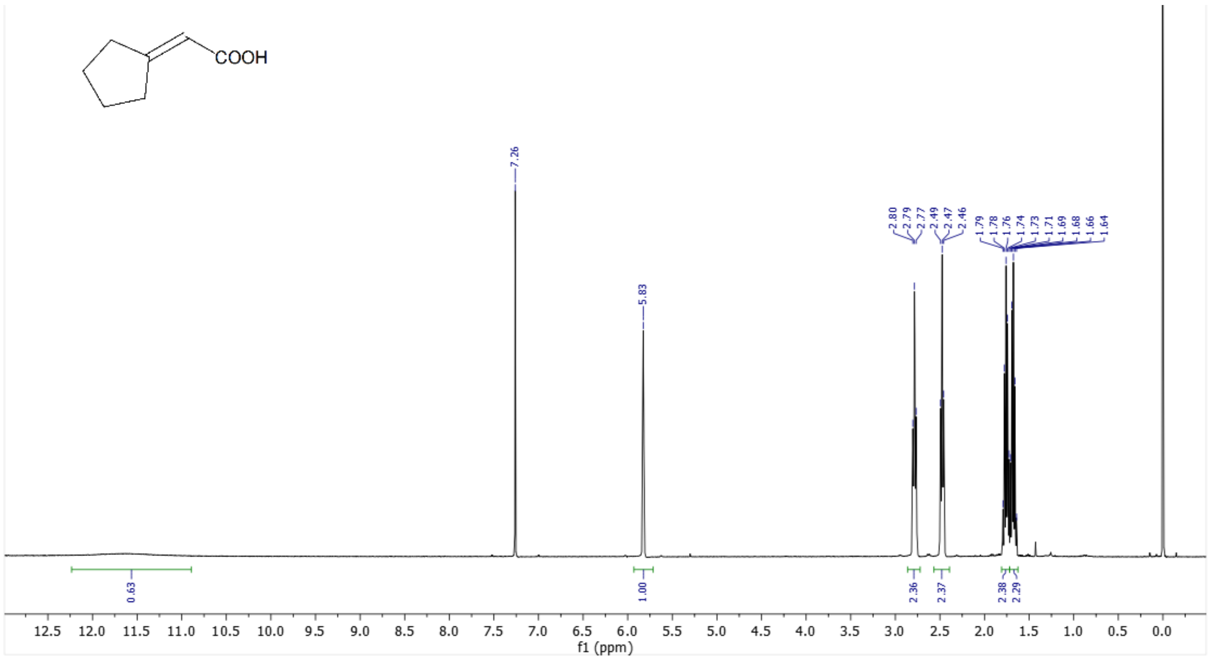

^{1}H-NMR spectrum of 2-cyclopentenylacetic acid (**3b**):

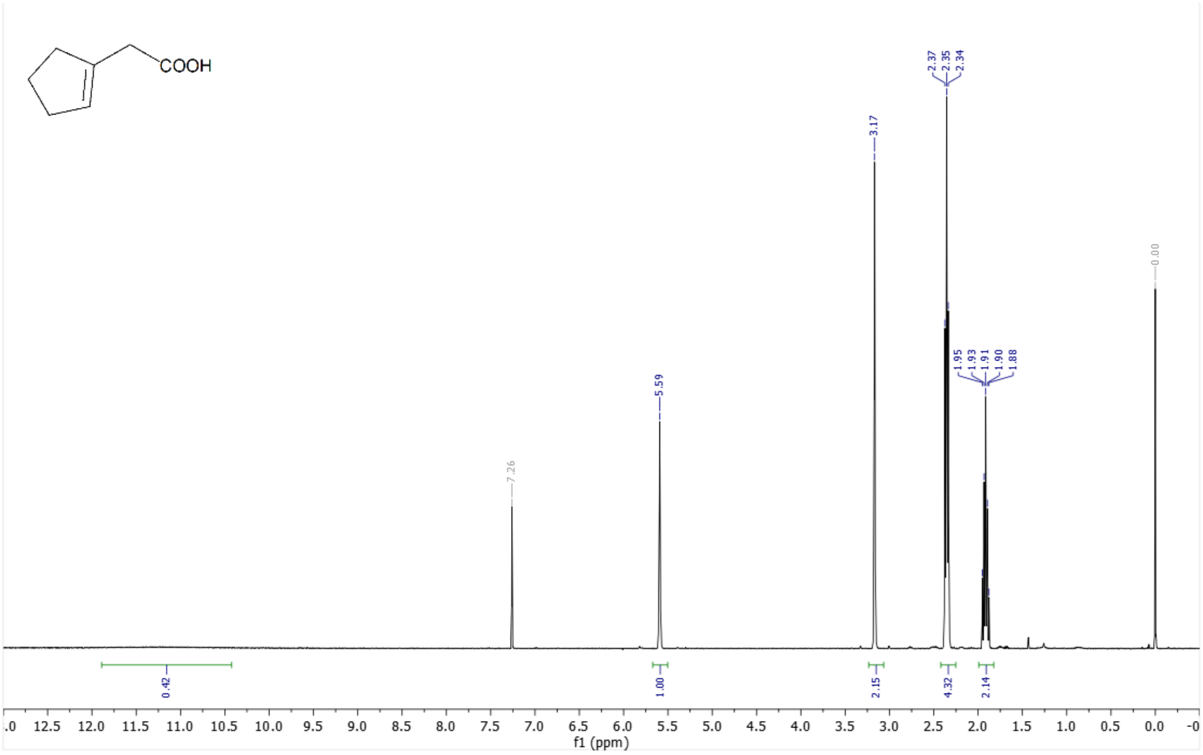

^{1}H-NMR spectrum of cyclohexylideneacetic acid (**4a**):

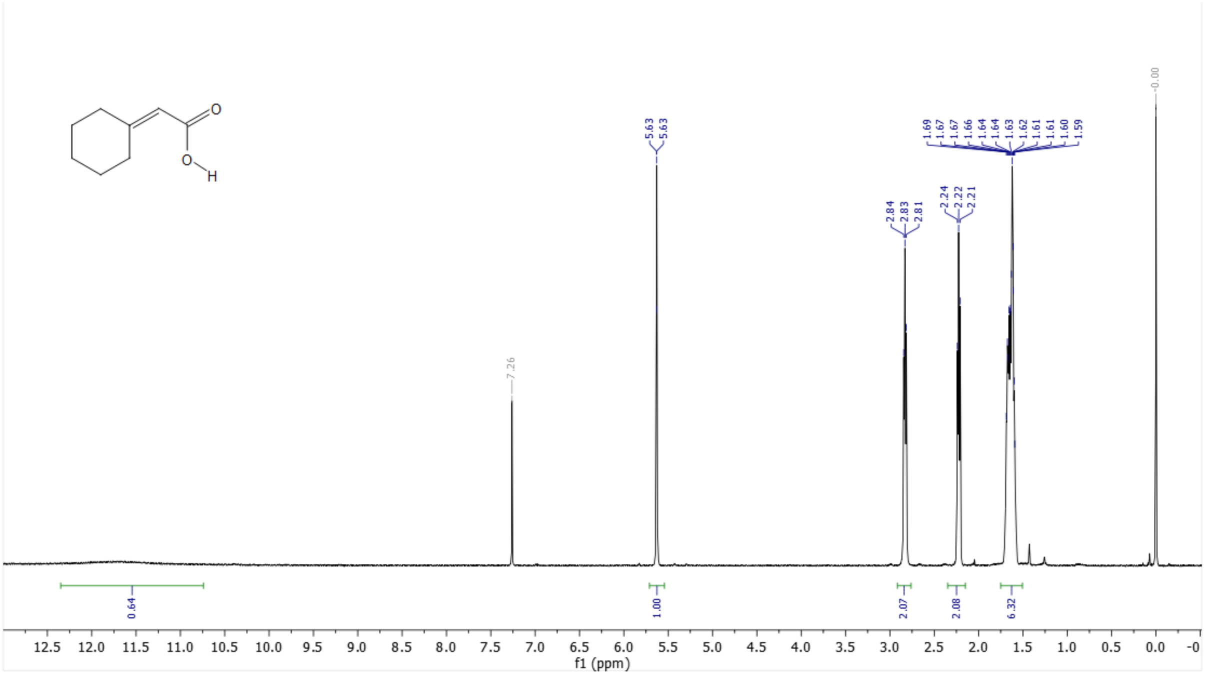

^{1}H-NMR spectrum of cycloheptylideneacetic acid (**5a**):

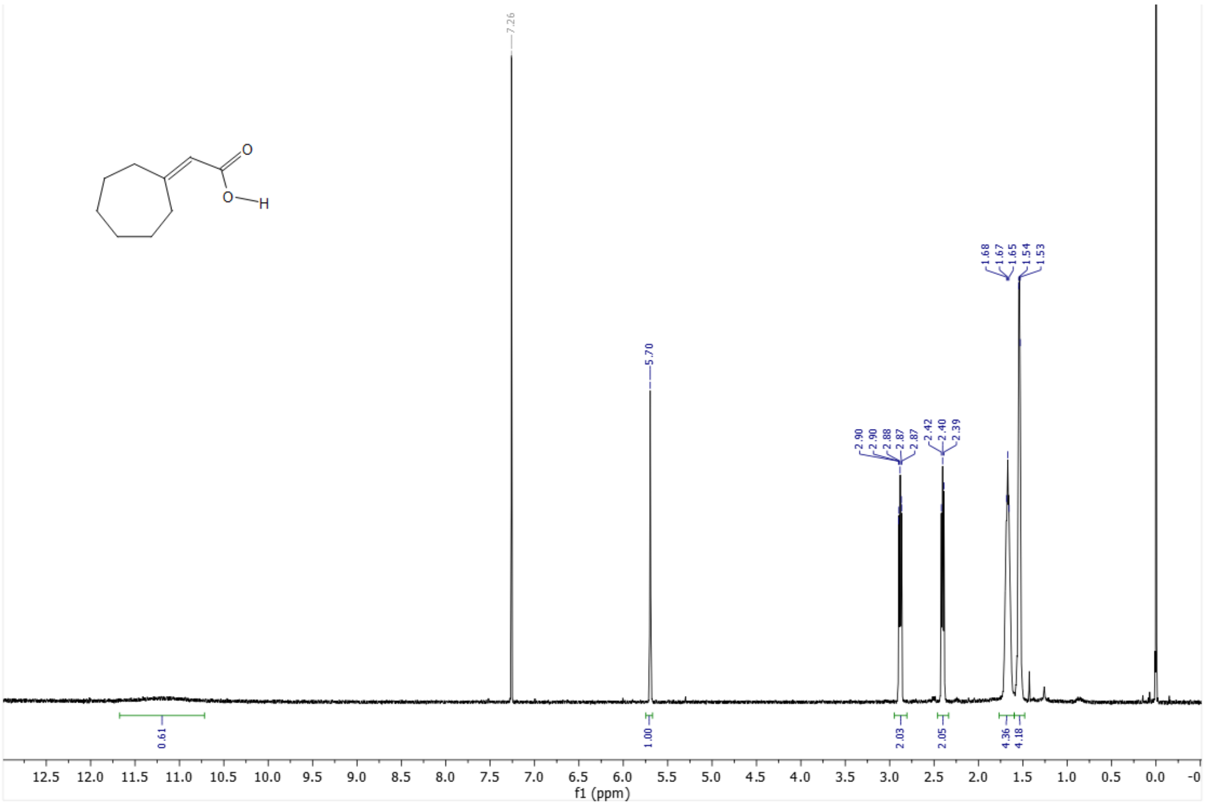

^{1}H-NMR spectrum of cyclobutylacetic acid (**7a**):

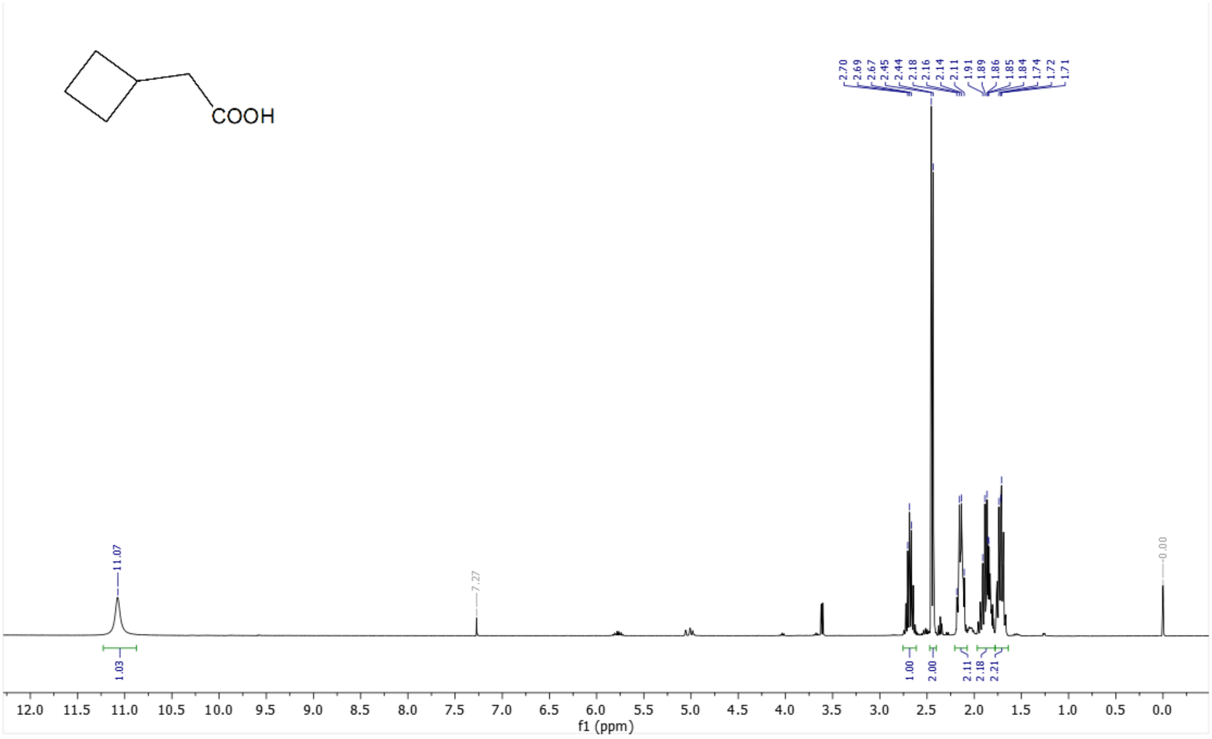

5. HPLC runs of tested compounds

HPLC analyses were carried out on a Dionex Ultimate 3000 UHPLC. Stationary phase: Kinetex C18 column (2.6 µm, 100 × 2.10mm) (Phenomenex Inc., Torrance, CA, USA). Mobile phase: Water (A) and acetonitrile (B); gradient: 0-45 min: 55-100 % B; flow rate: 0.2 mL/min; column temperature: 30 °C; wavelength: 500 nm.

cpd

2

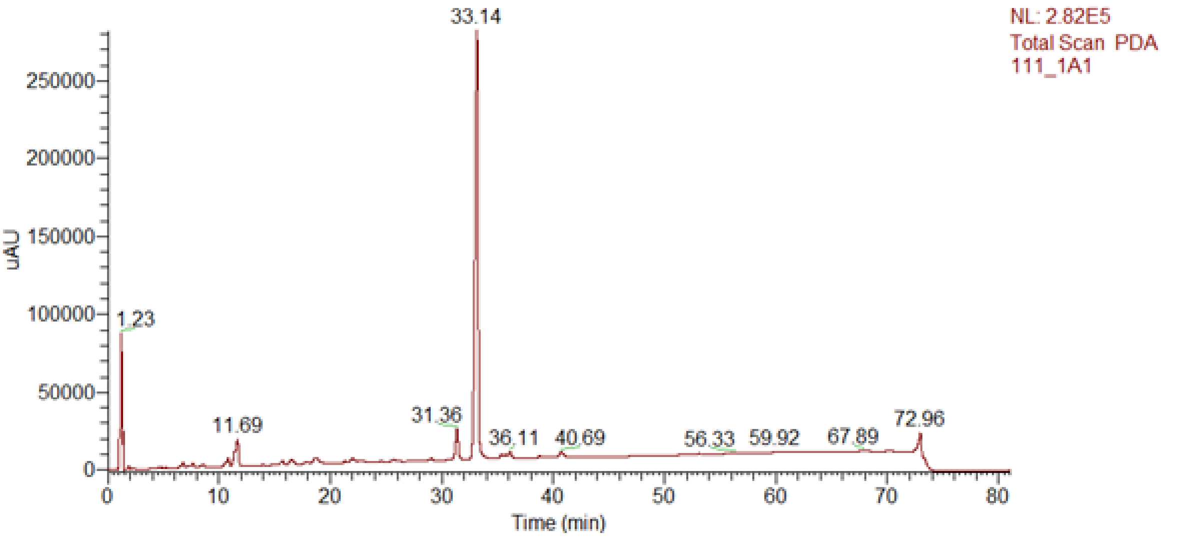

3

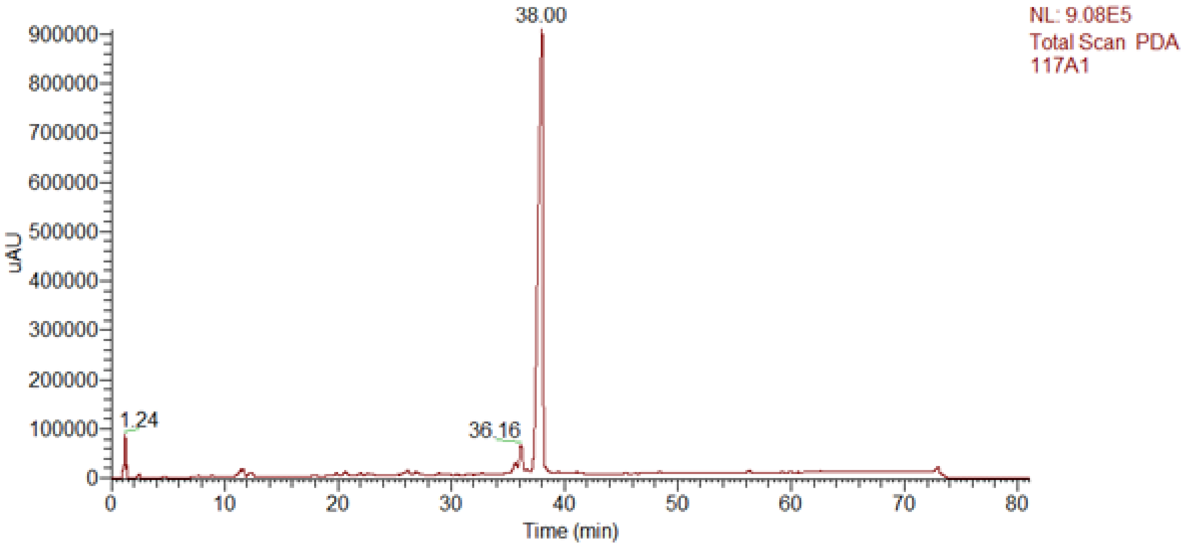

4

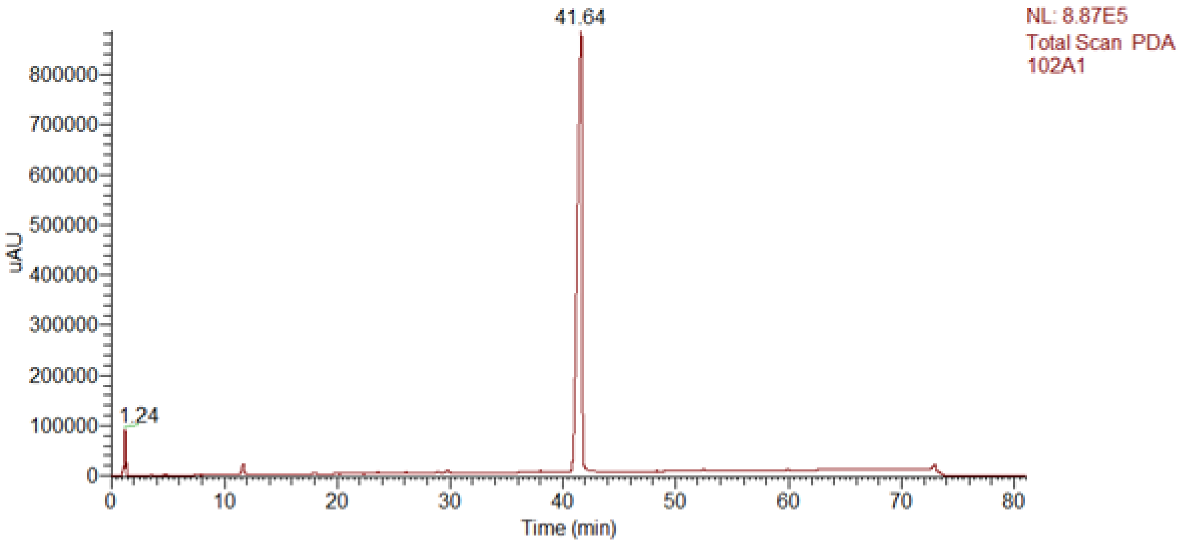
41.64
NL: 8.87E5
Total Scan PDA
102A1
800000
700000
600000
500000
400000
300000
200000
100000
0
uAU
1.24
0
10
20
30
40
50
60
70
80
Time (min)

5

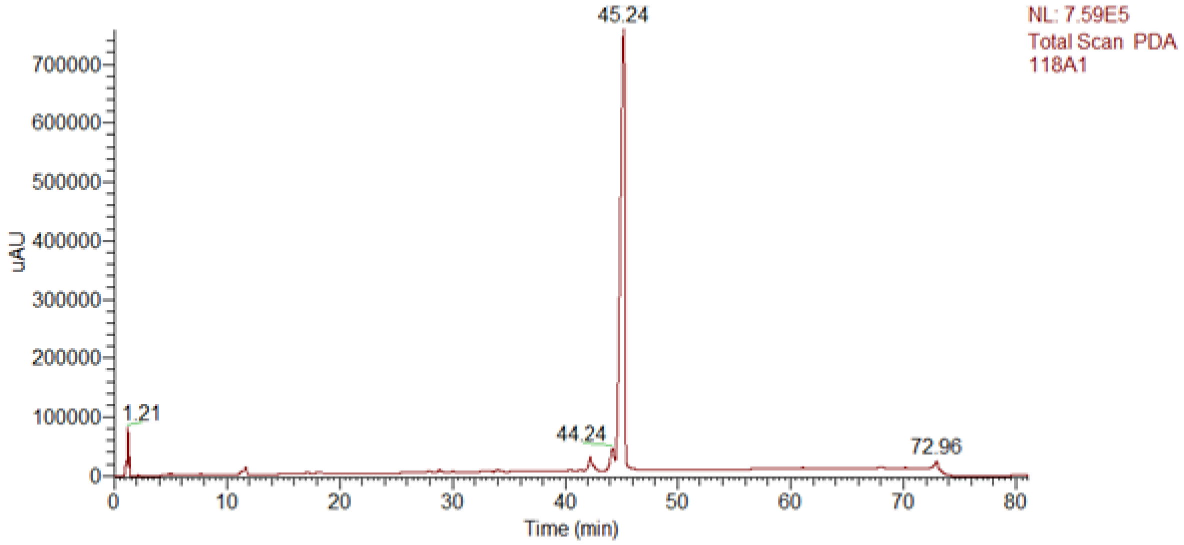
45.24
NL: 7.59E5
Total Scan PDA
118A1
700000
600000
500000
400000
300000
200000
100000
0
uAU
1.21
44.24
72.96
0
10
20
30
40
50
60
70
80
Time (min)

6

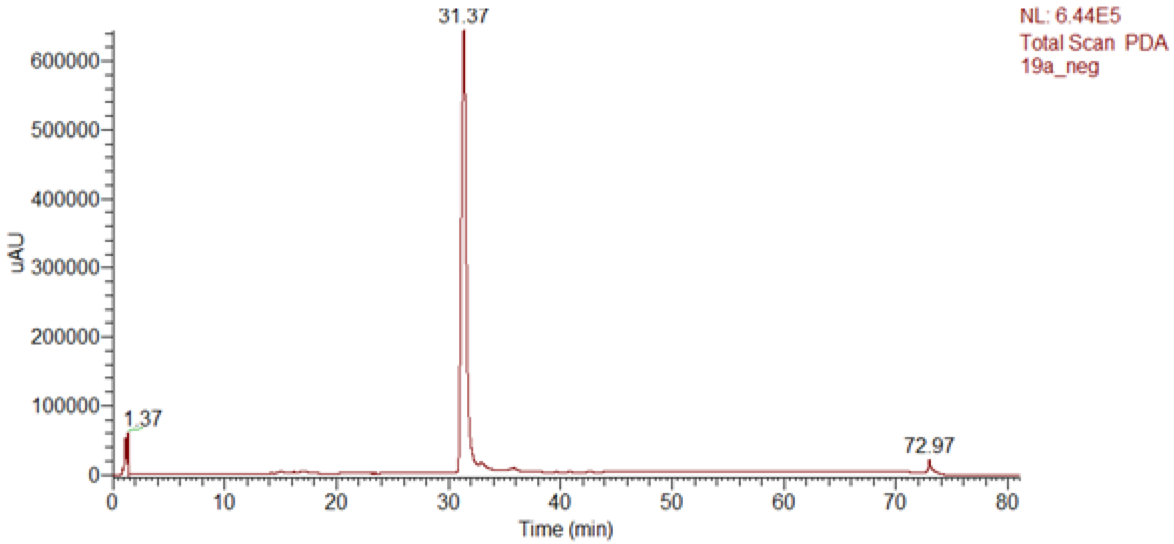
31.37
NL: 6.44E5
Total Scan PDA
19a_neg
600000
500000
400000
300000
200000
100000
0
uAU
1.37
72.97
0
10
20
30
40
50
60
70
80
Time (min)

7

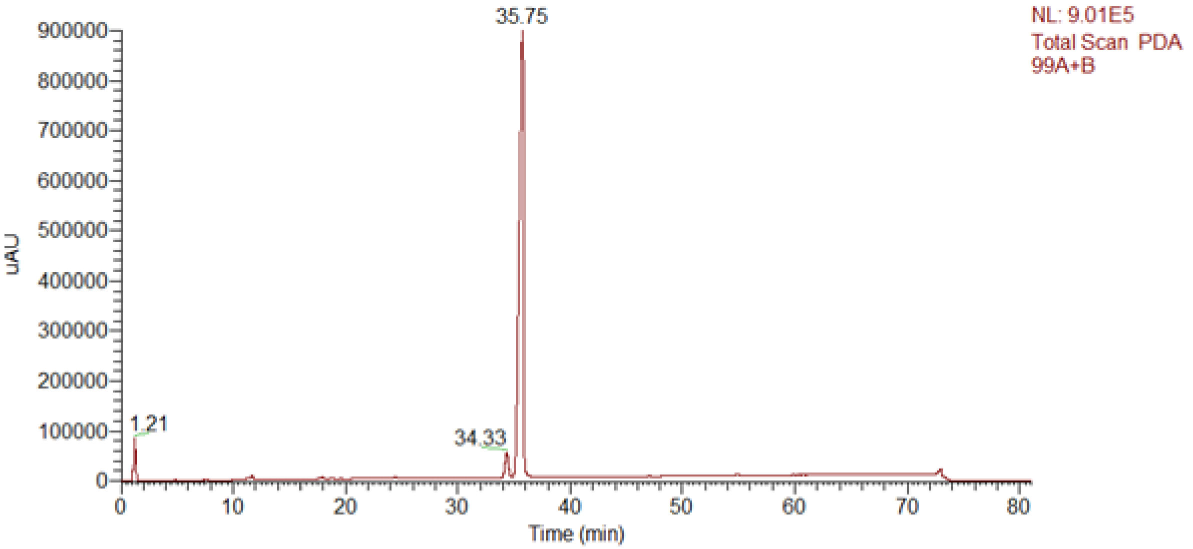
35.75
NL: 9.01E5
Total Scan PDA
99A+B
uAU
1.21
34.33
Time (min)

8

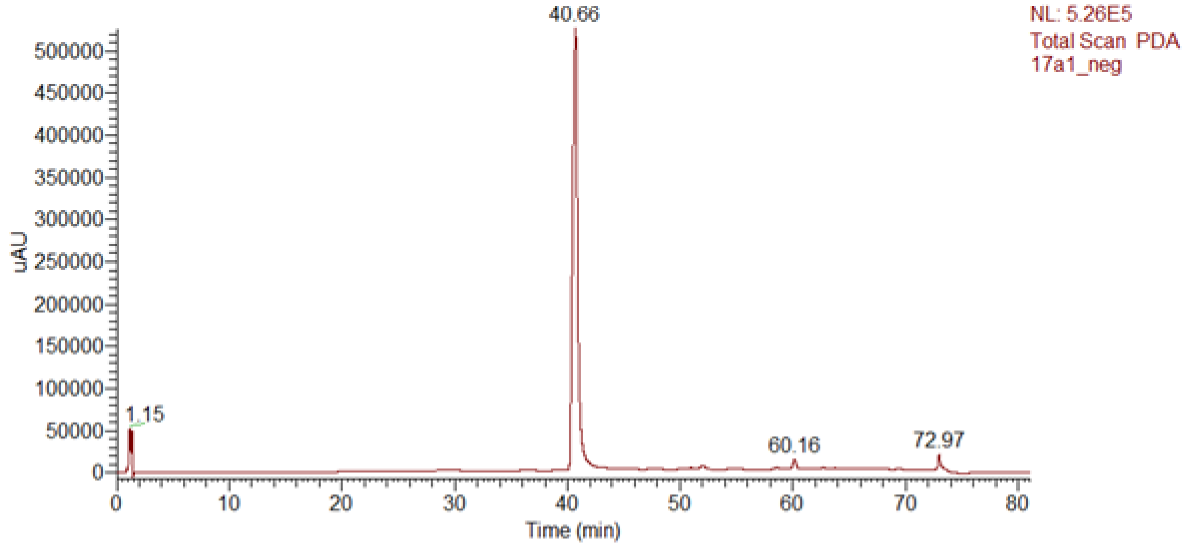
40.66
NL: 5.26E5
Total Scan PDA
17a1_neg
uAU
1.15
60.16
72.97
Time (min)

9

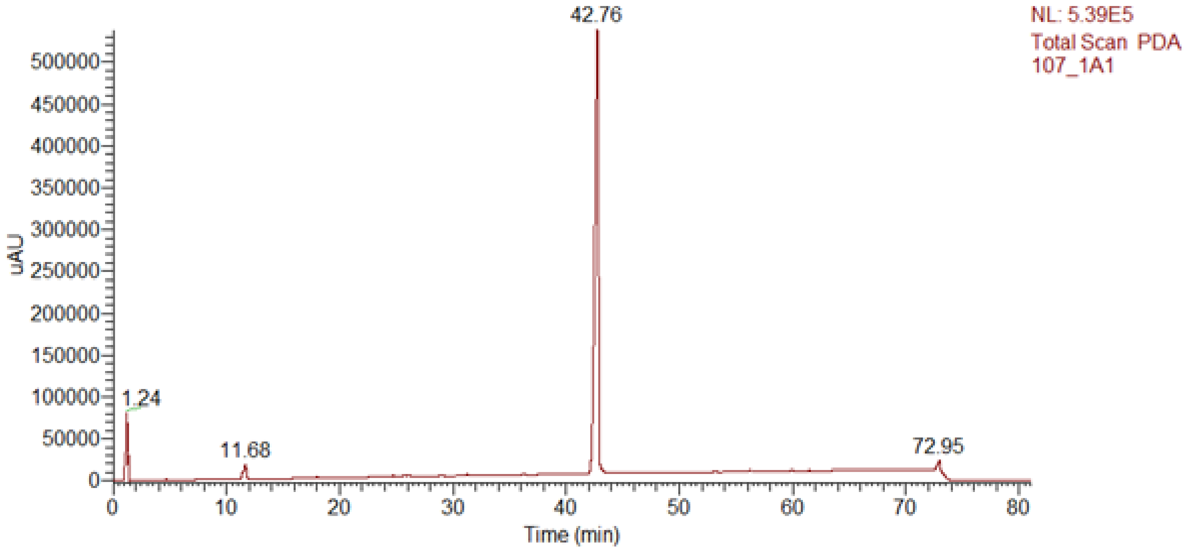
42.76
NL: 5.39E5
Total Scan PDA
107_1A1
uAU
1.24
11.68
72.95
Time (min)

10

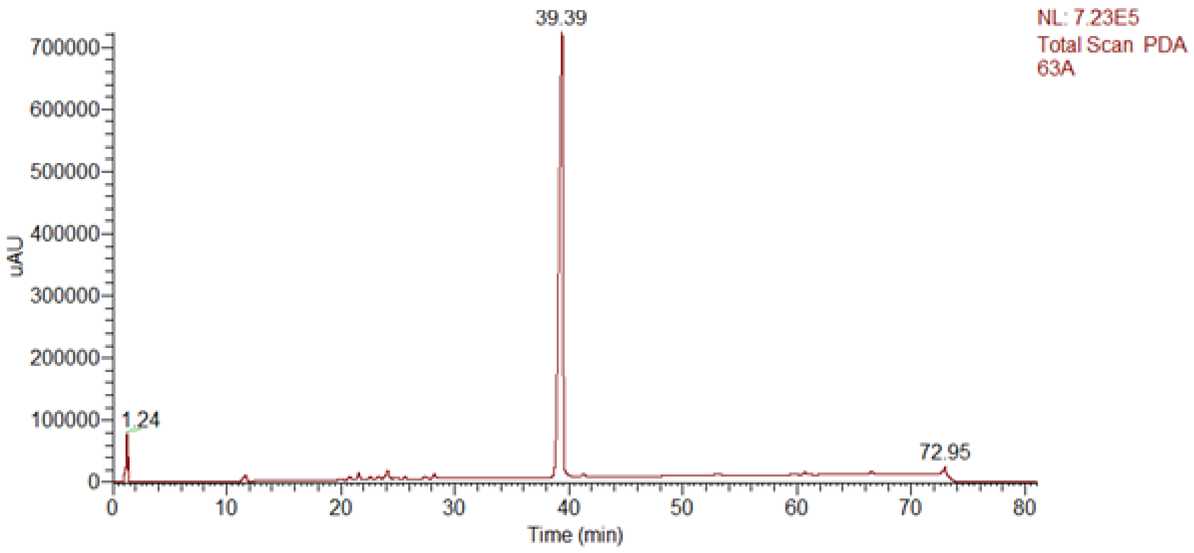
39.39
NL: 7.23E5
Total Scan PDA
63A
700000
600000
500000
400000
uAU
300000
200000
100000
1.24
72.95
0
0
10
20
30
40
50
60
70
80
Time (min)

11

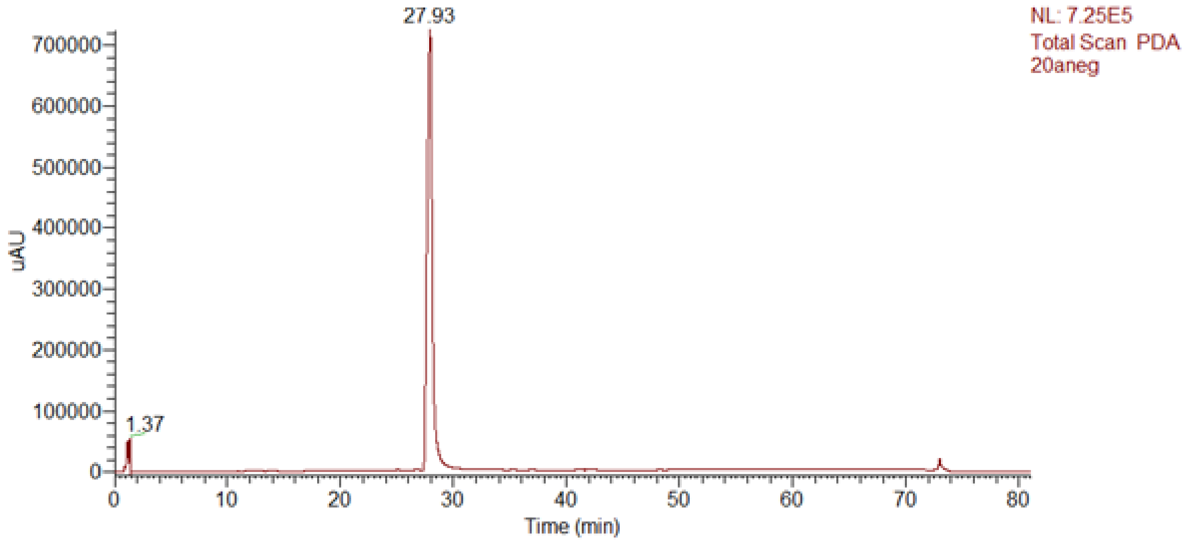
27.93
NL: 7.25E5
Total Scan PDA
20aneg
700000
600000
500000
400000
uAU
300000
200000
100000
1.37
0
0
10
20
30
40
50
60
70
80
Time (min)

12

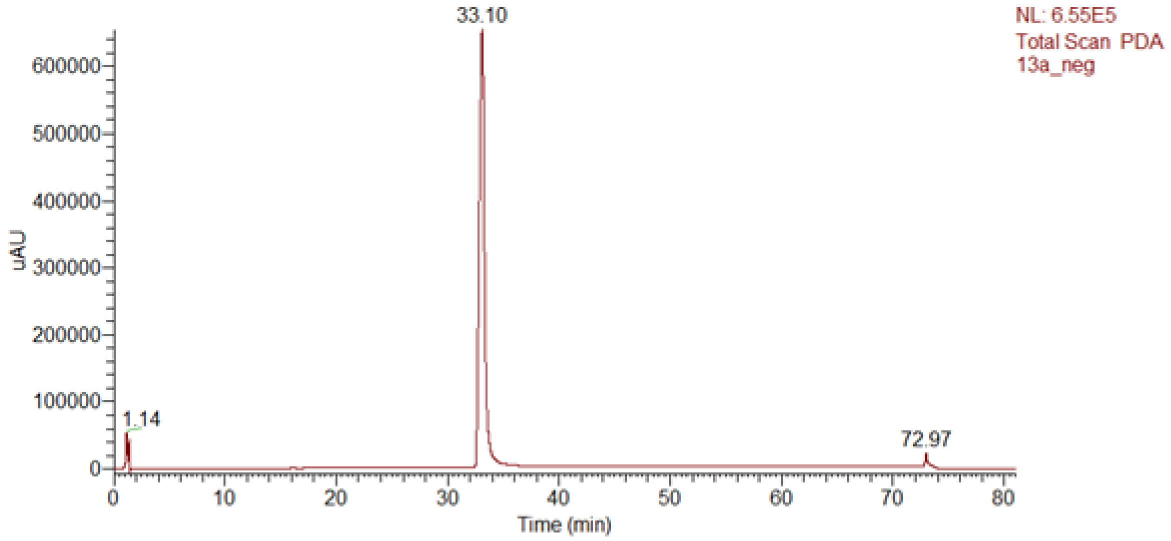
33.10
NL: 6.55E5
Total Scan PDA
13a_neg
600000
500000
400000
uAU
300000
200000
100000
1.14
72.97
0
0
10
20
30
40
50
60
70
80
Time (min)

13

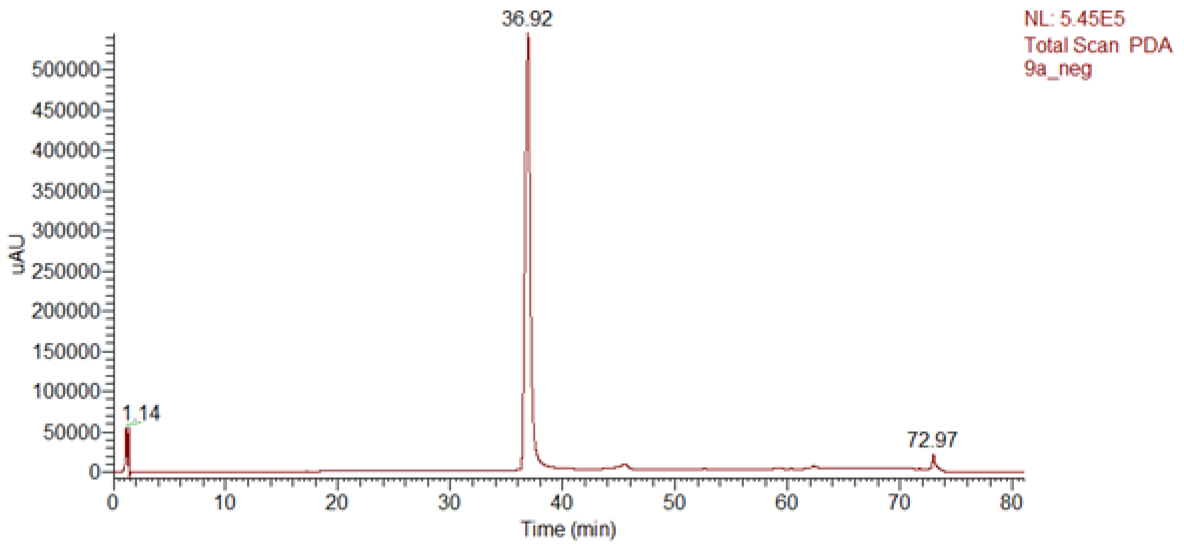
36.92
NL: 5.45E5
Total Scan PDA
9a_neg
uAU
1.14
72.97
Time (min)

14

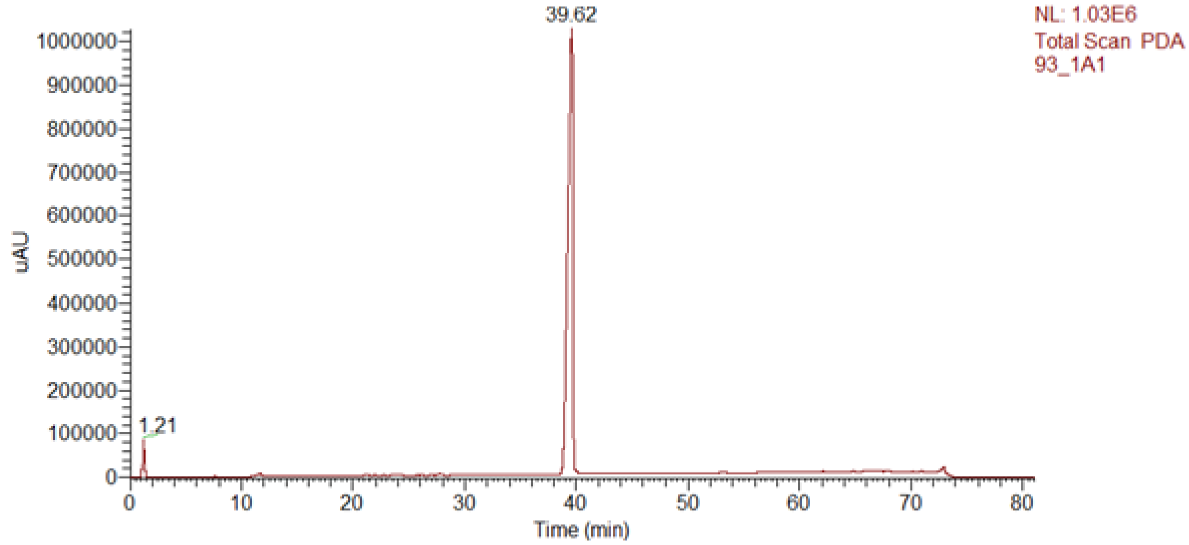
39.62
NL: 1.03E6
Total Scan PDA
93_1A1
uAU
1.21
Time (min)

15

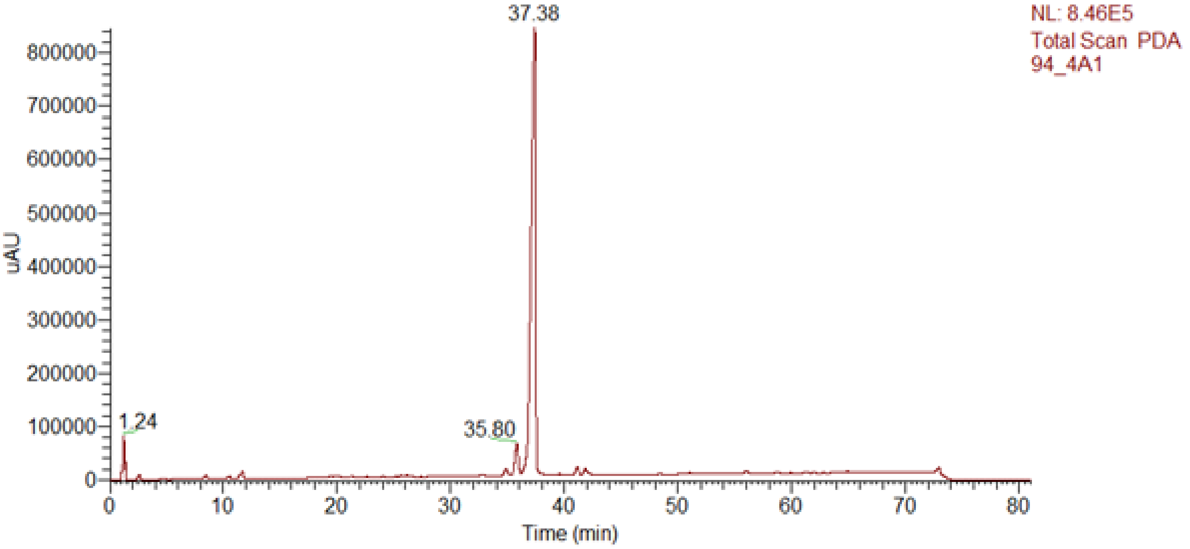
37.38
NL: 8.46E5
Total Scan PDA
94_4A1
uAU
1.24
35.80
Time (min)

16

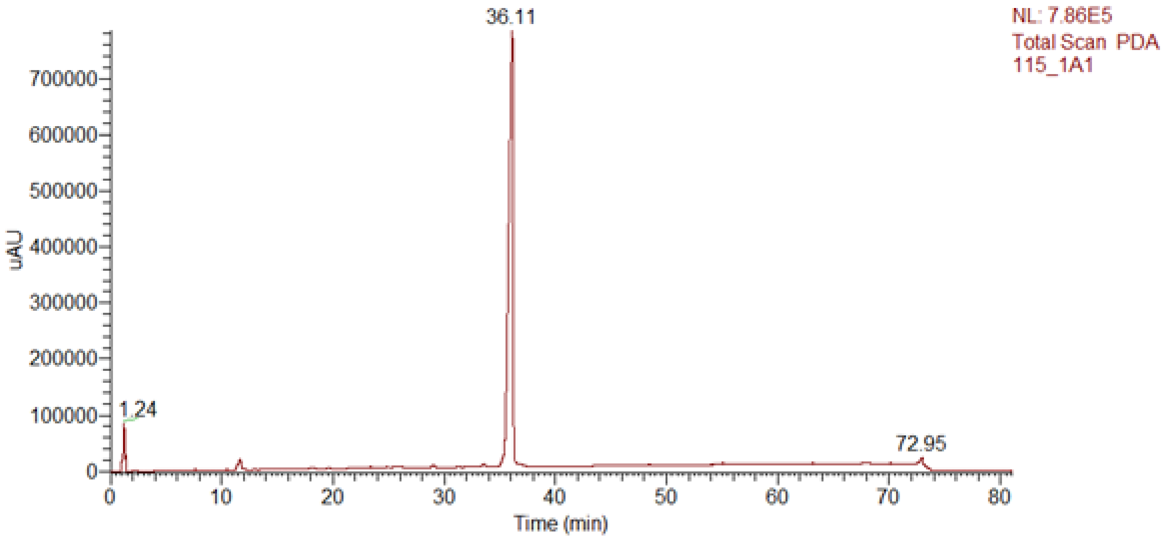
36.11
NL: 7.86E5
Total Scan PDA
115_1A1
uAU
700000
600000
500000
400000
300000
200000
100000
0
1.24
72.95
0
10
20
30
40
50
60
70
80
Time (min)

17

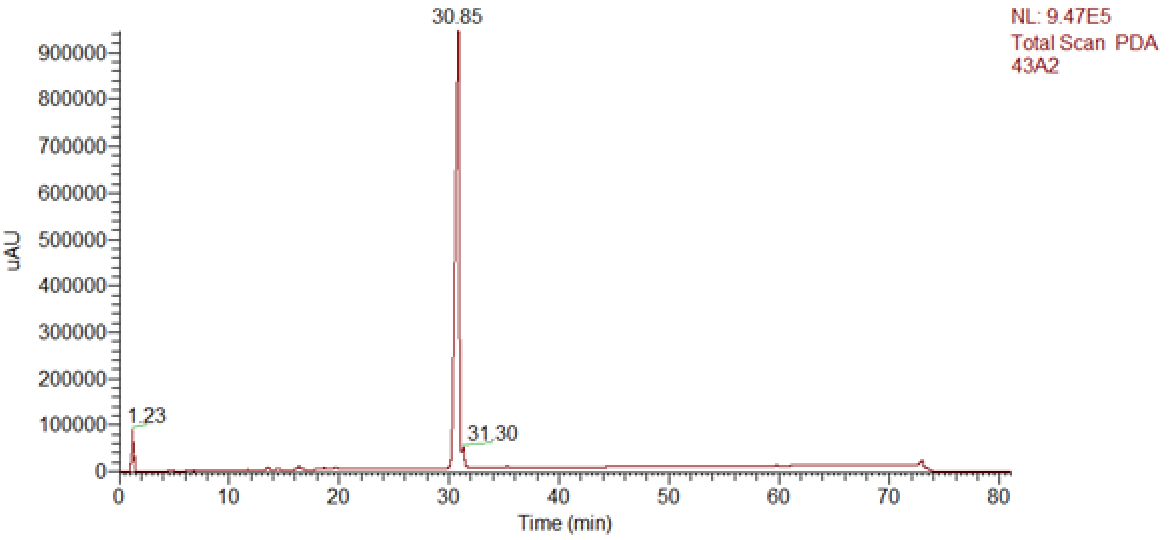
30.85
NL: 9.47E5
Total Scan PDA
43A2
uAU
900000
800000
700000
600000
500000
400000
300000
200000
100000
0
1.23
31.30
0
10
20
30
40
50
60
70
80
Time (min)

18

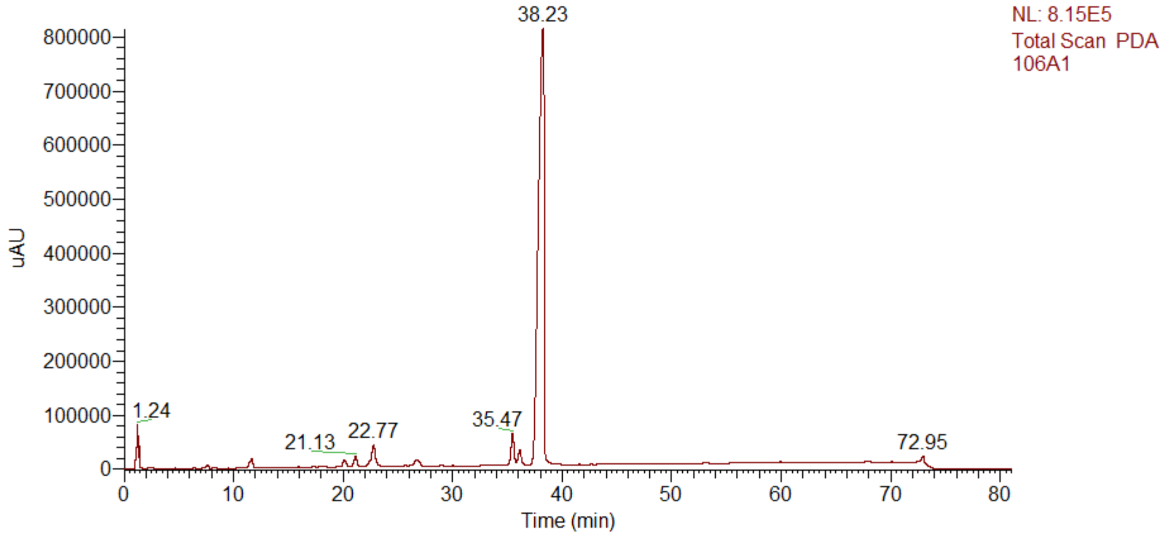
38.23
NL: 8.15E5
Total Scan PDA
106A1
uAU
800000
700000
600000
500000
400000
300000
200000
100000
0
1.24
21.13
22.77
35.47
72.95
0
10
20
30
40
50
60
70
80
Time (min)

19

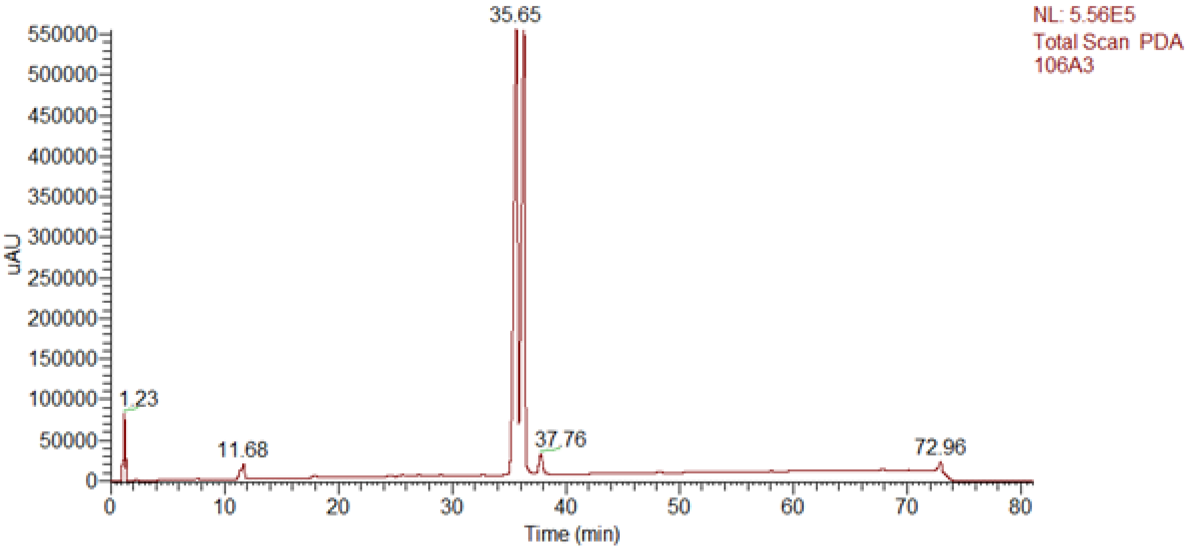
35.65
NL: 5.56E5
Total Scan PDA
106A3
uAU
550000
500000
450000
400000
350000
300000
250000
200000
150000
100000
50000
0
1.23
11.68
37.76
72.96
0
10
20
30
40
50
60
70
80
Time (min)

20

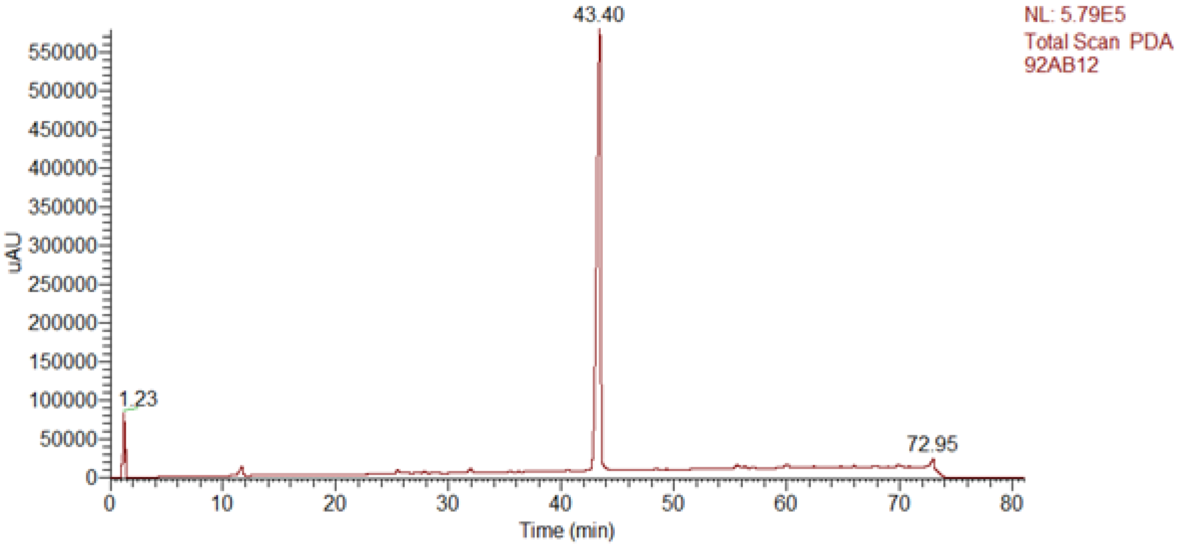
43.40
NL: 5.79E5
Total Scan PDA
92AB12
uAU
550000
500000
450000
400000
350000
300000
250000
200000
150000
100000
50000
0
1.23
72.95
0
10
20
30
40
50
60
70
80
Time (min)

6. Results of the XTT assay of compounds 1-20

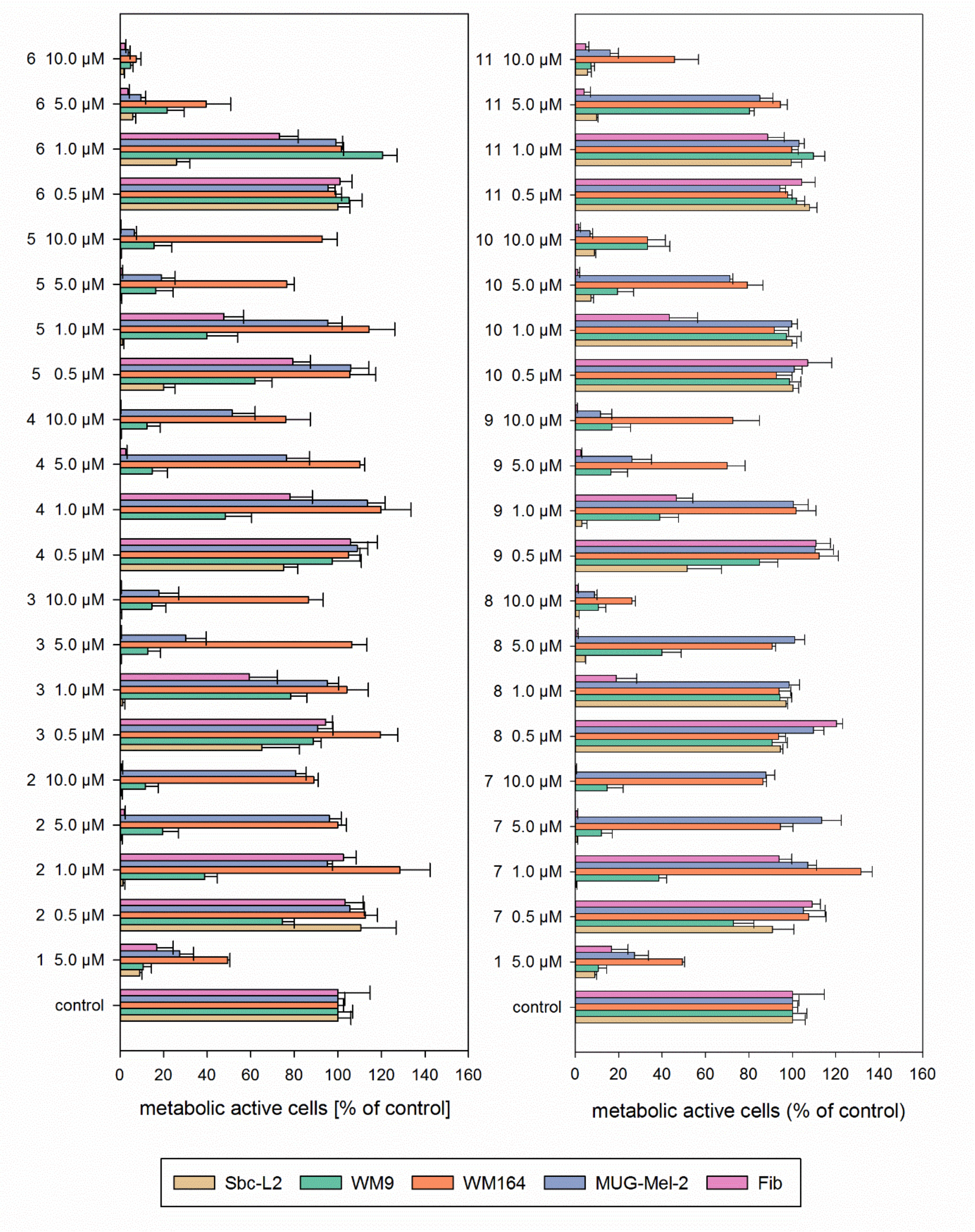

Figure S2. Results of the XTT assay of different concentrations of compounds **2**-**11** towards melanoma cells from non-metastatic (SBcl2) and metastatic lesions (WM9, WM164, MUG-Mel2) and skin fibroblasts (Fib) after 72 h of treatment (mean ± SEM, n = 4) and in comparison to 5.0 µM of **1**.

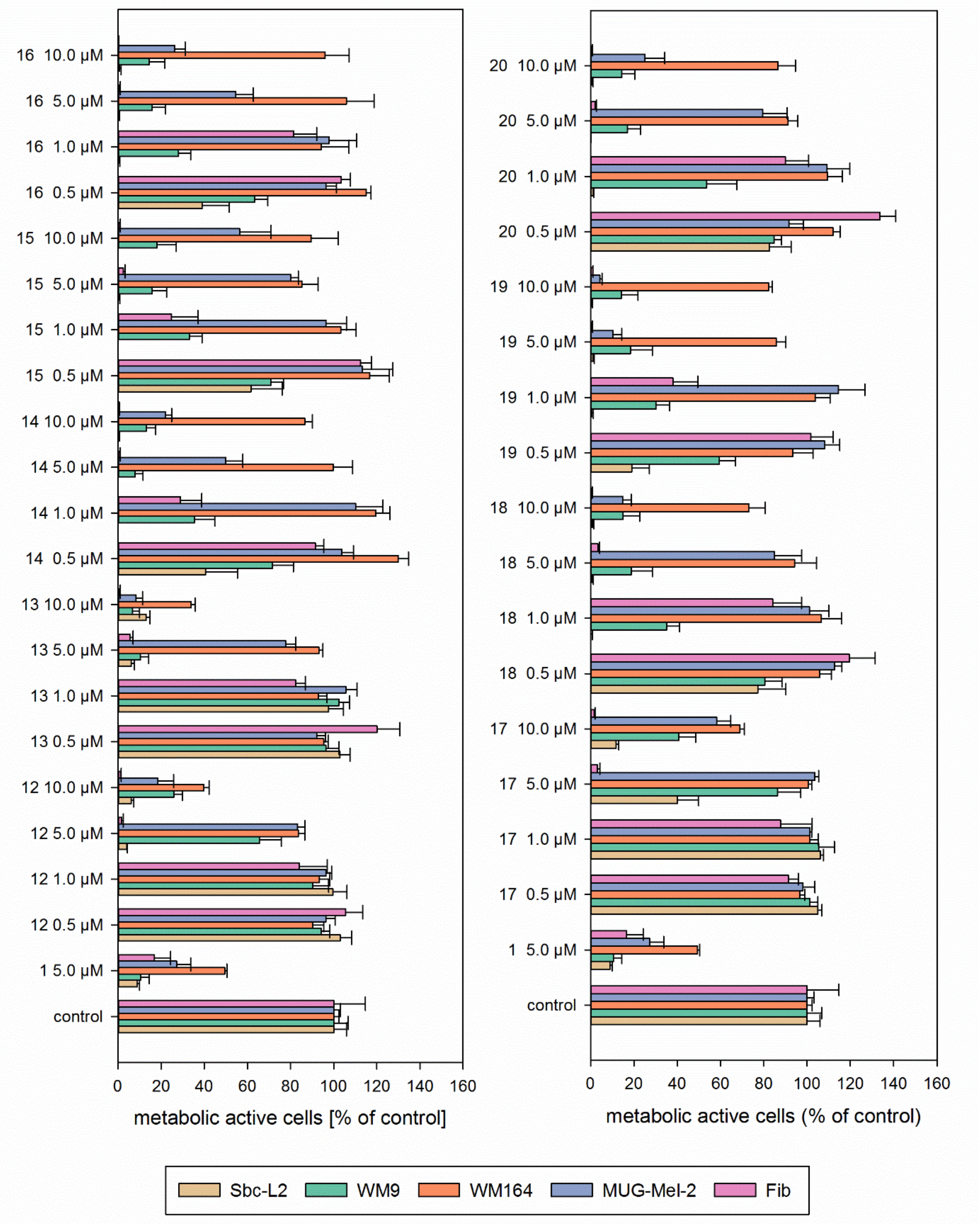

Figure S3. Results of the XTT assay of different concentrations of compounds **12-20** towards melanoma cells from non-metastatic (SBcl2) and metastatic lesions (WM9, WM164, MUG-Mel2) and skin fibroblasts (Fib) after 72 h of treatment (mean ± SEM, n = 4) and in comparison to 5.0 µM of **1**.
